# An integrated analysis of Maglemose bone points reframes the Early Mesolithic of Southern Scandinavia

**DOI:** 10.1038/s41598-020-74258-8

**Published:** 2020-10-14

**Authors:** Theis Zetner Trolle Jensen, Arne Sjöström, Anders Fischer, Erika Rosengren, Liam Thomas Lanigan, Ole Bennike, Kristine Korzow Richter, Kurt Joseph Gron, Meaghan Mackie, Morten Fischer Mortensen, Lasse Sørensen, David Chivall, Katrine Højholt Iversen, Alberto John Taurozzi, Jesper Olsen, Hannes Schroeder, Nicky Milner, Mikkel Sørensen, Matthew James Collins

**Affiliations:** 1 Section for Evolutionary Genomics, Faculty of Health and Medical Sciences, GLOBE Institute, University of Copenhagen, Øster Farimagsgade 5, 1353 Copenhagen, Denmark; 2 Department of Archaeology, BioArCh, University of York, Environment Building, Wentworth Way, York, YO10 5NG UK; 3 Department of Archaeology and Ancient History, Lund University, Helgonavägen 3, Box 192, 221 00 Lund, Sweden; 4 Sealand Archaeology, Gl. Roesnaesvej 27, 4400 Kalundborg, Denmark; 5 Historical Museum, Lund University, Krafts Torg 1, 223 50 Lund, Sweden; 6 Geological Survey of Denmark and Greenland (GEUS), Øster Voldgade 10, Copenhagen K, Denmark; 7 Department of Archaeology, Max Planck Institute for the Science of Human History, Kahlaische Strasse 10, 07745 Jena, Germany; 8 Department of Archaeology, Durham University, South Road, Durham, DH1 3LE UK; 9 Novo Nordisk Foundation Center for Protein Research, University of Copenhagen, Blegdamsvej 3b, 2200 Copenhagen N, Denmark; 10 Department of Environmental Archaeology and Materials Science, The Danish National Museum, I.C.Modewegsvej, 2800 Lyngby, Denmark; 11 The Danish National Museum, Ancient Cultures of Denmark and the Mediterranean, Ny Vestergade 10, Prinsens Palæ, 1471 København K, Denmark; 12 Oxford Radiocarbon Accelerator Unit (ORAU), School of Archaeology, University of Oxford, 1 South Parks Road, Oxford, OX1 3TG UK; 13 Department of Health Technology, Technical University of Denmark, Kemitorvet, 2800 Kgs. Lyngby, Denmark; 14 Department of Physics and Astronomy, Aarhus AMS Centre (AARAMS), Aarhus University, Ny Munkegade 120, 8000 Aarhus C, Denmark; 15 Department of Archaeology, University of York, King’s Manor, York, YO1 7EP UK; 16 Department of Archaeology, The Saxo Institute, University of Copenhagen, Karen Blixens plads 8, 2300 Copenhagen S, Denmark; 17 McDonald Institute for Archaeological Research, University of Cambridge, West Tower, Downing St, Cambridge, CB2 3ER UK

**Keywords:** Zoology, Evolutionary ecology, Proteomics, Environmental impact

## Abstract

The extensive peat bogs of Southern Scandinavia have yielded rich Mesolithic archaeological assemblages, with one of the most iconic artefacts being the bone point. Although great in number they remain understudied. Here we present a combined investigation of the typology, protein-based species composition, and absolute chronology of Maglemosian bone points. The majority of the bone points are made from cervids and bovines. However, changes both in species composition and barb morphology can be directly linked to a paucity of finds lasting nearly 600 years in Southern Scandinavia around 10,300 cal BP. We hypothesize that this hiatus was climate-driven and forced hunter-gatherers to abandon the lakes. Furthermore, the marked change in bone points coincides with a change in lithic technology. We, therefore, propose that the Maglemose culture in Southern Scandinavia is fundamentally divided into an Early Complex and a Late Complex.

## Introduction

The biological and geological record of the transition from the Late Glacial to the Early Holocene is manifested by a dramatic change in vegetation due to climatic warming^1^. With the increase in temperature vast amounts of buried stagnant ice gradually melted forming water-filled depressions. The Early Holocene landscape was therefore characterized by numerous shallow lakes and ponds in a relatively open birch and pine-dominated woodland^2^. These formed crucial hunting and fishing grounds for the first Maglemosian people living in Southern Scandinavia.

Throughout the Early Mesolithic barbed bone points were frequently lost in the lakes, presumably in connection with spearfishing. These lakes gradually evolved into bogs where peat accumulated. Fuel shortages, especially during the First and Second World Wars, resulted in industrialized peat exploitation in these bogs, which in turn caused these artefacts to be uncovered and recognized for what they are^3–5^.

The barbed bone points soon became closely associated with the Maglemose culture (c. 11,000–8000 BP). However, despite a long history of research on the typology of these characteristic items^6–8^, their chronological placement has largely been indirectly deduced from stratigraphy and pollen dating^4,9^. Species identification of bone points has previously been based on such evidence as bone debitage from habitation sites, or the absence of specific skeletal elements in a given faunal assemblage^3,10^^, p. 278,^^11^, which makes the identification of each artefact circumstantial. Based on these types of analyses, the majority of the Maglemosian bone points were thought to have been made from both ribs and long bones of “large ungulates”, translating to aurochs, elk, red- and roe deer (*Bos primigenius, Alces alces, Cervus elaphus,* and *Capreolus capreolus*)^12^.

**Figure 1:**
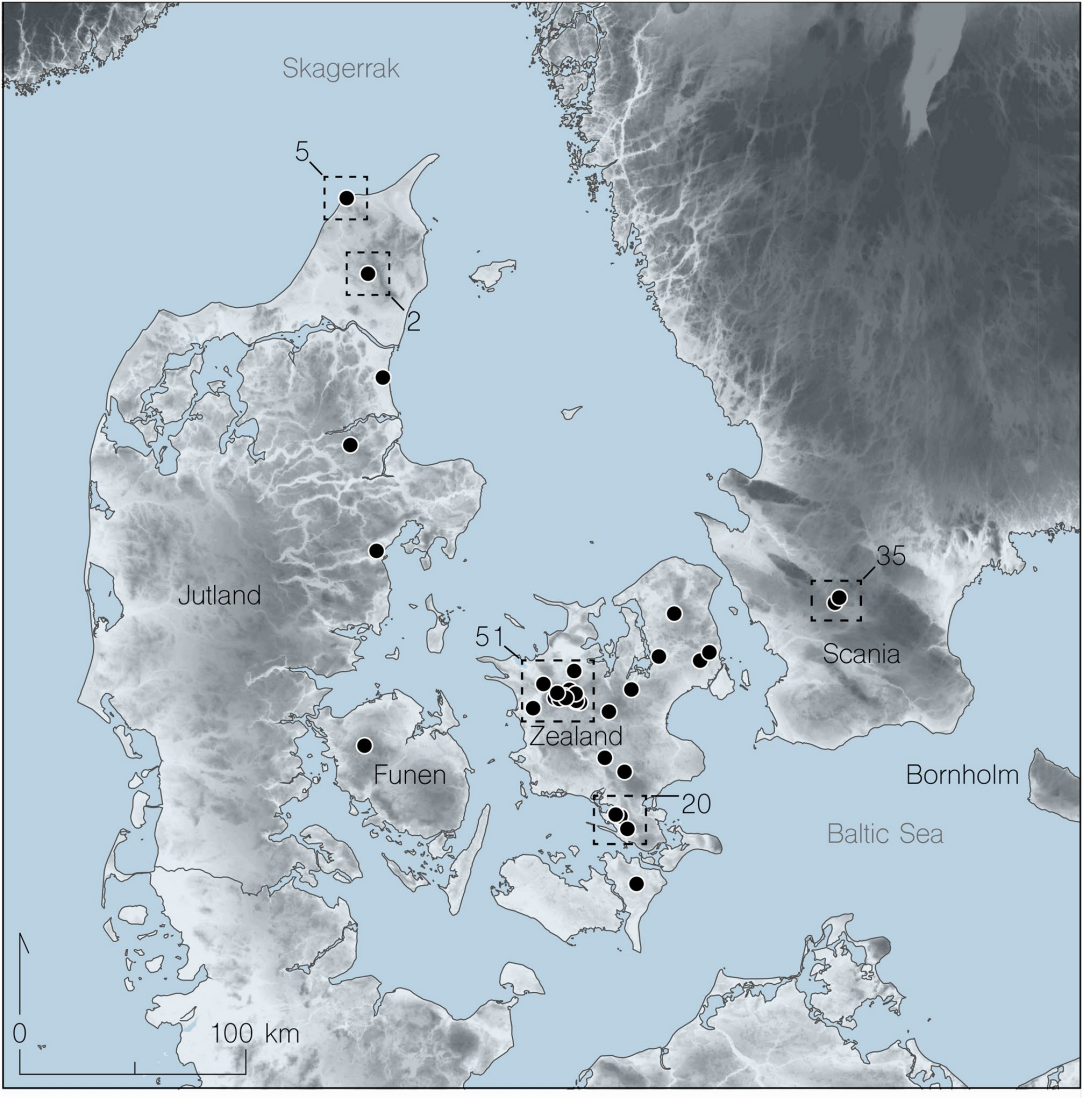
Overview of the approximate find locations for the 126 barbed bone points from Southern Scandinavia. All bone points found in paleolake systems, now grown into peat bogs. While the selection is finite the spread indicates the approximate Eastern → Western extent of sediments conducive for preservation (for further information on each artefact including species identifications see Supplementary Dataset 1 and Supplementary Figures 1–22 and 29–30) (Map: digital elevation model was produced using open source Copernicus data and information (from the European Union—EU-DEM layers), and then merged with spatial geographical data in QGIS v.3.19 (www.qgis.org), followed by a final correction in Adobe Illustrator v. 24.3 (www.adobe.com).

We analyzed 126 bone points from Southern Scandinavia (Fig. 1 and Supplementary Information, Section 1) using a combination of morphological typology, radiocarbon dating, and proteomic analyses. Using these techniques, we were able to assess the selection of raw materials and date the typological variation. This study is the first attempt to investigate whether a single artefact type can be used as a proxy for both human and animal populations. We demonstrate that such comprehensive analyses can provide information about species dynamics, resource exploitation, human–environment interactions, and perhaps population mobility. Our integrated analyses provide a robust new framework for Maglemose chronology, that extends to changes in lithic manufacture.

## Results

### Bone points

Local bone point typo-chronologies have previously been proposed for two sites in Sweden, i.e. Rönneholms mosse^8^ and Motala^13^. However, since the present study comprises material from a much wider geographical region, this fine-scale classification of the bone points might not be representative. Therefore, to reduce bias, we divided the material into two groups; fine-barbed bone points and large-barbed bone points.

### Radiocarbon dating

**Figure 2:**
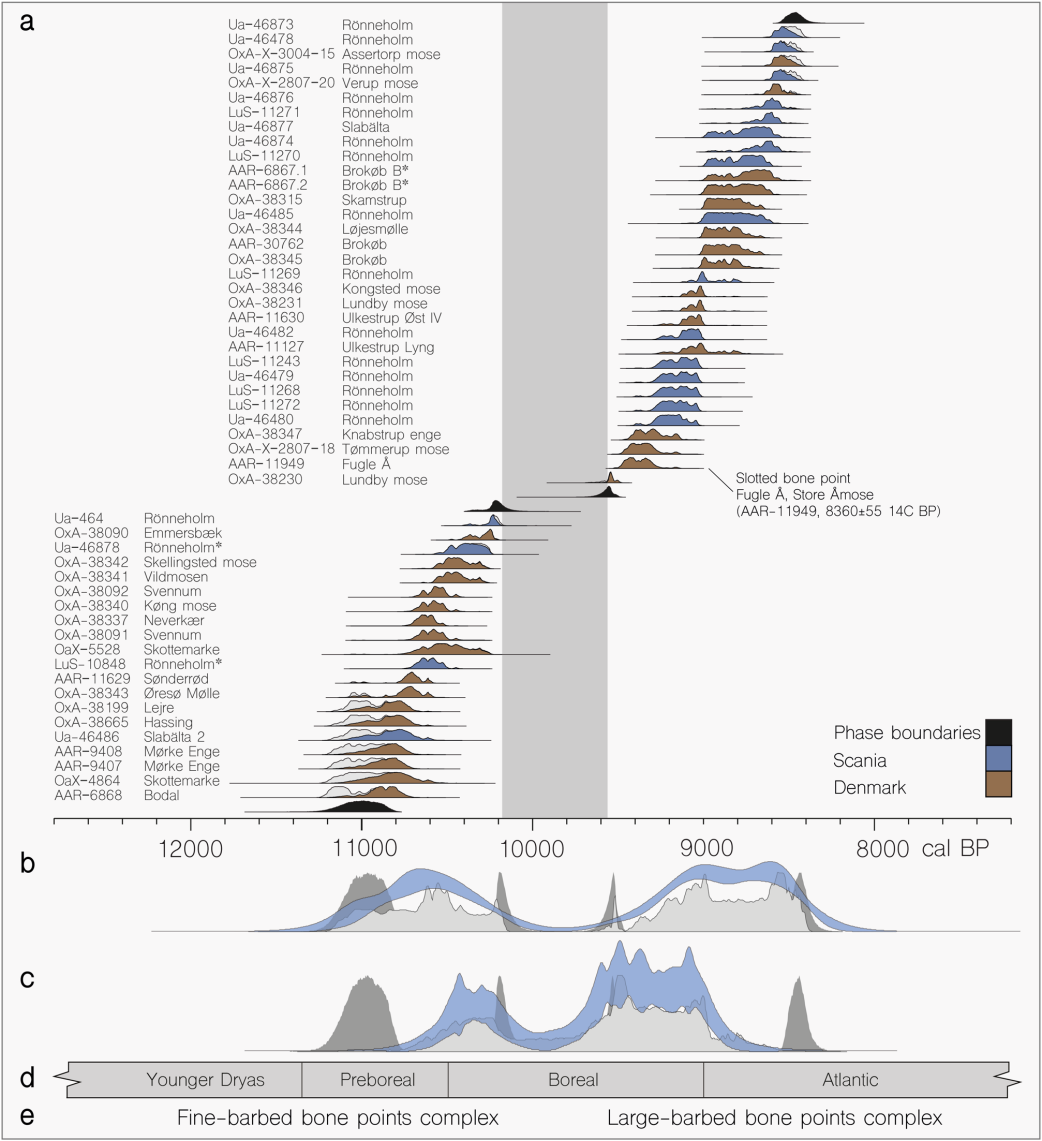
Radiocarbon dates from bone points showing the separation of the two types. (**a**) A Bayesian model assuming two phases performed on 52 bone points. Double dates on the same artefact marked with *. Carbon distributions colored to denote finding the place or phase boundaries, (**b**) Summed radiocarbon dates of bone points (shown in light grey), KDE to visualize activity and hiatus (light blue), boundaries marked in dark grey, (**c**) Summed radiocarbon dates from habitation in Denmark (see Supplementary Figure 23) (shown in light grey), KDE to visualize activity and hiatus (light blue), boundaries marked in dark grey, (**d**) biozones (Preboreal onset after Jessen et al.^1^)(**e**) the two bone point complexes separated by hiatus (see Table 1 and Supplementary Dataset 1).

Prior to this study, only five radiocarbon (^14^C) dates on Danish barbed bone points had been published^14,15^. With the addition of previously published dates from Scania in Sweden (*n* = 20)^8^, unpublished dates from Denmark (*n* = 7) as well as 21 new ^14^C measurements acquired specifically for this project, we were able to model the radiocarbon-date distribution of 50 bone points (excluding double dates on the same artefact) (Fig. 2; Table 1; Supplementary Information, section 2; Supplementary Dataset 1). After calibration into cal. years BP in OxCal v.4.3^16^, the artefacts separated into two distinct phases (Fig. 2). The fine-barbed bone points are all confined to the mid-late Preboreal and the beginning of the Boreal (c. 11,200–10,100 cal BP). The larger-barbed bone points are restricted to the end of the Boreal and the beginning of the Atlantic chronozones (9658–8413 cal BP). Between the fine-barbed bone points and the larger-barbed bone points, there is a clear gap in the radiocarbon ages lasting nearly 600 years. In order to explore this hiatus, we summed the radiocarbon dates and performed a Kernel Density Estimation (KDE) simulation to explore periods of activity. This indicates temporal and spatial morphological patterns and confirms the age correlated distributions of the two types of bone points in both regions (Fig. 2).

**Table 1:** List of 52 radiocarbon dates and stable isotope data used in this study.

Lab Nr	Site	Sample	14C years BP	Age cal. BP (95.4%)	δ^13^C	δ^15^N	CN	References
AAR-6868	Bodal	AFi I 050700	9660 ± 75	11,212–10,767	–	–	–	Fischer^15^
OxA-4864	Skottemarke	A20371	9570 ± 100	11,195–10,603	–	–	–	Fischer^14^
AAR-9407	Mørke Enge	Lyster 10	9605 ± 65	11,175–10,742	–	–	–	This study
AAR-9408	Mørke Enge	Lyster 7	9595 ± 65	11,171–10,736	–	–	–	This study
Ua-46486	Slabälta 2	Sla2	9546 ± 76	11,161–10,660	–	–	–	Larsson et al.^8^
OxA-38665	Hassing	A16966	9557 ± 63	11,143–10,695	− 22.3	+ 3.0	3.4	This study
OxA-38199	Lejre	A37811	9555 ± 55	11,125–10,702	− 21.5	+ 1.5	3.3	This study
OxA-38343	Øresø Mølle	A52426	9481 ± 47	11,070–10,584	− 24.3	+ 1.7	3.5	This study
AAR-11629	Sønderød	A44205	9468 ± 35	11,061–10,585	–	–	–	This study
LuS 10848	Rönneholms mosse*	FP1469	9375 ± 45	10,719–10,444	–	–	–	Larsson et al.^8^
OxA-5528	Skottemarke	A20364	9310 ± 90	10,717–10,252	–	–	–	Fischer^14^
OxA-38091	Svennum	VHM14923	9365 ± 45	10,709–10,438	− 20.8	+ 3.8	3.4	This study
OxA-38337	Neverkær	A39316	9391 ± 37	10,708–10,518	− 25.6	+ 3.4	3.4	This study
OxA-38340	Køng mose	534	9360 ± 44	10,703–10,436	− 20.9	+ 4.6	3.3	This study
OxA-38092	Svennum	VHM21070	9345 ± 40	10,685–10,430	− 21.7	+ 5.3	3.2	This study
OxA-38341	Vildmosen	A4763	9272 ± 45	10,575–10,288	− 26.5	+ 2.0	3.4	This study
OxA-38342	Skellingsted mose	A39565	9261 ± 46	10,567–10,282	− 25.1	+ 3.6	3.5	This study
Ua-46878	Rönneholms mosse*	FP1469	9208 ± 55	10,512–10,244	− 22.9	+ 3.1	3	Larsson et al.^8^*
OxA-38090	Emmersbæk	VHM13821	9120 ± 45	10,405–10,205	− 22.4	+ 2.7	3.2	This study
Ua-464	Rönneholms mosse	FP985	9054 ± 47	10,366–10,157	–	–	–	Larsson et al.^8^
OxA-38230	Lundby mose	487	8592 ± 40	9658–9495	− 23	+ 6.3	3.4	This study
AAR-11949	Fugle Å	N/A	8360 ± 55	9499–9147	–	–	–	This study
OxA-X-2807-18	Tømmerup mose	A45173	8335 ± 50	9476–9143	− 24.3	+ 5.5	3.5	This study
OxA-38347	Knabstrup enge	A45770	8290 ± 43	9428–9136	− 23.4	+ 5.2	3.4	This study
Ua-46480	Rönneholms mosse	FP982	8223 ± 43	9395–9028	–	–	–	Larsson et al.^8^
LuS-11272	Rönneholms mosse	FP1488	8205 ± 45	9295–9023	–	–	–	Larsson et al.^8^
LuS-11268	Rönneholms mosse	FP1466	8195 ± 50	9294–9015	–	–	–	Larsson et al.^8^
Ua-46479	Rönneholms mosse	FP923	8191 ± 46	9280–9020	–	–	–	Larsson et al.^8^
LuS-11243	Rönneholms mosse	FP1589	8185 ± 45	9272–9019	–	–	–	Larsson et al.^8^
AAR-11127	Ulkestrup Lyng	KAM-18325	8095 ± 65	9256–8774	–	–	–	This study
Ua-46482	Rönneholms mosse	FP1198	8145 ± 48	9255–9000	–	–	–	Larsson et al.^8^
AAR-11630	Ulkestrup Øst IV	A47608	8124 ± 44	9243–8991	–	–	–	This study
OxA-38231	Lundby mose	490	8105 ± 40	9243–8815	− 24	+ 5.5	3.2	This study
OxA-38346	Kongsted mose	A40894	8101 ± 42	9243–8795	− 22.4	+ 4.4	3.5	This study
LuS-11269	Rönneholms mosse	FP1470	8065 ± 45	9121–8775	–	–	–	Larsson et al.^8^
OxA-38345	Brokøb	A42422	8021 ± 44	9020–8724	− 21.5	+ 4.0	3.4	This study
OxA-38344	Løjesmølle	A5126	7999 ± 43	9009–8663	− 23.6	+ 3.0	3.4	This study
AAR-30762	Brokøb	A30762	7987 ± 45	9005–8656	− 22.6	+ 3.6	3.3	This study
Ua-46485	Rönneholms mosse	FP1312	7966 ± 73	9008–8610	–			Larsson et al.^8^
OxA-38315	Skamstrup	A44121	7975 ± 39	8997–8655	− 22.7	+ 4.4	3.5	This study
AAR-6867.2	Brokøb B	MC I 081183	7940 ± 65	8993–8609	–	–	–	Fischer^15^
AAR-6867.1	Brokøb B	MC I 081183	7890 ± 65	8984–8562	–	–	–	Fischer^15^
LuS-11270	Rönneholms mosse	FP1483	7925 ± 45	8980–8609	–	–	–	Larsson et al.^8^
Ua-46874	Rönneholms mosse	FP1204	7847 ± 53	8973–8483	− 23.4	+ 6,0	3	Larsson et al.^8^*
Ua-46877	Slabälta	Sla1	7890 ± 65	8969–8562	− 23	+ 5.4	3	Larsson et al.^8^*
LuS-11271	Rönneholms mosse	FP1487	7835 ± 45	8850–8479	–	–	–	Larsson et al.^8^
Ua-46876	Rönneholms mosse	FP1247	7820 ± 50	8765–8455	− 23.6	+ 5.0	3	Larsson et al.^8^*
OxA-X-2807-20	Verup mose	A44111	7790 ± 45	8647–8435	− 23.6	+ 7.2	3.4	This study
Ua-46875	Rönneholms mosse	FP1220	7748 ± 49	8602–8421	− 22.9	+ 5.4	3.1	Larsson et al.^8^*
OxA-X-3004-15	Assentorp mose	A42424	7738 ± 54	8600–8415	− 26.2	+ 3.8	3.9	This study
Ua-46478	Rönneholms mosse	FP762	7745 ± 42	8594–8430	–	-	–	Larsson et al.^8^
Ua-46873	Rönneholms mosse	FP746	7720 ± 54	8592–8413	–	–	–	Larsson et al.^8^

^14^C ages calibrated to cal. years BP in OxCal v.4.3 using IntCal3 calibration curve^16,82^. Asterisk (*) after the site name denotes a second measurement performed on the same artefact. An asterisk after reference denotes additional measurements undertaken in this study (i.e. δ^13^C, δ^15^N and C:N ratios) conducted at the Uppsala Ångström Laboratory on material previously dated by AMS.

Published and unpublished radiocarbon dates from the faunal remains of Eurasian elk (*n* = 73), red deer (*n* = 33), and bovines (aurochs + bison *Bison bonasus*, *n* = 73) reveal reduced frequencies in the faunal remains of these species around c. 10,000 cal. BP in Denmark (see Supplementary Figure 24; Supplementary Information, Section 2; Supplementary Dataset 2). However, no equivalent decline in the dates from Southern Sweden has been observed. If this decline in radiocarbon-dated remains from Eastern Denmark is representative of a decline in the animal populations this would have implications for the availability of raw material for the manufacture of bone points. The absence of bone points also corresponds to a partial absence of dated habitation sites in Eastern Denmark (not including the island of Bornholm) and Jutland (Fig. 2). However, it is during this same gap in the Danish record, that most of the dated habitation sites on the west coast of Sweden occur, as well as on the island of Bornholm (see Supplementary Figures 23 and 24; Supplementary Information, section 2; Supplementary Dataset 3).

### Protein analysis

**Figure 3:**
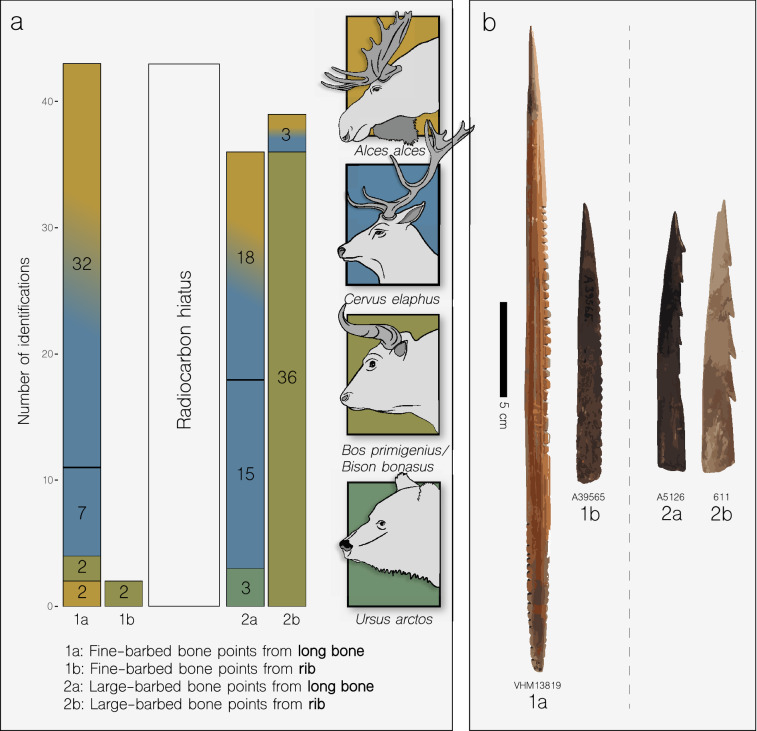
Species identifications of bone points. (**a**) Histogram of 120 summed protein mass spectrometry identifications separated by radiocarbon hiatus (see Supplementary Dataset 4). Each column refers to barbed bone points made of respectively long bone and rib. Colours refer to stylized animal portraits. Gradient colours (blue vs. orange) indicate either red deer or elk, (**b**) selected bone points of each class.

One-hundred and twenty barbed bone points were analyzed by ZooMS^17^ (see Supplementary Information, section 3, Supplementary Dataset 4). They turned out to derive from three groups of mammals: 74 from cervids, 43 from bovines and three from brown bear (*Ursus arctos*) (Fig. 3).

Similarly to aurochs and bison^18^, red deer and elk could not be distinguished using previously published markers, e.g., from Welker et al.^17^. However, through mining of published whole-genome data, we were able to construct more complete sequences for the species revealing five single amino acid polymorphisms (SAPs) between red deer and elk resulting in five potential tryptic peptides (biomarkers) (Supplementary Table 1; Supplementary Information, section 4). In three of these biomarkers, one or both sequences also matched (100%) with environmental bacterial sequences as identified using BLASTp and were discarded (Supplementary Information, section 4). LC–MS/MS analyses of four reference samples (two red deer and two elks) confirmed that the two remaining biomarkers can be used to discriminate between the two species (Supplementary Information, section 4). In the MALDI spectra, the red deer peptide m/z 2216 (GETGPAGR**P**GEVGPPGPPGPAGEK, peptide COL1A1T66/67, position 910-934) results from a missed cleavage because the A → P substitution reduces the efficiency of tryptic cleavage at the adjacent arginine^19^. As m/z 2216 is also present in aurochs we can only use this marker to discriminate red deer once aurochs has been discounted. Unfortunately, masses corresponding to the equivalent sequence in elk were not detected in any of the MALDI spectra, so we can not confirm that samples lacking the m/z 2216 are elk using ZooMS. Of the 74 bone points previously identified as red deer/elk, 22 were reassigned to red deer. Two Preboreal bone points analyzed by LC–MS/MS (VHM13821 and A37811) had diagnostic peptides for elk, while two from the Boreal period (A40894 and A42422) were identified as red deer, confirming the earlier MALDI-MS based identification (Supplementary Table 2).

## Discussion

Radiocarbon dates from Denmark and Scania (southern Sweden) revealed a hiatus starting at c. 10,300 cal BP, lasting nearly 600 years that separates the Maglemose period into two complexes represented by bone points of a markedly different form. Protein analysis revealed that the fine-barbed points (i.e. Preboreal and Early Boreal) were also materially different from the larger-barbed bone points (i.e. Late Boreal).

Fine-barbed points of the Early Maglemose Complex (*n* = 45) were predominantly manufactured from cervid long bones (see Fig. 3) despite evidence for the presence of other large ungulates—aurochs, bison, reindeer (*Rangifer tarandus*), and wild horse (*Equus ferus*)—in Southern Scandinavia at this time^20–22^. This overrepresentation of cervids may be a reflection of the species abundance at the time but more likely represent a deliberate choice by Early Maglemosian hunter-gatherers. Seven of the 45 were identified as red deer based on our novel biomarker. One of the seven was also radiocarbon dated (Øresø Mølle, A52426, 9481 ± 47) and represents the earliest occurrence of red deer in Denmark.

The bone point morphology of the Late Maglemose Complex changes to a larger and more varied barb design. The number of bone points made of bovine ribs increases substantially (from 6 to 93%) in this large-barbed (*n* = 75) assemblage (Fig. 3) and those of brown bear appears for the first time. All confirmed bison remains from Denmark and Southern Sweden have been radiocarbon dated to no later than the Preboreal^20,23^. This seemingly leaves aurochs as the sole persisting bovine species and assumed source of bone points produced after the Preboreal. The bone points made from the aurochs ribs represent the very last remnants of this species before its disappearance on the Danish islands c. 7000 cal BP^24,25^. The selection of aurochs rather than cervid ribs probably reflects its preferable mechanical properties (Supplementary Information 5). It could, therefore, be argued that the selection of the raw materials by Maglemosian hunter-gatherers was based upon practical, rather than for example spiritual, considerations^26^.

The majority of the large-barbed bone points date to the Maglemose culture, while seven dates extend into the beginning of the Kongemose culture (c. 8500–7400 cal BP), after which simple bone points without barbs seem to have been preferred (at least in Central Scania)^27,28^. The transition from large-barbed bone points to simple bone points seems to be synchronous with the arrival of trapezoid lithic armaments^29–31^. This change in material culture may reflect a change of economy and seasonal rounds of the local population: coastal and inland groups merging within Southern Scandinavia as sea levels rose; seasonal spearfishing in the lakes losing its importance by communities that relocated their demographic centres to the coasts during the Kongemose^32^. However, the transition to simple bone points and Blak type trapezoid armaments might also represent the arrival of new migrants, whose new technologies ended the microlithic tradition completely.

Interestingly, the hiatus of dated barbed bone points is also evident in the radiocarbon dates from the *classic* Maglemosian habitation sites in Eastern Denmark, in effect dividing them into two periods of occupation (see Fig. 2 and Supplementary Figure 23, Supplementary Information, section 2; Supplementary Dataset 2). Further, a similar gap in radiocarbon dates of faunal remains further indicates a decline in population size in Eastern Denmark, but interestingly not in Scania (apart from Rönneholms mosse) (see Supplementary Figure 24, Supplementary Information, section 2; and Supplementary Dataset 3). The δ^13^C values obtained from the bone points indicate vegetational change over time from an open environment to a more closed setting (see Supplementary Information, sections 6 and 8). This is unlikely to represent a behavioural change in the animals, but instead probably reflects a landscape characterized by denser forests.

The gap in radiocarbon dates during the Early Holocene might be interpreted as evidence for a decline in human habitation in Eastern Denmark. There is only weak evidence for human presence during the ‘bone point hiatus’. The ^14^C dates from the two settlement sites of Draved and Klosterlund in Jutland fall before and partly during the hiatus^33,34^. However, the radiocarbon dates from these sites are conventional and from the very early days of radiocarbon dating when samples were not processed to remove secondary humic acids; a factor that can often result in misleadingly young dates^35^. Five AMS dates from uniserial bone harpoon points date to the hiatus in Southern Scandinavia. These are from Tunebjerg Øst (9050 ± 40 ^14^C BP) and Trunderup Mose (8845 ± 60 ^14^C BP), both sites on Funen^36^, from Rönneholms mosse in Scania (8610 ± 90 ^14^C BP)^37^ and from Vallensgård Mose on the present-day island of Bornholm in the Baltic Sea (9250 ± 60 ^14^C BP, 8875 ± 65 ^14^C BP)^38^, which would have been connected to Continental Europe at this time.

When large-barbed bone points appear following the hiatus (at c. 9650 cal BP), they show markedly different morphological traits, from their predecessors. They appear once the former major lakes fill and deepen again. Most of the smaller ponds would probably at this time, have grown into fens. Until recently, it was believed that the new bone point morphology appeared before the first indications of the pressure blade lithic industry in Denmark at c. 9000 cal BP^39^. This industry is characterized by small regular blades, created by applying pressure rather than direct percussion. Blades of this type were utilized as cutting-edge inserts in slotted bone points. However, based on a previously unpublished radiocarbon date obtained from a slotted bone implement (Clarks Type B1 or B2) from Fugle Å, near Ulkestrup Lyng in Store Åmose (AAR-11949, 8360 ± 55 ^14^C BP) (see Fig. 2 and Supplementary Figure 29); pressure flaking is now contemporary with the emergence of large-barbed bone points.

The reason for this gap in the radiocarbon record which seemingly separates two cultural traditions is currently unknown. Several factors could have caused a small and confined human population to almost disappear from the archaeological record; i.e. epidemics, warfare, changes in subsistence strategy, climate, and migration. However, most of these factors are difficult to tie to the disappearance of bone points in the Southern Scandinavian lakes. Several local studies have revealed climatic fluctuations in the Northern Hemisphere during the Early Holocene including lower temperatures and increased precipitation^40–47^. To our knowledge, no evidence of a similar cool interval or increase in precipitation between c. 10,300 to 10,000 cal BP has been reported for Southern Scandinavia. This may be due to a lack of high-resolution studies of sediments from this period coupled with a sampling bias, further complicated by contemporaneous erosion events^48–50^.

High-resolution studies coupled with direct radiocarbon dating of sediment cores conducted in Southern Sweden, however, do not show an increase in precipitation in lake levels^51, 52^ at that time. Rather, they indicate dry climate and the lowest water levels of the entire Holocene during the early Boreal^51–53^. The northern expanse of hazel (*Corylus avellana*) at this time^1^ is also believed to have been facilitated by the markedly lowered water levels in the lakes and fens^20^. Pollen analyses of the Åmose basin on Zealand and Rönneholms mosse in Scania also show several marked water level changes that occurred during the Early Holocene^48,49^, although these are not correlated by direct radiocarbon dating of the sediments. During the transition from the Preboreal to the Boreal, a brief, but significant lowering of the water levels took place, which was shortly followed by renewed transgression^54^. In Åmosen, this regression led to erosion of the littoral zone and the redeposition of sediments, basically removing most Preboreal riparian sites and sediments associated with this period^50^. As in Scania, the lowering of the water level was also followed by a transgression^48^. The reason for these water level fluctuations is difficult to determine. One factor during the Early Preboreal could be the melting of stagnant ice, whereby glacier ice melted in situ. Temperature oscillations may also account for some of these water-level changes, with dryer climatic conditions resulting in increased evaporation^55^. “The general notion of a particularly dry climate during this period also seems to be reflected in sediment cores from Eastern Denmark, Southern Sweden and Western Germany, which all contain increased levels of charcoal, argued to have been caused by wildfires^48, 56,57^. Similarly, Jørgensen observed pollen from the shrub Ephedra sp. in Åmosen at the interphase between the Preboreal and the Boreal periods. Ephedra is known to thrive in arid environments, indicating that Åmosen might have been arid at this time period, although he also argues that it could have been transported by the wind^48^. If the climate did indeed force hunter-gatherers to relocate and abandon their old activity areas, these actions might be reflected in radiocarbon dates elsewhere in the form of potential bust and boom cycles and a rapid diffusion of technologies.

Interruption in ^14^C dates or drastic changes in the archaeology elsewhere in Europe is also centred around 10,000 cal. BP. In Eastern Fennoscandia, a c. 200-year long gap seems to occur shortly after 10,300 cal. BP, which has been interpreted as a decline in the local population as a result of an abrupt climate event^58^. Radiocarbon dates of human skeletal remains in Central Germany also reveal a similar gap, indicating either an absence of humans in the period or potential preservation or sample bias^59^. Interestingly, in North-western Europe this period marks a radical change in microlithic technology contemporaneous with increased droughts and severe wildfires in the region^60^. In Southern Norway, a similar diffusion event occurred, where pressure blade technology suddenly appeared^61^. These two studies testifiy to the notion that the environment must have affected the Early Mesolithic populations of Northern Europe.

The falling water levels^62^ would almost certainly have impacted the fishing and hunting opportunities and may have caused humans to move away in search of viable fishing grounds. This may account for the observed decrease of butchered animal remains discarded in environments conducive for preservation. However, ecological stress caused by increasing temperatures, coupled with potential wildfires in drying mixed coniferous forest, may also have triggered the movement of humans and animals alike.

The presence and continued use of fine-barbed bone points in Northern Germany during the hiatus^12,63^ suggest that fishing practices were not as disrupted further to the south. While dates from the Swedish west coast; Huseby Klev and Balltorp^64–66^ and a further 10 AMS dates from charred remains from the habitation site of Ålyst on Bornholm (Supplementary Figure 24; Supplementary Dataset 2), also suggest the arrival of new groups there. Subsistence at the Swedish sites focused primarily on marine mammals, fish, and birds^67^. Unfortunately, the coastlines of the Early Holocene in Denmark became submerged during the Atlantic period due to rising sea levels (see Supplementary Information 7), meaning that Early Mesolithic coastal sites are rarely located and excavated in this region^68,69^. Consequently, it is not possible to study and compare coastal settlements which could corroborate a shift in economic strategy and subsistence on a regional scale during the Early Mesolithic in this area. However, human exploitation of marine resources along coastal environments in Southern Scandinavia can be inferred from stable isotope data obtained from human remains. Individuals from Køge Sønakke off the coast of Eastern Zealand in Denmark^70^, Österöd^71^ and Huseby Klev^65,72^ from the South-western coast of Sweden are contemporaneous with the identified hiatus. These individuals yielded collagen δ^13^C and δ^15^N values consistent with partially or fully marine diet (Supplementary Dataset 2). Indeed the presence of harpoons during the Mesolithic has strongly been tied to the hunting of marine mammals, although the fact that they ended up in the potentially dried up lakes may indicate a more varied use^36^.

In summary, radiocarbon dates show a hiatus, spanning c. 600 years, separating the two classified types of bone points at c. 10,300 cal BP. The hiatus of dated barbed bone points are also evident in the radiocarbon dates from the habitation sites in Eastern Denmark, in effect dividing the Maglemose into two complexes, displaying distinctly different technologies; fine-barbed bone points and percussion knapping in the Early Maglemose Complex, and large-barbed bone points and pressure flaking in the Late Maglemose Complex. These two complexes represent two radically different material cultures and technological traditions which challenges the notion of a period in relative stasis. We are confident that this surprising gap in the South Scandinavian material will find more parallels elsewhere in Europe.

The cause of this radiocarbon gap is currently unknown, but we hypothesize that climate change caused water-levels to drop and thereby forced the Mesolithic people to relocate and adapt their subsistence strategies, potentially along the now submerged coasts. Another possibility coupled to low water levels could be the increase of wildfires due to an exceedingly dry climate, which may also have forced hunter-gatherers to relocate.

The introduction of pressure blade technology is now inferred to be synchronous with the emergence of large-barbed bone points after the hiatus and may indicate the transmission of influences from Sweden. Species identification by ZooMS and LC–MS/MS indicates that a conscious selection was carried out in regards to the specific species (as well as the preferred skeletal elements, i.e. rib or long bone) for manufacture.

The pressure blade industry which characterises the Late Maglemose Complex is thought to have originated at c. 20,000 cal BP in the area of present-day Siberia/Northern China from where it spread westward to Western Russia^73^, and subsequently into Northern Fennoscandia^39,74,75^. Results from archaeological studies on the diffusion of pressure blade technology correspond well with genetic studies from Norway and Sweden. These have shown that the individuals involved in the spread of pressure blade technology, were genetically admixed between Western and Eastern Hunter-Gatherers^76^, and thus indicate migration routes westwards from Russia into the Scandinavian peninsula while the route into Denmark remains inconclusive^75^ as it could also have spread through Poland and Germany. Most recently, re-analyses of lithic remains attributed to the oldest sequence at Huseby Klev (deep pit) revealed the presence of pressure blade technologies, which are securely dated to the hiatus and the subsequent decades (10,040–9610 cal BP). Moreover, the same study also reported on aDNA extracted from chewed birch pitch revealing that the individuals were of genetically admixed ancestry^77^. This novel lithic technology coupled with a changed bone point morphology and the pattern of radiocarbon dates from Western Sweden and Central Scandinavia^78^ favours the spread of this technology through Scania and into present-day Denmark.

It is hoped that future high-resolution sedimentary studies are conducted to elucidate if and in what ways the local climate affected the people inhabiting this area. Similarly, direct radiocarbon dates of material from classic Maglemosian sites, as well as genetic studies, will hopefully help to illuminate whether the Early and Late Maglemose Complex are also genetically as well as technologically distinct.

## Methods

### Materials

The bone points analyzed (*n* = 127) all derive from Southern Scandinavia (Denmark and Southern Sweden) (see Supplementary Information 1). The bone points were, based on barb morphology and skeletal element used in their manufacture (i.e. long bone versus rib), and to some extent species, divided into two groups.

A subset of the bone point samples was further submitted for AMS radiocarbon (^14^C) dating (*n* = 23), protein analysis to determine the species of each artefact (*n* = 120), and carbon (δ^13^C) and nitrogen (δ^15^N) isotopic analyses (*n* = 19). This has resulted in two distinctive groups extending from the Preboreal into the Atlantic Period, corresponding to the Maglemose and slightly beyond, into the subsequent Kongemose culture.

### Radiocarbon dating

We submitted bone powder or bone fragments (mean weight 100 mg) of 23 bone points from Denmark for AMS dating at the Oxford Radiocarbon Accelerator Unit based on their typological grouping. Collagen was extracted from bone powder using ORAU pretreatment codes AF (samples A44111, 436, 487 and 534) and AG (all other samples^79^. The extracted collagen was combusted, graphitised and dated according to Dee and Ramsey^80,81^ and Ramsey et al.^80,81^. Of the 23, 21 were successfully dated and were subsequently merged with 24 published AMS dates^8,14,15^ as well as seven unpublished dates from Zealand (see Supplementary Dataset 1). Two artefacts (FP1469: 9375 ± 45 ^14^C BP, 9208 ± 55 ^14^C BP, and Brokøb B: 7940 ± 65 ^14^C BP, 7890 ± 65 ^14^C BP) were dated twice. Isotopic analysis of the dated collagen samples was conducted offline using combustion IRMS at the Oxford Radiocarbon Accelerator Unit, as were a further five samples dated at The Tandem Laboratory at Uppsala University for which sufficient collagen remained (Supplementary Dataset 1).

The total of 52 AMS dates was then calibrated to cal. years BP in OxCal v.4.3 using IntCal3 calibration curve^16,82^. We also compiled 68 published and unpublished radiocarbon dates performed on charcoal and bone associated with habitation sites, including human remains not directly associated with habitation (Supplementary Information 2), and 118 published and unpublished radiocarbon dates from faunal remains (elk *n* = 33, red deer *n* = 12, auroch and bison *n* = 73) spanning the Maglemose to infer presence or absence (Supplementary Information 2). We applied Bayesian phase modelling on the dates from habitations, assuming the coeval age of habitation events using OxCal v.4.3^82^ (see Supplementary Dataset 3 and Supplementary Figure 25).

### Proteomics

We performed ZooMS on 120 bone points from Denmark and Scania in Southern Sweden using protocols from^83,84^ (see Supplementary Information 3). Mass spectrometry was conducted on a Bruker MALDI-TOF–MS/MS instrument in reflector mode to acquire spectra from 800 to 3500 m/z. Taxonomic identification was completed using published markers^17^. As red deer (*Cervus elaphus*) and European Elk (*Alces alces*) cannot be distinguished with published markers^85^, we compared their collagen (COL1ɑ1 and COL1ɑ2) sequences and identified five single amino acid polymorphisms (SAPs). We then analyzed these SAPs to see if they provided unique tryptic markers for ZooMS analysis (see Supplementary Information 4).

To confirm our candidate ZooMS marker capable of discriminating between red deer and elk, four reference samples (two from each of these species) were digested with Trypsin, Elastase and Chymostrypsin and sequenced using LC–MS/MS (see Supplementary Information 3). In addition, we sequenced the tryptic peptides from four bone points, two from the Preboreal and two from the Boreal (see Supplementary Information 3).

## Supplementary information

Supplementary Dataset 1.

Supplementary Dataset 2.

Supplementary Dataset 3.

Supplementary Dataset 4.

Supplementary Dataset 5.

Supplementary Information.

## Data Availability

The mass spectrometry data for both ZooMS and LC-MS/MS have been deposited to the ProteomeXchange Consortium via the PRIDE^86^ partner repository with the dataset identifier PXD018050.
