# Supplementary material for: An integrated analysis of Maglemose bone points reframes the Early Mesolithic of Southern Scandinavia
Source: Sci Rep. 2020 Oct 14;10:17244. doi: 10.1038/s41598-020-74258-8 (PMC7560828; doi:10.1038/s41598-020-74258-8)
Supplement: Supplementary file 6 — Supplementary Information. [file 41598_2020_74258_MOESM6_ESM.pdf]

Supplementary Information for

An integrated analysis of Maglemose bone points reframes the Early Mesolithic of Southern Scandinavia

Theis Zetner Trolle Jensen[1,2*], Arne Sjöström[3], Anders Fischer[4], Erika Rosengren[3,5], Liam Thomas Lanigan[2], Ole Bennike[6], Kristine Korzow Richter[7], Kurt Joseph Gron[8], Meaghan Mackie[2,9] Morten Fischer Mortensen[10], Lasse Sørensen[11], David Chivall[12], Katrine Højholt Iversen[9,13], Alberto John Taurozzi[2], Jesper Olsen[14], Hannes Schroeder[2], Nicky Milner[15], Mikkel Sørensen[16], Matthew James Collins[2,17*]

[1]BioArCh, Department of Archaeology, Environment Building, Wentworth Way, University of York, YO10 5NG, York, UK.
[2]Section for Evolutionary Genomics, GLOBE Institute, Faculty of Health and Medical Science, University of Copenhagen, Øster Farimagsgade 5, 1353 Copenhagen, Denmark.
[3]Department of Archaeology and Ancient History, Lund University, Helgonavägen 3, Box 192, 221 00 Lund, Sweden.
[4]Sealand Archaeology, Gl. Roesnaesvej 27, 4400 Kalundborg, Denmark.
[5]Historical Museum, Lund University, Krafts Torg 1, 223 50 Lund, Sweden
[6]Geological Survey of Denmark and Greenland (GEUS), Øster Voldgade 10, Copenhagen K, Denmark.
[7]Department of Archaeology, Max Planck Institute for the Science of Human History, Kahlaische Strasse 10, 07745 Jena, Germany.
[8]Department of Archaeology, Durham University, South Road, DH1 3LE, Durham, UK.
[9]Novo Nordisk Foundation Center for Protein Research, University of Copenhagen, Blegdamsvej 3b, 2200 Copenhagen N, Denmark.
[10]The Danish National Museum, Department of Environmental Archaeology and Materials Science. I.C.Modewegsvej, 2800 Lyngby, Denmark.
[11]The Danish National Museum, Ancient Cultures of Denmark and the Mediterranean, Ny Vestergade 10, Prinsens Palæ, 1471 København K, Denmark
[12]Oxford Radiocarbon Accelerator Unit (ORAU), School of Archaeology, University of Oxford, 1 South Parks Road, Oxford OX1 3TG, UK
[13]Department of Health Technology, Technical University of Denmark, Kemitorvet, 2800 Kgs. Lyngby, Denmark.
[14]Aarhus AMS Centre (AARAMS), Department of Physics and Astronomy, Aarhus University, Ny Munkegade 120, 8000 Aarhus C, Denmark.
[15]Department of Archaeology, University of York, King's Manor, York, YO1 7EP, UK.
[16]The Saxo Institute, Department of Archaeology, University of Copenhagen, Karen Blixens plads 8, 2300 Copenhagen S, Denmark.
[17]McDonald Institute for Archaeological Research, University of Cambridge, West Tower, Downing St, CB2 3ER, Cambridge, UK.

[*]To whom correspondence may be addressed. Email: tztjensen@sund.ku.dk; matthew.collins@palaeome.org

Supplementary Note 1. Bone points 3

Supplementary Note 2. Radiocarbon dates 6

Supplementary Note 3. Collagen analysis by Mass Spectrometry 8

Supplementary Note 4. Database Creation and Hypothetical Marker Determination 11

Supplementary Note 5. Bone Density - ribs vs. long bones 13

Supplementary Note 6. Isotopic analysis of collagen from bone points 13

Supplementary Note 7. Water Level Fluctuations in Danish/German/Swedish Straits 14

Supplementary Discussion 8. Potential Causes for Absence of Bone Points 16

Supplementary Figures 18

Supplementary Tables 48

References 53

Supplementary Note 1. Bone points

Theis Zetner Trolle Jensen, Arne Sjöström, Morten Fischer Mortensen & Anders Fischer

In the present study, the neutral term 'barbed bone point' is used as a descriptor of the artefact under consideration, as opposed to functional laden descriptors, such as; leister-points or prongs. While the latter idioms are not inaccurate *per se,* they denote that the bone points are part of a composite system in which more than one barbed bone point is fastened to a shaft and therefore imply their functional usage [1]. Their function was undoubtedly fishing gear, but evidence for composite leisters is only circumstantial (e.g. the proposed leister sets from Fulge Å and Mosegården III in Åmosen [1])

The majority of Maglemosian bone points from Denmark are stray finds, collected in connection with peat cutting during the World Wars and field surveys in the previously harvested bogs. Only a small proportion derives from targeted excavations. Therefore, provenance is rarely exact and site descriptions can often be vague or in most cases entirely absent. A majority of these chance finds derive from large bog complexes from Zealand. Lille Åmose (Little Åmose), and Store Åmose (Greater Åmose), complexes, will be briefly introduced, as well as, the bogs Rönneholms and Ageröds mosse in Scania. Photos of the bone points and their approximate provenance are visualised in Fig. S1-22 (Photos by Theis Zetner Trolle Jensen & Arne Sjöström).

Åmosen, Zealand, Denmark

The Åmose (translated: river bog) covering 36 km² is the largest peat bog complex on the Danish islands and the largest filled in lake system in the country [1]. It encompasses two major parts: Lille Åmose and Store Åmose, and the two probably comprise the richest area in NW Europe in terms of Mesolithic and Neolithic inland finds [2]. These archaeological riches were, in general, of an extraordinarily fine preservation quality until c. 1960 AD, when severe drainage events were initiated and over the following years led to the peat decomposing over major areas [2]. During the 1940s and 1950s peat cutting was intensified due to fuel shortage, and a magnitude of well-preserved settlements was uncovered. Barbed bone points have been found nearly all over the bog, and reflect the intense fishing activity carried out during the Mesolithic [3].

The Åmose basins have a long and complex developmental history, which have been the focus of dozens of geological and palaeoecological investigations over the last 100 years (eg. [4,5]. One prominent feature is the dramatic water level changes that occurred during the Early Holocene. The first big change happened in the Early Preboreal when the water level in Greater Åmose decreased as much as four metres, and the only preserved sediments from this time are confined to the deepest areas of the basin [6]. Although a subsequent water level rise occurred, the lake surface remained relatively low. During the transition from the Preboreal to the Boreal, a brief lowering of the water levels took place yet again. This regression led to erosion of the littoral zone and redeposition of sediments elsewhere.

A later regression happened around the transition from the Boreal to the Early Atlantic period, leading to severe erosion of the littoral Boreal sediments. This caused a hiatus of 200-300 years in the sedimentary record during the Late Boreal; based on samples from several locations in the bog. The reasons for these water level fluctuations are difficult to determine. Important factors could be the melting of local stagnant ice and temperature oscillations. In addition, beavers can influence water levels with their dams, resulting in significant changes, not just fluctuations during construction, but also during periodic dam bursts.

As a result of the lowering of the lake surface during the Holocene, it is commonplace to find lakeshore settlements of the Kongemose, Ertebølle and Funnel Beaker periods located on top of Maglemosian 'fishing grounds' (i.e. lake bottom sediments rich in Early Mesolithic bone points).

Rönneholms mosse, Scania, Sweden

The peat bogs Rönneholms mosse and the Ageröds mosse together form a 12 km^2 large wetland area, separated by the Rönne river, northwest of the lake Ringsjön. Before the peat bogs formed in the Atlantic and Subatlantic biozones, the area constituted a shallow lake that was part of a larger lake Ringsjön (c. 50 km^2).

The first finds in the bog area were found at the beginning of the 20th century in connection to peat extraction and trenching. Several Mesolithic sites, mainly dated to the Maglemose and Kongemose cultures, were excavated in the 1940s at the former shorelines of the ancient lake and on peat islands in Ageröds mosse [7]. Excavations at several of the sites were continued in the 1970s and the 1980s [8,9]. A few barbed bone points were found at these sites, but since they are not radiocarbon dated, they have not been included in the study. Only two points from Ageröds mosse have been dated, found at the recently excavated bog site Slabälta [10]. Since the peat extraction in Ageröd was conducted in the first half of the 20th century, in a relatively large-scale by digging deep trenches, there is no data regarding whether or not finds like fishing tools were found outside the settlement areas in this part of the bog.

In Rönneholms mosse, peat has been commercially extracted by horizontal and mechanical stripping, thus exposing settlements and other remains within a large area on an annual basis. It has therefore been possible to study the geological development and the human activity in this part of the bog area in more detail. Archaeological remains have been found over the course of 26 years of excavations and field recognisance conducted by Arne Sjöström of Lund University.

In the southern part of the ancient lake, in the Rönneholm basin, a layer of lime gyttja was deposited during the Preboreal and Boreal biozones. There are very few signs of fishing activities in this part of the ancient lake during this period. Only a few barbed bone points and pine torches, dated to the Late Preboreal, have been found in the lime gyttja, and there are no signs of net fishing in the layer. However, it should be noted that the peat extraction field in Rönneholms mosse is located a few hundred meters from the presumed Preboreal shoreline and that other conditions may occur closer to land.

When the algae gyttja began to deposit on top of the lime gyttja, in the Late Boreal and Early Atlantic, there was a dramatic increase of human activity in the Rönneholm basin. Thousands of single finds and over 100 small campsites have been found scattered all over the basin in this layer, mostly dated to the Early Atlantic. Thousands of net sinkers made of stone show that net fishing was common. Also, numerous bone points were found in the algae gyttja during the extensive surveys at the Rönneholm mosse [11].

The radiocarbon-dated barbed bone points from the bog area indicate that the use of this tool type in central Scania ended in the transition from the Maglemose to Kongemose culture. Finds of other tool types made of bone and numerous sites in the bog, dated to the Kongemose culture, show that the disappearance of barbed bone points was not caused by poor preservation conditions. In the Kongemose, spearfishing was most likely performed with leisters with wooden prongs and simple bone points without barbs. The Scanian study was based on a rather uniform assemblage of 35 barbed bone points from the Rönneholm and Ageröd peat bogs.

The geological development of the Rönneholm-Ageröd basin has been studied by Tage Nilsson [12–15]. The Rönneholm basin has only been partially studied by Nilsson in 1935, but since both the basins formed a single lake during the early postglacial time the detailed studies from the Ageröd basin can partially be applied to the Rönneholm basin.

Nilsson presented a hypothetical curve of the water level fluctuations, without absolute levels. He found that the water level in the lake was relatively high at the beginning of the Preboreal, and that it dropped during this period and the Early Boreal to form a low stand in the middle of the Early Boreal. In the transition to the Late Boreal, a water level rise occurred, before the level dropped to a post-glacial minimum level during the end of the Late Boreal. In the transition to the Early Atlantic, the water level rose to a high level before it dropped again at the end of the period to form a longer low water stand during the Late Atlantic [13,15].

It is difficult to determine the time length, the levels, and the cause of the low water periods that Nilsson presented. The archaeological excavations in Rönneholms Mosse have shown that there have been several short periods with extreme low stands in the Rönneholm basin during the Early Atlantic, since small campsites from the same period have been found in the gyttja layers all over the basin. These short low water periods, when the basin was more or less dried out, have not been confirmed in the geological record. In spite of these extreme low water stands, there have been activities with spearfishing in the lake. This is known by numerous single finds of bone points situated at the same levels as the sites. This contradiction can probably be explained by extreme yearly changes in water level in the shallow lake during the Early Atlantic. A similar settlement pattern; with sites situated offshore in the dried out lake basin, has not been seen for the low water periods during the Boreal biozones.

Supplementary Note 2. Radiocarbon dates

Bone points

Theis Zetner Trolle Jensen, Jesper Olsen, Anders Fischer

We submitted 23 bone points for radiocarbon dating. Two failed due to low collagen yields. Twenty-one returned dates spanning the Preboreal, Boreal, and the beginning of the Atlantic biozones, while two turned out to have C/N values outside the accepted range and should be treated with caution (C/N values >3.6, Supplementary Dataset 1). We merged our successfully dated bone point results with 20 dates from Sweden published by Larsson *et al.* [11], the five published by Fischer [16,17], and seven previously unpublished dates from northwest Zealand (1; Brokøb, 1; Sønderød, 2; Mørke Enge, 1; Ulkestrup Øst IV, 1; Ulkestrup Lyng), plus a slotted bone point from Fugle Å (Supplementary Dataset 1).

Habitation debris and stray found Preboreal, Boreal and Early Atlantic fauna

We collected radiocarbon dates from Maglemosian habitations as well as from faunal remains of that period. This was done to infer the activity levels of the archaeology and faunal richness during the 10,300 cal BP bone point hiatus. However, caution should be exercised when using these dates as proxies for presence or absence or using them at all. Firstly, most of the published dates were performed using conventional radiocarbon methodology several decades ago, when collagen preservation quality etc. was not treated as critically as nowadays. Secondly, because the majority was performed on charcoal that can reflect large offsets and long age spans due to the "old wood effect", and thirdly, not all present-day purification steps were routinely performed decades ago. For instance removal of humic acids from the collagen fraction was not routinely performed in the pioneering days of the former ^{14}C laboratory in Copenhagen. This procedure only became standard in sample preparation from laboratory number K-2127 onwards (Henrik Tauber pers. comm. to Anders Fischer [2,18]. As a consequence, some of these samples may have produced dates that are misleadingly young due to infusion of humic washed down from stratigraphically younger deposits.

Habitation

We collected published and unpublished (n = 38) radiocarbon dates conducted on material (bone or charcoal) associated with habitation sites in Denmark. We performed a simple Bayesian model for each site creating phase models, assuming that the dates from each of the sites are of coeval age. We also summed the radiocarbon dates in a KDE model to visualise activity. In addition, we included dates (n = 22) from the southwestern coast of Sweden, from Balltorp and Huseby Klev. Lastly, three dates performed on three isolated human remains (two burials and dredged off the coast) not associated with habitation were also included. The human remains were reservoir corrected based on their δ^{13}C values. The marine reservoir age, R, is estimated to 400 ±50 ^{14}C years and the fraction marine, F_m, diet of each individual is calculated using a terrestrial and marine δ^{13}C endpoint value of -21‰ and -10‰ respectively

[19]. The radiocarbon reservoir correction for each individual is then calculated as $^{14}C_{corrected}$ = $^{14}C_{measured}$ - $F_m \times R$. Errors on the $^{14}C_{corrected}$ age are calculated using error propagation. The habitation sites included are from Denmark: Barmose I, Klosterlund, Ålyst, Mullerup, Holmegaard, and Ulkestrup in Denmark. As well as Balltorp and Huseby Klev in Sweden (see Supplementary Dataset 2, and Fig Supplementary Figures 23 and 24).

Fauna

We collected (*n* = 118) radiocarbon dates from the Maglemose period performed on faunal remains of the four main species observed by ZooMS in the bone point assemblage (i.e. elk, red deer, aurochs, and bison) from the Preboreal, Boreal, and Early Atlantic biozones. No radiometric dating has been performed on brown bear from the Maglemose period. We performed a Bayesian phase model for each of the taxonomic groups within each region, we then summed the radiocarbon and performed KDE to indicate activity for all dates from a given region (see Supplementary Dataset 3, and Supplementary Figure 25).

Supplementary Note 3. Collagen analysis by Mass Spectrometry

Theis Zetner Trolle Jensen, Kristine Korzow Richter, Meaghan Mackie & Alberto John Taurozzi

Sampling

The sampling and protein extraction of the bone points was conducted in the palaeoproteomics laboratories at BioArCh, University of York, United Kingdom and Centre for GeoGenetics, University of Copenhagen, Denmark. An average of 20 mg of bone was either cut off or drilled at low rpm to produce bone powder. The blade or drill was cleaned in 5% bleach followed by 80% ethanol between sampling. For the reference samples, two elk samples were obtained from the Zoological Department, University of Copenhagen, and two red deer specimens from morphologically distinguishable bone fragments were taken from ongoing Syltholm excavations, near Rødbyhavn, Denmark.

Collagen Extraction

Two different extraction protocols were tested for the samples from these locations. 120 samples were tested (41 by Extraction 1 and 79 by Extraction 2). This was due to the discovery that one gave greater peptides yields than the other. Extraction 1 provided better quality spectra (higher intensity and better resolution) and allowed a higher throughput of samples. It was therefore used for all subsequent samples. However, both methods allowed for species identification of the samples and the results are combined.

Extraction 1 followed a minimally destructive protocol by van Doorn *et al.* [20]. The samples were incubated in 100µL of 50 mM ammonium bicarbonate solution (NH_4HCO_3) pH 8.0 (AmBic) for 16 hours at ambient temperature. They were then vortexed for 15 seconds and centrifuged at 13,000 rpm for 1 min, the supernatant was discarded. 100µL of AmBic was added to the samples, followed by incubation for one hour at 65°C to gelatinise the collagen.

Extraction 2 followed a modified destructive protocol by Buckley *et al.* [21]. The samples were demineralized in 250µL of 0.6M HCl at 4°C for approximately a week, with the acid changed daily. They were then vortexed for 15 seconds and centrifuged at 13,000 rpm for 1 min. The supernatant was discarded and the samples were rinsed three times with 250µL of AmBic. Finally 100µL of AmBic was added to the samples, followed by incubation for one hour at 65°C to gelatinise the collagen.

Enzymatic Digestion and peptide clean-up

For all samples, 50µL of each extraction was transferred to a separate 1.5mL Eppendorf tube, 1µL of sequence grade Trypsin (0.4 µg/µl) (Promega) was added to each followed by incubation at 37°C for c. 16 hours.

Additionally, the four reference samples *Alces alces* (P220 and P221), *Cervus elaphus* (X4787 and X4997) which were were extracted using the Extraction 1 protocol were also enzymatically digested with three different enzymes to increase the sequence coverage for collagen sequencing. After gelatinization, the reference samples were dried using a vacuum centrifuge and resuspended in 100µL Tris-HCl, pH 8. 50µL was transferred to a new 1.5mL tube and incubated with 1µL of elastase (0.4 µg/µl) at 37°C for c. 16 hours. The remaining 50µL was incubated with 1µL of chymotrypsin (0.4 µg/µl) at 25°C for c. 16 hours.

After digestion, the extractions were centrifuged at 13,000 rpm for 1 min before acidification to <pH 2 using 5% (vol/vol) Trifluoroacetic acid (TFA, Sigma Aldrich). Purification was performed using C18 reverse-phase resin ZipTip® pipette tips (EMD Millipore) according to the manufacturer's instructions. Peptides were eluted in 50µL.

MALDI-TOF-MS

Peptide eluates of the tryptic digested collagen were co-crystallised with α-cyano-4-hydroxycinnamic acid (Sigma Aldrich) matrix solution (50% ACN, 0.1% TFA (vol/vol)) at a ratio of 1:1 (1µL:1µL). Samples were spotted in triplicate with calibration standards onto a 384 spot ground steel MALDI target plate (Bruker). Samples were run on a Bruker Ultraflex III MALDI TOF/TOF mass spectrometer (Centre for Excellence in Proteomics at the University of York, United Kingdom) with a Nd:YAG smart beam laser, with a SNAP averaging algorithm used to obtain monoisotopic masses (C 4.9384, N 1.3577, O 1.4773, S 0.0417, H 7.7583). The MALDI was run in reflector mode over an m/z range of 800–3200. The generated spectra were converted to txt files and analysed using mMass v.5.5.0 [22]. The triplicate raw files were averaged, and then peak picked with a S/N threshold of 4 (raw data uploaded to PRIDE together with the LC-MS/MS data under the identifier PXD018050). The nine published biomarkers [21,23] were used to identify the spectra to taxonomic groups (Supplementary Dataset 4). An additional biomarker was discovered (see Supplementary Information 4) at m/z 2216 which was used to identify red deer. Tryptic collagen peptides identified by MALDI TOF were numbered based upon the scheme used by [21]; thus A1T14 and A2T67 respectively enumerate the 14th and 67th tryptic peptides along the Col 1α1 and Col 1α2 chains of the bovine collagen sequence, positions are also given in the sequence according to the alignment to the bovine collagen reference sequence.

nLC-MS/MS

For the four reference samples, the different digestion elutions were dried using a vacuum centrifuge and resuspended in 50µL 80% ACN, 0.1% formic acid (FA). 10µL of each digestion was combined in a 96 well plate (one well per sample).

For the four bone points (VHM13821, A37811, A40894, A42422), the enzymatically digested and eluted peptides were measured by NanoDrop spectrophotometry (Thermo

Scientific, Wilmington, DE, USA) for protein concentration. The volume required for approximately 1.5 μg of peptide per sample was placed in separate wells in a 96-well plate and topped up to 30 μL using 40% ACN, 0.1% FA.

The 96 well plate, in both cases, was vacuum centrifuged at 45ºC until approximately 3 μL was left in the wells, and the samples were then rehydrated with 10 μL of 0.1% TFA, 5% ACN. The samples were analyzed on anEASY-nLC 1200 (Proxeon, Odense, Denmark) coupled to a Q-Exactive HF (reference) or HF-X (bone points) (Thermo Scientific, Bremen, Germany) at the Novo Nordisk Foundation Center for Protein Research, the University of Copenhagen. The parameters used were as previously published [24] with 5µl of sample injected.

Analysis of nLC-MS/MS data

MaxQuant (v.1.6.2.6a or v.1.6.3.4) [25] was used to search the resulting raw files. The database contained a) elk collagen type I sequences, b) red deer collagen type I sequences (Supplementary Dataset 5), and d) common contaminants (from MaxQuant). Species diagnostic peptides found in bone points and reference material are shown in Supplementary Table 2. In addition, the four bone points were run against the soil bacteria *Klebsiella pneumoniae* proteome (see Section 4). MaxQuant parameters were: digestion mode semi-specific for trypsin, to account for possible additional hydrolytic cleavages occurring during diagenesis; variable modifications - oxidation (M,P), acetyl (N-term), deamidation (N,Q), pyro-Glu (E,Q); minimum score for unmodified and modified peptide searches was 60. The remaining parameters were set to the program defaults. Deamidation was assessed using publicly available code [24].

Supplementary Note 4. Database Creation and Hypothetical Marker Determination

Kristine Korzow Richter, Theis Zetner Trolle Jensen, Meaghan Mackie & Alberto John Taurozzi

Elk and red deer collagen sequences and theoretical markers

The Collagen type I sequences from elk come from previously published sequences [26], with missing amino acids substituted with ones from the same positions based on the *Bos taurus* sequence (COL1A1 P02453 and COL1A2 P02465). The collagen type 1 sequences from red deer comes from the red deer genome. At the time of analysis, the collagen type I sequences were not annotated. Therefore a blastx search of the *Bos taurus* collagen sequences against the entire red deer genome (Bioprojects: PRJNA324173) was used to find the location of the collagen genes (COL1A1 - chromosome 5 MKHE01000005.1:143374497-143390891; COL1A2 - chromosome 18 CM008025.1:14219961-14252107). In order to make sure all of the exons were aligned appropriately, the DNA sequence covering the entire gene was aligned to the corresponding *Bos taurus* genes which have annotated exons available on NCBI using Geneious Prime 2019.03. The introns were removed and the resulting sequence translated and aligned to the *Bos taurus* and elk collagen sequences. The aligned sequences were then used to identify potential SAPs and the corresponding tryptic peptides that distinguished red deer and elk.

The tryptic peptides that contained SAPs were then searched against the non-redundant protein sequences using blastp. Any of the tryptic peptides which matched with 100% identity to a non-collagen sequence were removed from the list of biomarkers.

Possible soil bacteria

In order to confirm that the key peptides which distinguished elk and red deer were not soil contamination, the raw files from four bone points were also searched against the proteome of the soil bacteria *Klebsiella pneumoniae* (NCBI:txid573). This is due to the theoretically *Cervus elaphus* specific peptide GAPGPDGNNGAQGPPGPQGVQGGK (COL1A2T40, position 518-541) occurring in all sequenced bone points with conflicting species-specific markers. When searched with blastp, it matches the collagen-like protein (WP_139109296.1) of *K. pneumoniae*. The search against the proteome of *K. pneumoniae* gave evidence of additional proteins from this species being present, indicating that this peptide *could* be derived from soil contamination and this peptide was, therefore, not used for species identification. In addition, another species-specific peptide (COL1A2T51, position 662-669) GDVGSPGR for *Cervus* and GDIGSPGR for *Alces*, was found after a blastp search to match many different potential soil bacteria and was also not used for species specificity (Supplementary Table 1).

Identification of peptide markers to discriminate Red Deer from Elk.

Collagen tryptic peptide A2T66 (position 829-845, Red deer - **S**GETGASGPPGFAGEK; Elk - **T**GETGASGPPGFAGEK) was present in the LC-MS/MS data, but peaks corresponding to the masses of these peptides were not visible in any of the MALDI spectra, making them useful for discrimination by LC-MS/MS, but not ZooMS. A1T66/67 (position 910-934, Red deer - GETGPAGR**P**GEVGPPGPPGPAGEK (m/z:2216) ; Elk - GETGPAGR**A**GEVGPPGPPGPAGEK) contains a SAP after a tryptic cut site. A proline after a tryptic cut site is known to increase the frequency of missed cleavages at the site [27]. This is observed in the LC-MS/MS data with a high percentage of missed cleavages in the red deer peptide, but none in the elk peptide.

Deamidation

Deamidation levels of asparagine (Asn) and glutamine (Gln) amino acid residues were examined to assure protein authenticity. Asn and Gln naturally deamidate over time, influenced by factors such as temperature and moisture. In this case, we compared the levels of deamidation of the collagen and other assumed authentic proteins (such as COL3, the bovine version being in MaxQuant's default contaminant list) with those detected and known to be common laboratory contaminants. In all cases, the peptides from the samples showed much more deamidation than the modern contaminants, supporting their authenticity (Supplementary Figures 27 and 28). While still low, the deamidation rates of the contaminants vary a lot more than the samples, probably due to the much lower contamination peptide counts that the calculations are based on.

Supplementary Note 5. Bone Density - ribs vs. long bones

Kurt J. Gron and Theis Zetner Trolle Jensen

The skeletal element of heavily worked artefacts made from either ribs or long bones is easily identified, due to gross morphological differences in shape, structure, and thickness. However, the reasons as to why humans preferred one over the other remain unclear. This is because any *a priori* assumptions about the choice of raw material for the manufacture of bone points almost certainly ignore various knowable and unknowable factors related to suitability for purpose. In the first instance, long bones and elements of the axial skeleton, such as ribs, likely have very different mechanical qualities, mostly predicated on differences in Bone Mineral Density (BMD)[37]. Long bones of the appendicular skeleton, for example, are generally denser than ribs, vertebrae, and other irregular-shaped elements more common in the axial skeleton; a fact often reflected in their differential survival in the archaeological record [38]. This density will certainly affect their malleability if treated, so depending on the desired result, ribs may have been more appropriate than other elements for this particular purpose, despite the apparent strength advantage of long bones; ribs and other skeletal elements can be straightened if heated and soaked [39]. There is also a degree of intra- and inter-taxonomic variability even within a single skeletal element. For example, ribs from the anterior part of the trunk have a different form than those from the posterior part. Furthermore, the same rib will have a different curvature in animals of different sizes, so that a red deer and an auroch of similar body size may have similarly curved ribs, whilst two animals of the same taxon might have divergent element morphology if there are significant differences in the circumference of the rib cage. It is almost certain that multiple considerations were taken into account when selecting raw material for the manufacture of the bone points, and these considerations were likely independent of any consistent preference for one taxon, one element, or one part, of a bone than another.

Supplementary Note 6. Isotopic analysis of collagen from bone points

Kurt J. Gron and Theis Zetner Trolle Jensen

Data acquisition

Samples with the prefix “OxA” were analyzed at the University of Oxford’s Radiocarbon Accelerator Unit. Stable isotope measurements for samples OxA-38,090, 38,092, 38,199, P46206, 38,665, and X-2807-20 were performed on a Fisons NA2000 elemental analyser coupled to a PDZ Europa 20-20 Isotope Ratio Mass Spectrometer. The remaining samples were analyzed on a Sercon elemental analyser connected to a Sercon 20-22 isotope ratio mass spectrometer. All samples were measured relative to an in-house l-alanine standard routinely externally characterized by third parties.

Samples with the prefix “Ua” were analyzed at The Tandem Laboratory at Uppsala University also marked with an asterisk * in Supplementary Dataset 1. Isotope ratios were

determined on an Elementar ISOTOPE select elemental analyser coupled to a Elementar isoprime precisION isotope ratio mass spectrometer. Measurement accuracy was checked against internal sorghum flour, wheat flour, and protein standards, which were calibrated against international standards IAEA-C7 and IAEA-C6 as well as an internal laboratory standard.

Results

Twenty-three carbon ($\delta^{13}C$) and nitrogen ($\delta^{15}N$) bone collagen isotope ratios were obtained. Using ZooMS, eight of these were attributed to aurochs or bison (henceforth referred to as bovines) and eleven to Eurasian elk or red deer (henceforth referred to as cervids) (Supplementary Figure 26). All samples fell within the acceptable atomic C:N range of 2.9-3.6 [28], indicating a low likelihood of diagenesis. However, four of these $\delta^{13}C$ values (Ox-38342, 38343, 38337, 38341), dating to the period prior to the hiatus, diverge from the main dataset and must be treated with caution. In a broader context, and with available contemporary archaeological northern European large herbivores, these values are substantially more negative [29]. Both the cervids ($\delta^{13}C$ -24.3‰ and lower) and bovine samples (-25.1‰) are lower than values obtained on the same taxonomic groups for the entirety of the Eurasian Holocene [30]. Substantial divergence from the main dataset is not typically a reason for caution; however, there are no recorded archaeological $\delta^{13}C$ values from northern Europe lower than -24.31‰ for the taxa in question (dIANA database, https://oasisnorth.org/diana.html, accessed 3/9/2019). For these reasons, these four values will not be discussed further, but we acknowledge that they are exceptionally low (but see Supplementary Information 8 for an extended discussion).

The remaining bovine (n = 11) and cervid (n = 8) $\delta^{13}C$ values range from -24.3 to -21.7‰ (mean -23.2‰) and -23.6 to -20.8‰ (mean -21.9‰) respectively. $\delta^{15}N$ values have a broader range, with bovine and cervid values between 1.5 to 6.3‰ (mean 4.8‰) and 1.7 to 7.2‰ (mean 4.3‰). There is a net decrease in average $\delta^{13}C$ before and after the hiatus in both bovines (-21.7 to -23.4‰) and cervids (-21.4 to -22.5‰), whilst average bovine $\delta^{15}N$ increases (2.7 to 4.8‰) and average cervid $\delta^{15}N$ decreases (5.2 to 3.4‰). In comparison with 329 well-dated large herbivore bone collagen carbon and nitrogen isotope values (Fig. S26) from across northern Europe recently published by [30] there is a similar net decrease in $\delta^{13}C$ on either side of our apparent hiatus. However, our $\delta^{15}N$ data do not clearly relate to any long-term processes as proposed for the North European material by [30], and no consistent inter-taxonomic trends are discernible (Supplementary Figure 26).

Supplementary Note 7. Water Level Fluctuations in Danish/German/Swedish Straits

Ole Bennike

During the final drainage of the Baltic Ice Lake at about 11,700 cal. BP, the water level dropped by c. 25 m over a few years [31]. The water level in the Danish/German/Swedish straits reached a low stand during the early Preboreal, and following this drainage event, peat bogs, local lakes, and forested areas became widespread in the straits. As the relative sea level in Kattegat began to rise, a fjord with brackish water formed in the northern Øresund. The ongoing eustatic sea-level rise led to increased salinity and the fjord became larger. However, the threshold in Øresund, with a present threshold depth of 7 m, was probably not flooded until at about 8,000 cal. BP [32].

In the Great Belt, the oldest dated marine shells gave an age of ca. 8,100 cal. BP [33]. In the Little Belt, the oldest shell date is ca. 7,700 cal. BP [34] and in Mecklenburg Bay the oldest reported age is ca. 7,600 cal. BP. However, it appears that water level began to rise long before these ages, which resulted in the formation of large lakes. The youngest lake deposits in the Little Belt are dated to ca. 8,500 cal. BP, at this time the lake level was ca. 13 m below sea level. The lake phase was followed by a brackish-water phase that lasted ca. 800 years, during this time period, salinity was too low for marine molluscs to live in the Little Belt.

In the central parts of the Great Belt, between Zealand and Funen, fluvial deposits have been dated to about 10,650 to 10,250 cal. BP [35]. The fluvial phase was followed by a lake phase that lasted until ca. 8,800 cal. BP. During this phase, the lake gradually became larger and larger and flooded wide areas, this was followed by a brackish-water phase. The development of Mecklenburg Bay and the Femern Belt was probably similar to the development in the Little Belt, but a river probably also existed in the Femern Belt as in the Great Belt, as depicted on palaeogeographical maps by Jensen et al. [36].

Supplementary Discussion 8. Potential Causes for Absence of Bone Points

Theis Zetner Trolle Jensen, Liam Lanigan, Erika Rosengren, & Kurt J. Gron

Based on southern Swedish lake sediment cores [40–42], the Boreal may have been particularly dry and saw the lowest water levels of any point during the Holocene. This evaporation of lakes with sediment infill may have started even earlier in eastern Denmark [43]. Palynological records also provide corroboration that lake levels shifted appreciably during the Early Holocene [4,5], and around the start of the Boreal period. Changes in vegetation are evidenced by a northern expansion of hazel forest, likely taking advantage of now reduced lacustrine coverage to colonise former lake beds. Lower concentrations of stagnant ice during the Boreal period could go some way to explain a drier climate. Other factors include a warming climate and the role of 'biological engineers' such as beavers.

For species that are associated with wetland habitats, such as elk and aurochs, these changes in conditions would have almost certainly had a detrimental effect. We must be aware; however, that the lack of dated animal remains from this period could possibly be a matter of preservation, and/or other types of sample biases (see [44]). Since wetlands constitute the optimal conditions for bone preservation, a dearth of remains may reflect subsequent challenging taphonomic conditions. Several analyses of osteological remains have lent support to an impoverishment of faunal material on Zealand [45 and cited references].

Alternatively, palaeogeographical changes may help explain the 10 ka hiatus, with Zealand, Lolland, and Falster being separated from the rest of Denmark at about 10 cal. ka BP due to a rising shore level [34]. At this time, large lakes also began forming in the Femern Belt and the Great Belt [33]. Despite this, it is likely that a land bridge still connected Zealand to southern Sweden [32]. Contemporary animal populations in Scania are still accounted for in the archaeological record, so it can be claimed that inundation of the sounds is not a viable explanation for why the large mammals seemingly did not migrate to or from eastern Denmark. Finally, at c. 8,000 cal BP several large ungulates and carnivores disappeared altogether from eastern Denmark [46], possibly as a result of rising sea-levels (Littorian transgression) turning Denmark (apart from Jutland) into an archipelago [47].

Despite the small sample size, the stable carbon isotope data is consistent with niche partitioning between species, as well as signs of environmental change, although the value obtained from a single bovine individual seems to indicate that it was exploiting a similar feeding niche to the cervids. Before ca. 10 ka BP, the $\delta^{13}C$ values vary considerably, but after 9,5 ka BP, decreasing $\delta^{13}C$ values indicate an environmental shift, from an open environment to a more closed setting, probably in part due to the canopy effect[5]. The $\delta^{13}C$ values may, therefore, indicate that the mammals were feeding within an increasingly forested landscape after 9,5 ka BP. This probably reflects animals favouring the edges of wetlands and forest clearings. Within this setting, niche partitioning based on the preferred feeding ecology of the taxa results in higher cervid $\delta^{13}C$ values than the bovines (Hofmann 1989).

Following the hiatus, the manufacture of bone points changes considerably. Could this be a reflection of this greater environmental shift? There are a more varied species selection and choice of raw material. This may reflect changing habitat that favoured these species, as open hazel-pine (*Corylus* sp. and *Pinus* sp.) forests were replaced after the hiatus with more closed and darker forests dominated by lime, oak and elm (*Tilia* sp., *Quercus* sp., and *Ulmus* sp.) [48]. Conversely, it could reflect a shift in hunting strategy to the more localized environment, although there is some $\delta^{13}C$ overlap before and after the hiatus in the radiocarbon dates. Wear on the teeth of aurochs from this period suggests an abrasive diet, consistent with a major proportion of their diet being based on grasses [49]. The vegetational development might increasingly have restricted grazing species to the fringes of the forest (i.e. the 'marginal-effect') making them especially vulnerable to human hunting [50].

There is no appreciable change in $\delta^{15}N$ before or after the hiatus, but there is a rather large range of variation in the $\delta^{15}N$ values obtained herein. $\delta^{15}N$ values record the animals' dietary protein through the filter of its behavioural ecology, and therefore will at least in-part reflect available browsing and grazing environments. The taxa represented within the resolution of our ZooMS results encompass a broad range of preferred feeding habitats and feeding types, including bulk feeders, intermediate feeders, and concentrate selectors [51]. These feeding types are at least in part influenced by the animals' digestive systems, a factor known to affect $\delta^{15}N$ values in herbivores of different gut complexities (Bocherens et al. 1996). Our $\delta^{15}N$ values are much more variable (Bovine, 4.8‰ and Cervid, 5.5‰) than values for single herbivore taxa in multiple environments (ca. 2-3‰) [52], and therefore probably reflect both physiological differences in addition to reflecting any environmental change. This relationship between high variability of nitrogen levels alongside landscape changes remains uncertain, and possibly indicates erroneous results for a few samples although all C/N values were within the accepted. More intriguingly, the unexpected results seen in a few samples might be indicative of physiological stress due to a disruption of 'normal' ecological conditions leading to unusual resource exploitation by local fauna.

Supplementary Figures

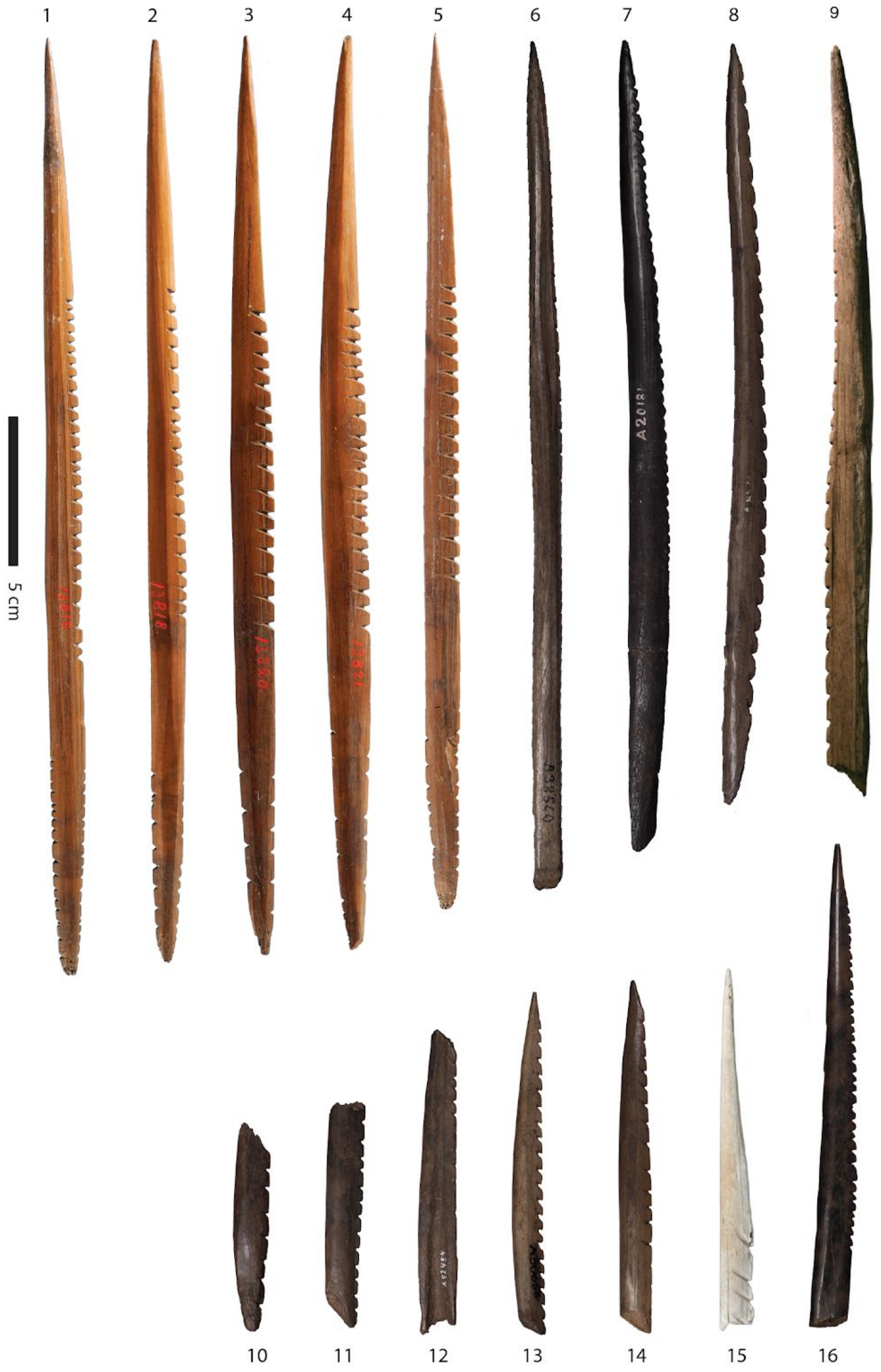

Supplementary Figure 1. Photos of bone points. 1: VHM13819, 2: VHM13818, 3: VHM13820, 4: VHM13821, 5: VHM13822, 6: A38560, 7: A20181, 8: A8524, 9: SLA2, 10:A52427, 11:52492, 12: 52454, 13: A50653, 14: A42153, 15: FP985, 16: A42156

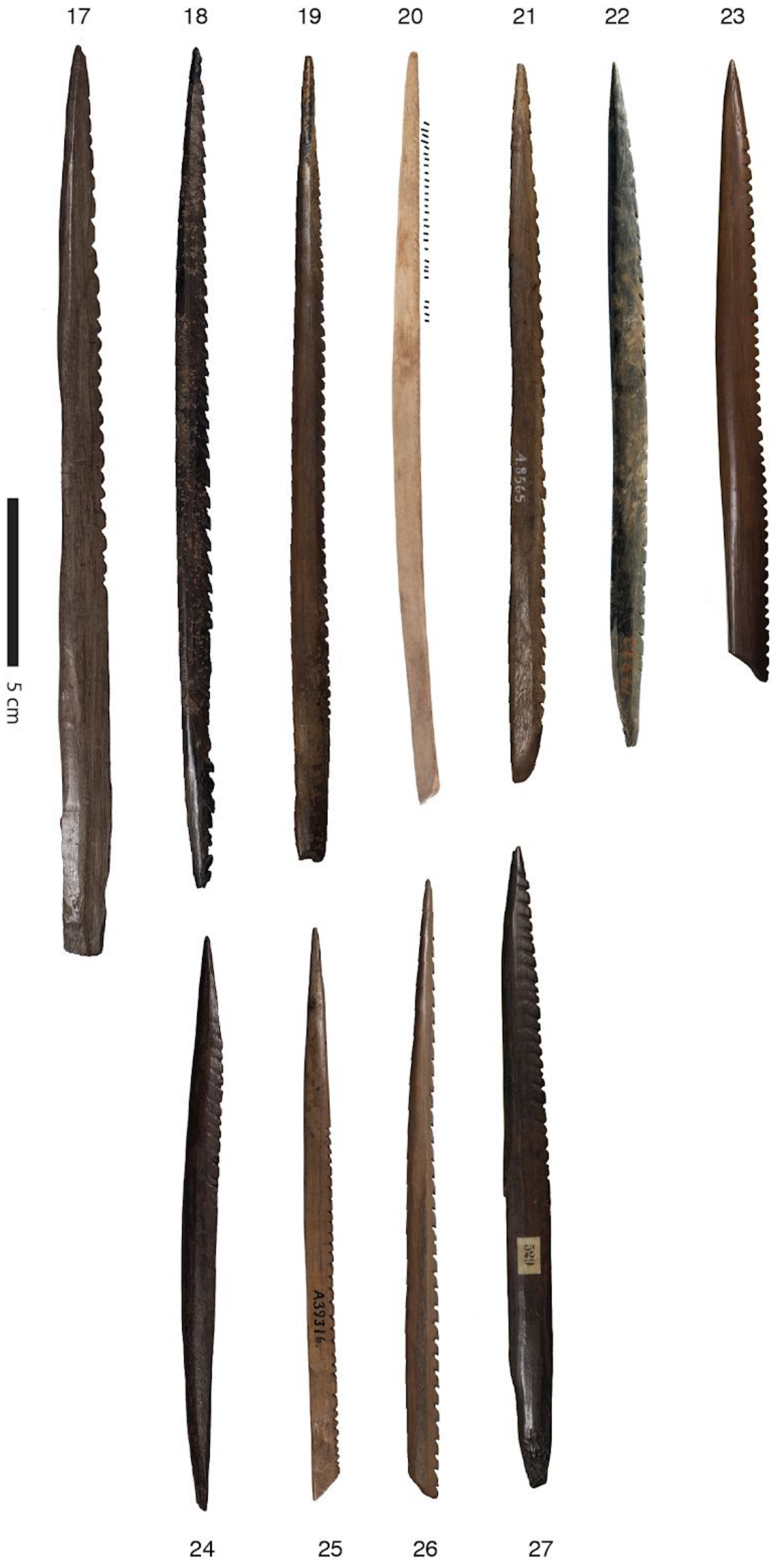

Supplementary Figure 2. Photos of bone points. 17: A20416, 18: A37811, 19: A42792, 20: 442, 21: A8565, 22: VHM14923, 23: A4763, 24: A42924, 25: A39316, 26: A16966, 27: A9055

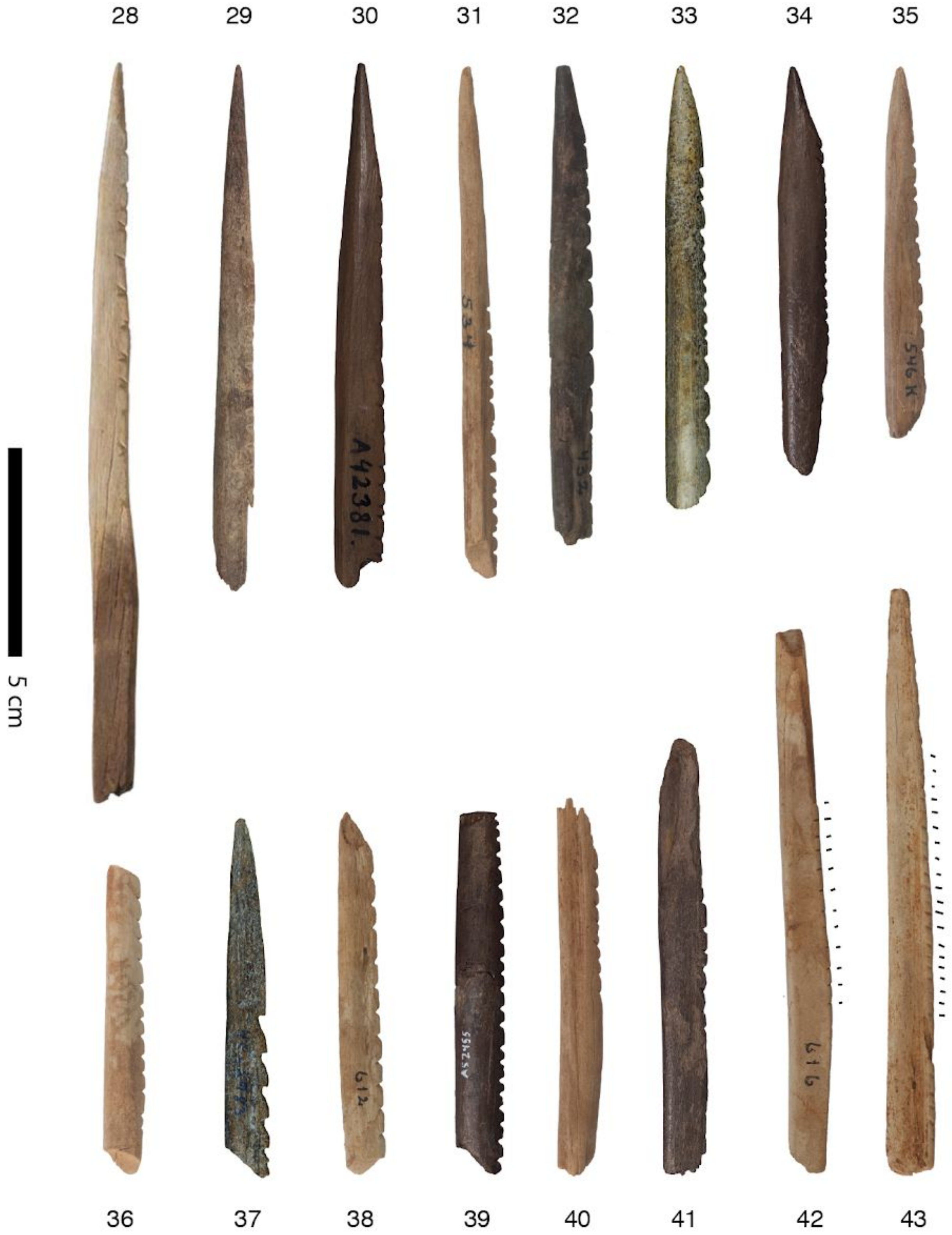

Supplementary Figure 3. Photos of bone points. 28: FP1469, 29: Ulk3, 30: A42381, 31: 534, 32: 432, 33: Skellingsted2, 34: A52426, 35: 546, 36: 438, 37: Skillingsted3, 38: 612, 39: A52445, 40: 562, 41: 52494, 42: 616, 43: 573

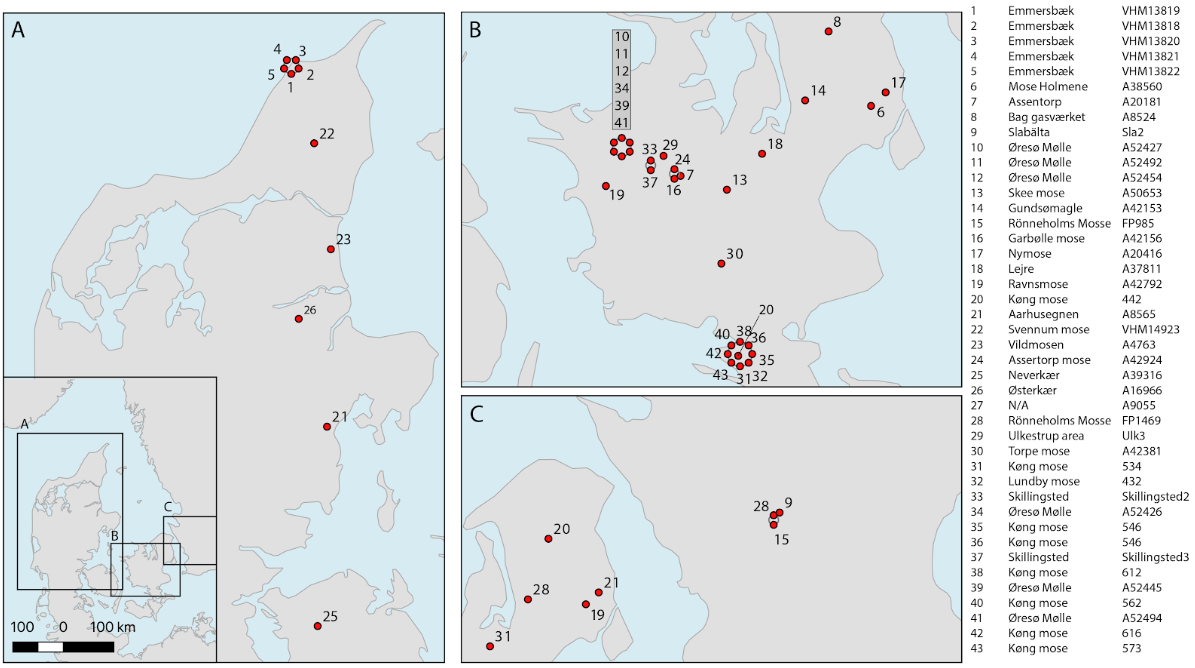

Supplementary Figure 4. Overview of approximate provenance of bone points 1-43 (N.b. Nr. 27 not shown due to no provenance).

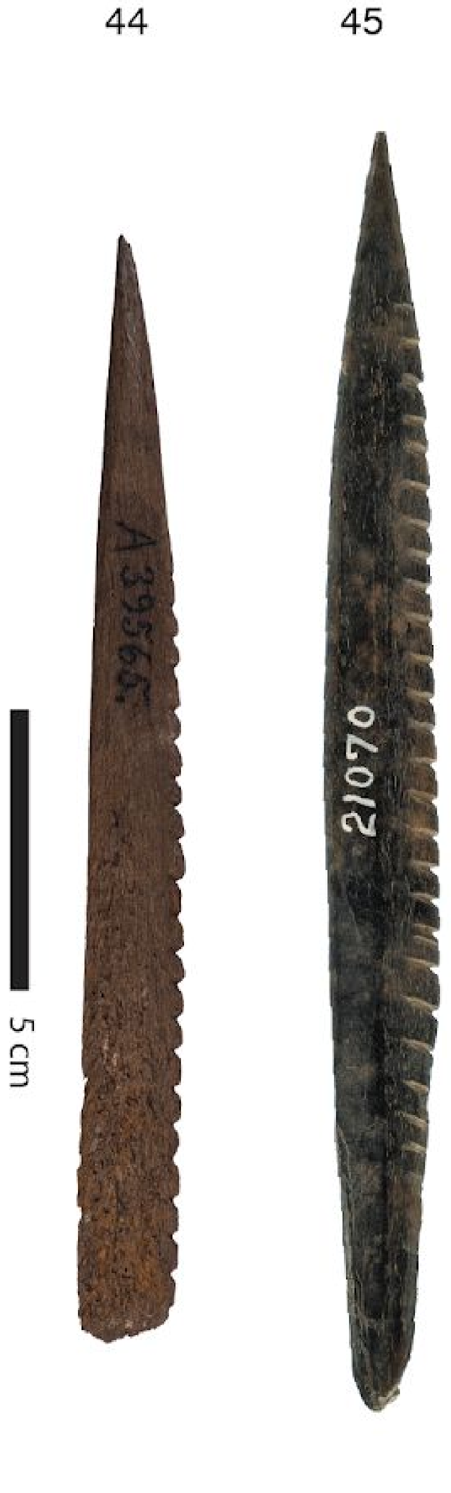

Supplementary Figure 5. Photos of bone points. 44: A39565, 45: VHM21070

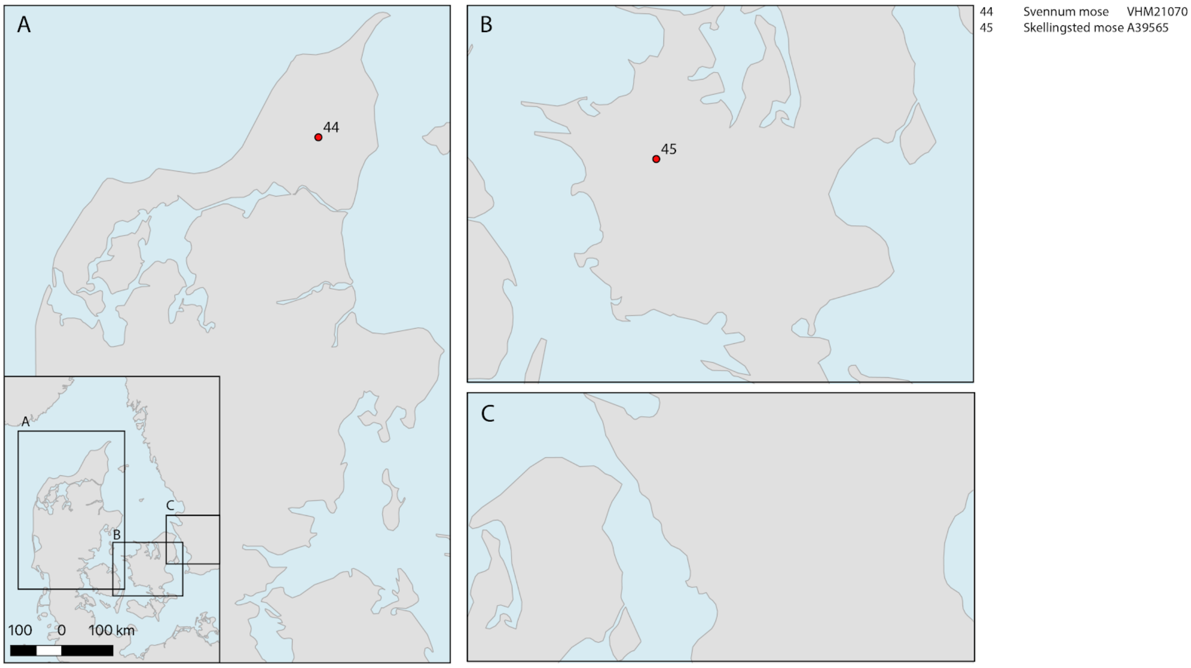

Supplementary Figure 6. Overview of approximate provenance of bone points 44-45.

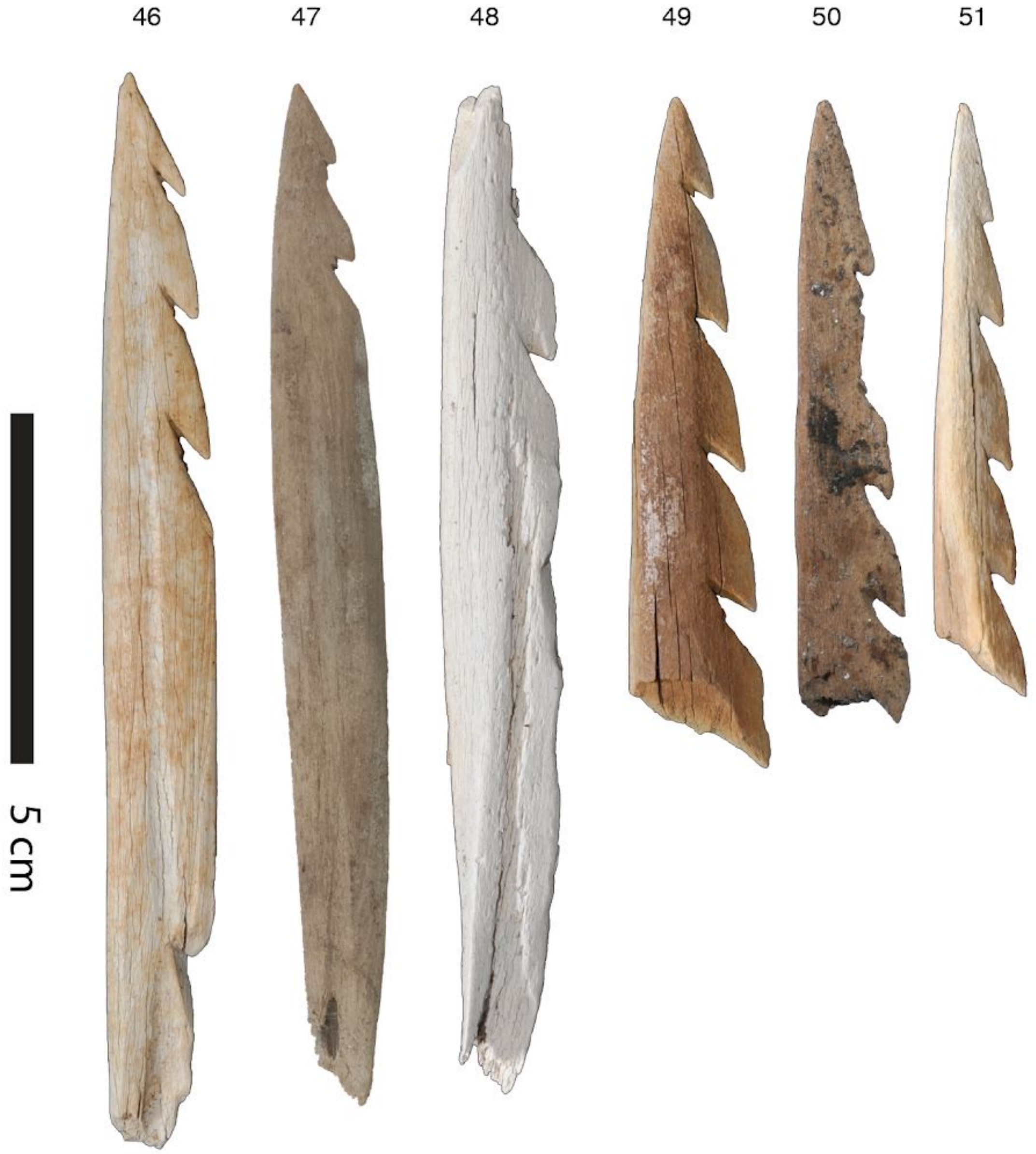

Supplementary Figure 7. Photos of bone points. 46: FP1507, 47: FP923, 48: FP1492, 49: FP1488, 50: FP982, 51: FP1483

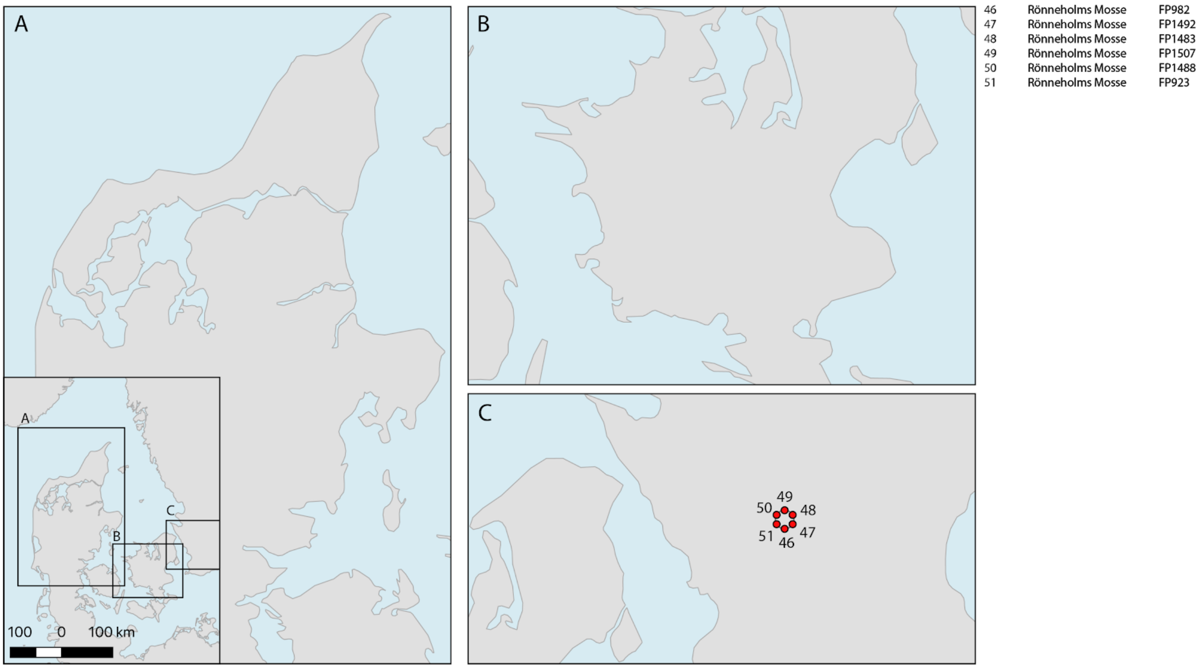

Supplementary Figure 8. Overview of approximate provenance of bone points 46-51.

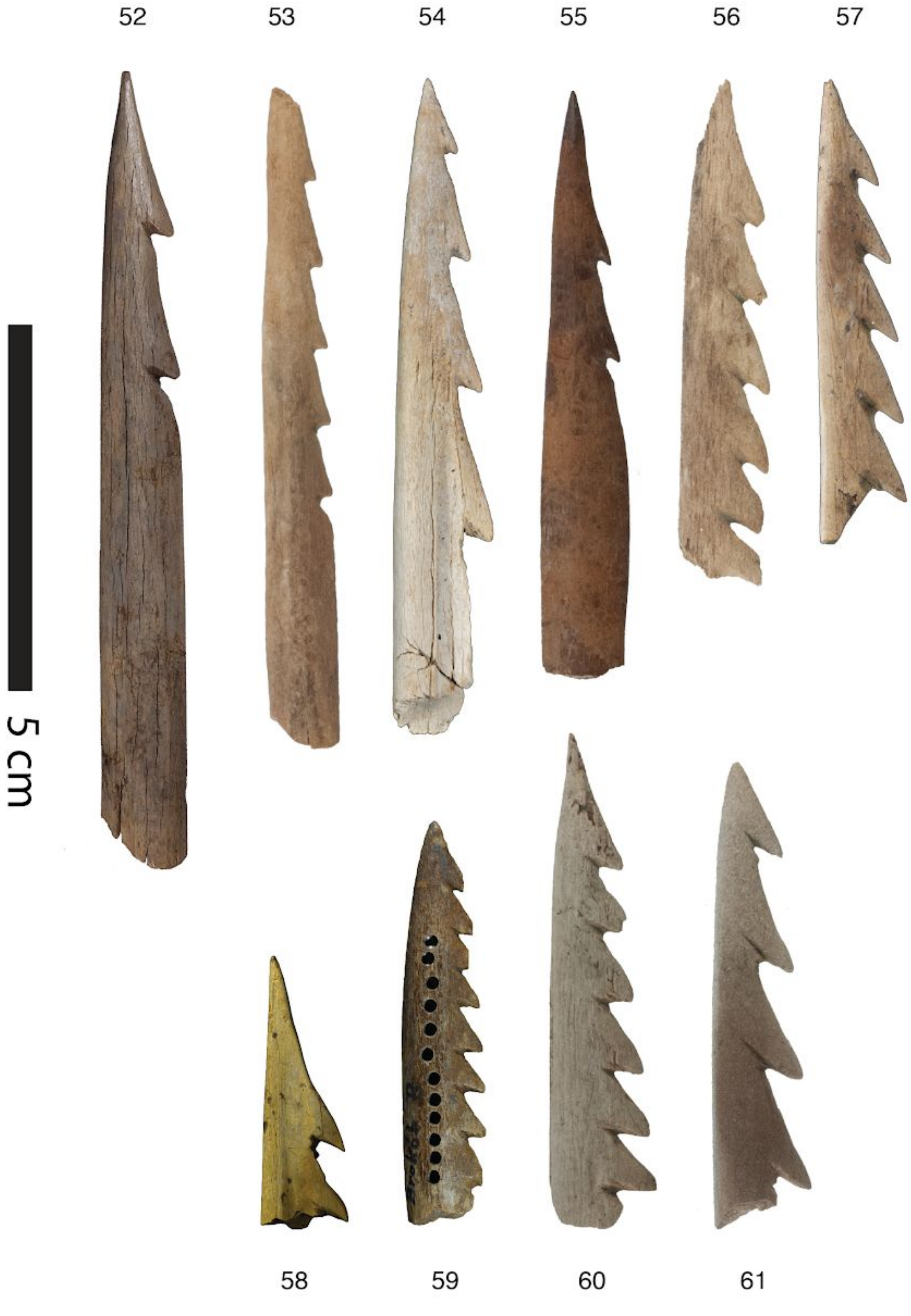

Supplementary Figure 9. Photos of bone points. 52: A40894, 53: 544, 54: FP1589, 55: Aamosen7, 56: FP1151, 57: FP1198, 58: FP132, 59: Brokøb B, 60: FP762, 61: FP1423

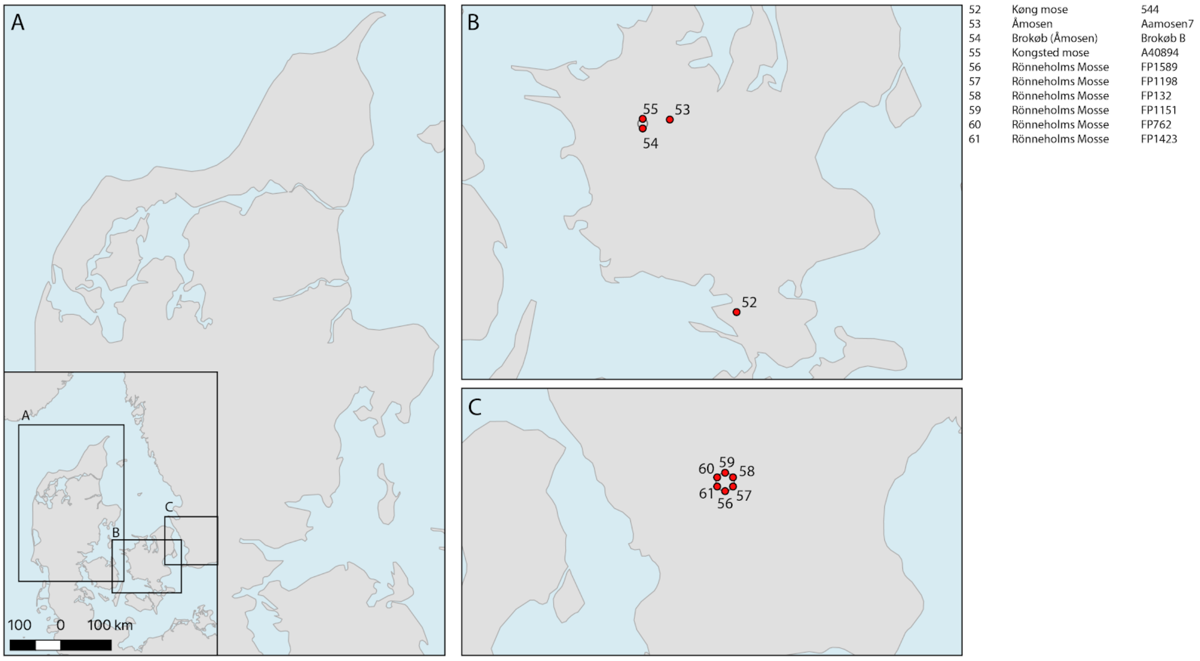

Supplementary Figure 10. Overview of approximate provenance of bone points 52-61.

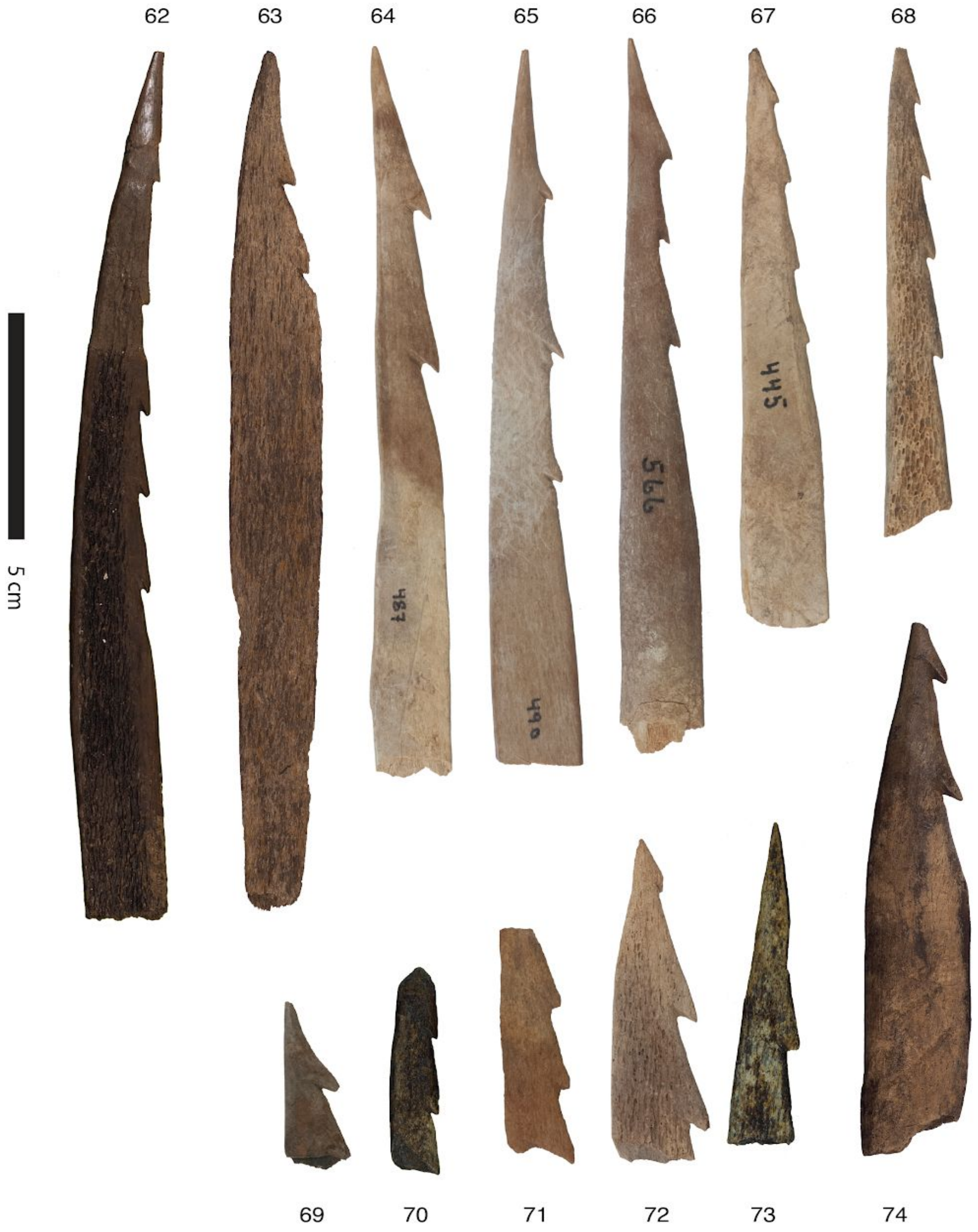

Supplementary Figure 11. Photos of bone points. 62: A49042, 63: A45770, 64: 487, 65: 490, 66: 566, 67: 445, 68: 606, 69: Aamosen17, 70: Øgårde4, 71: Aamosen6, 72: Ulk10, 73: Øgårde 3, 74: A45173.

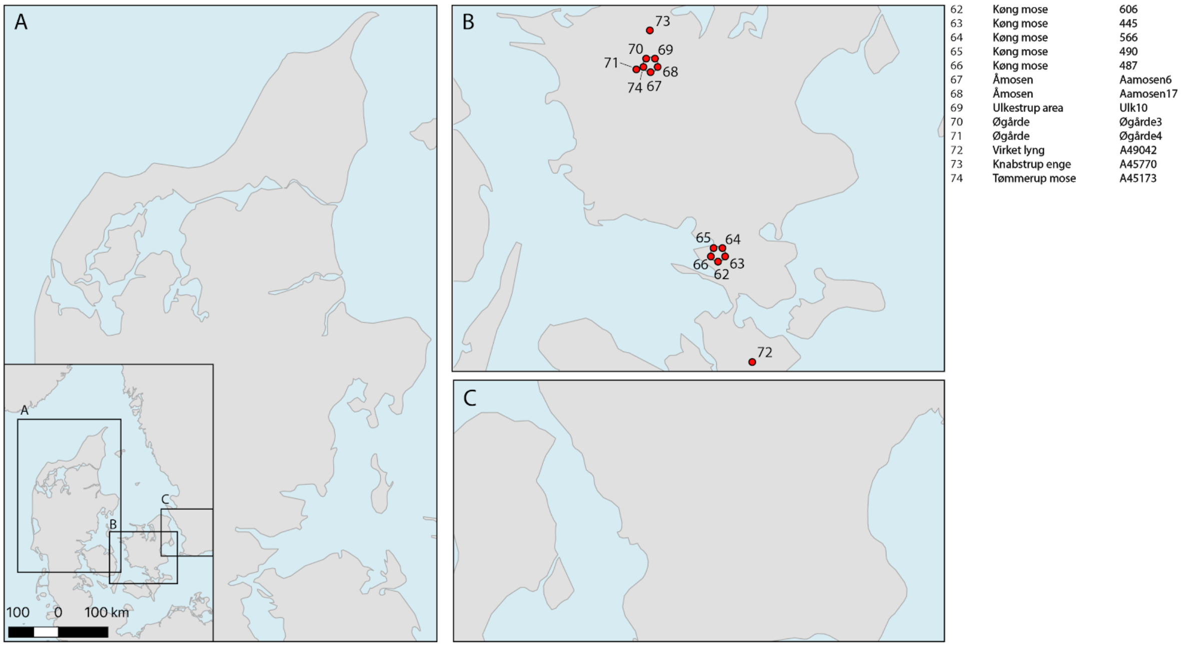

Supplementary Figure 12. Overview of approximate provenance of bone points 62-74.

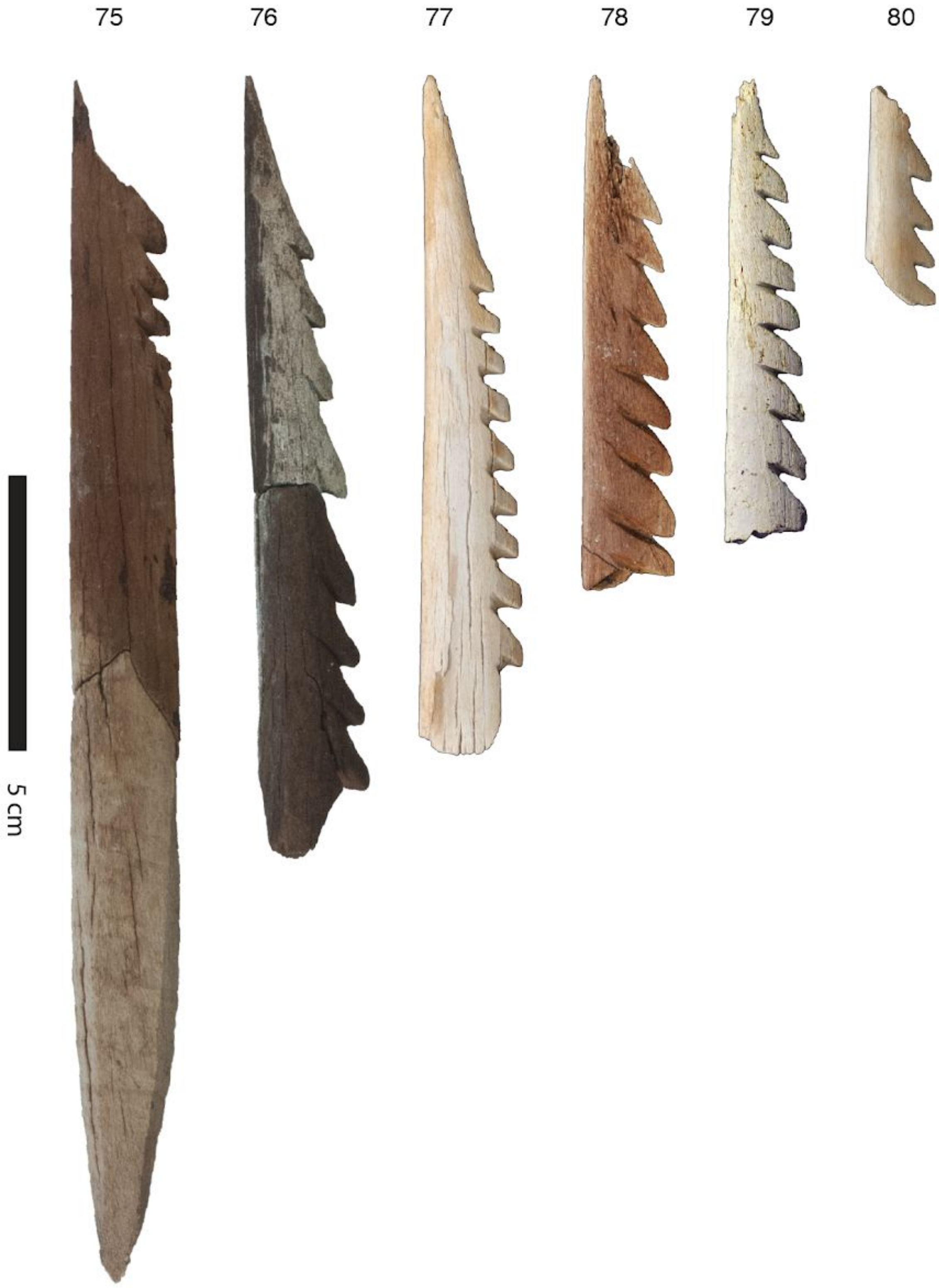

Supplementary Figure 13. Photos of bone points. 75: FP1516, 76: FP1377, 77: FP1470, 78: FP1516, 79: FP269, 80: FP1506.

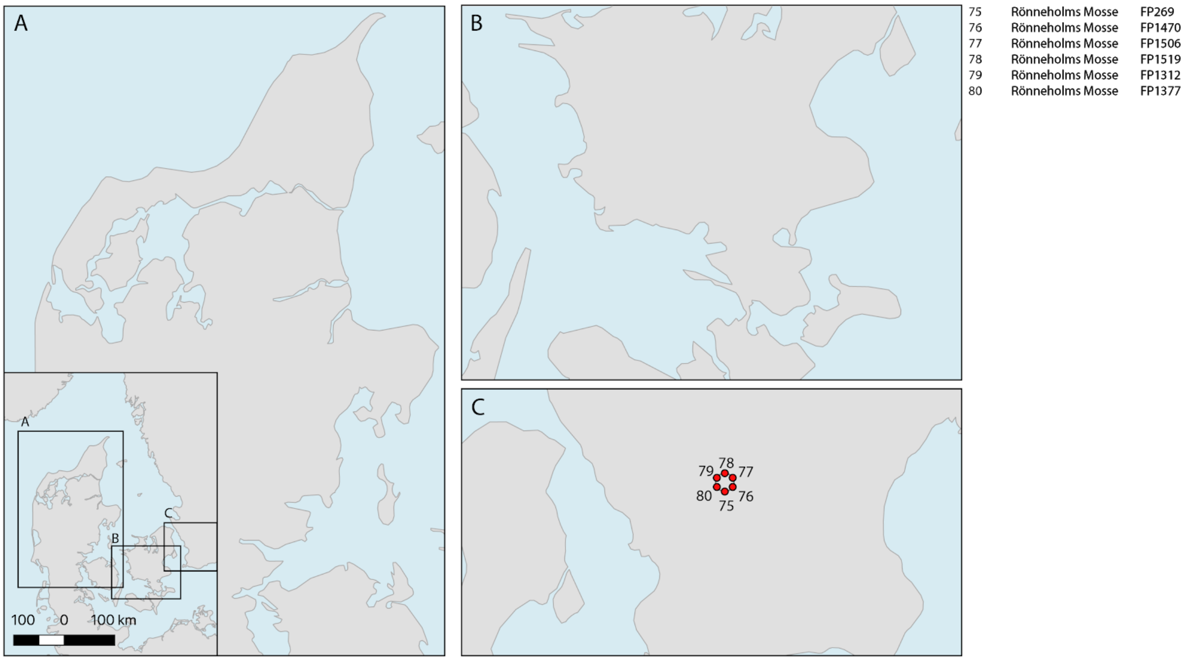

Supplementary Figure 14. Overview of approximate provenance of bone points 75-80.

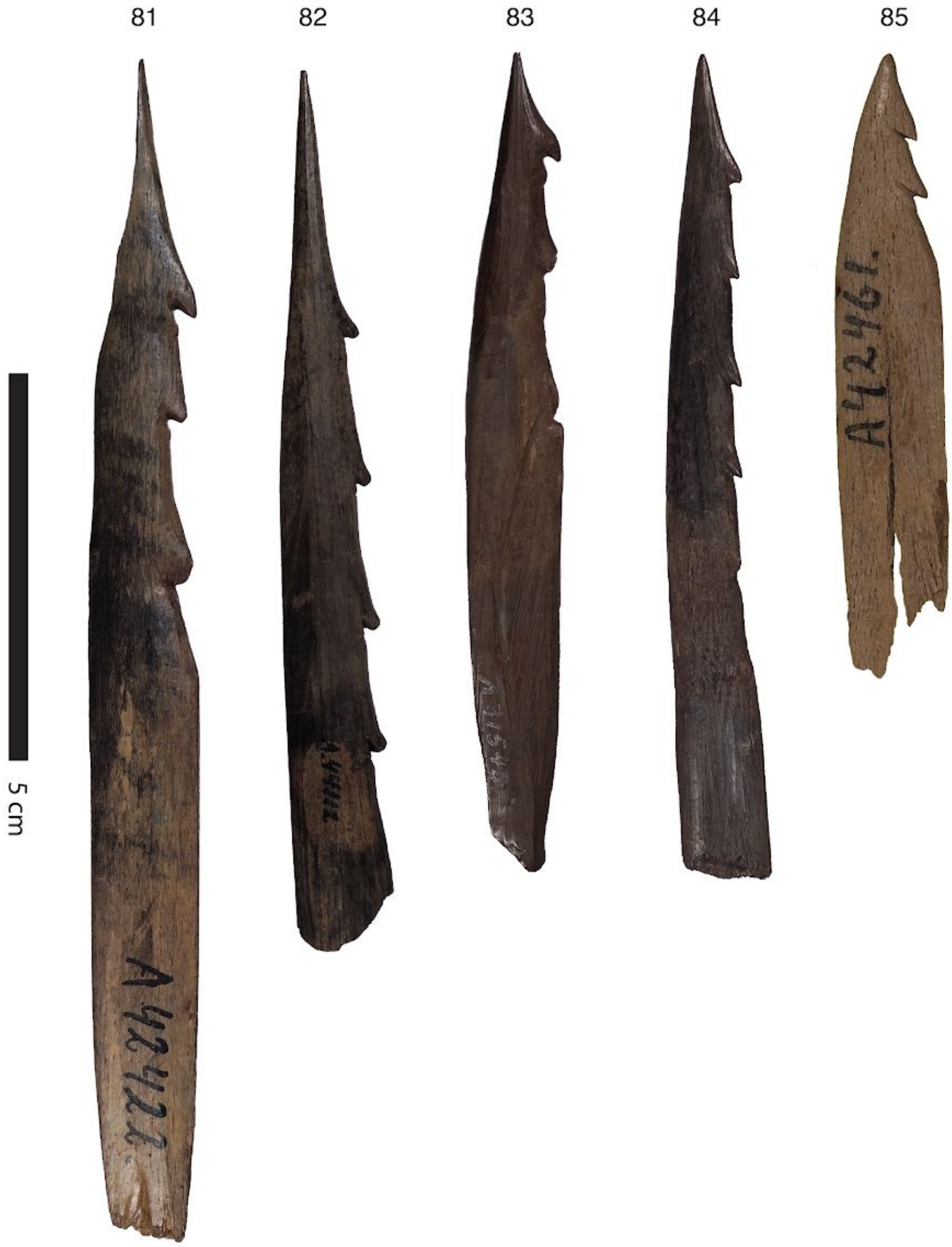

Supplementary Figure 15. Photos of bone points. 81: A42422, 82: A44112, 83: A31548, 84: A5126, 85: A42461

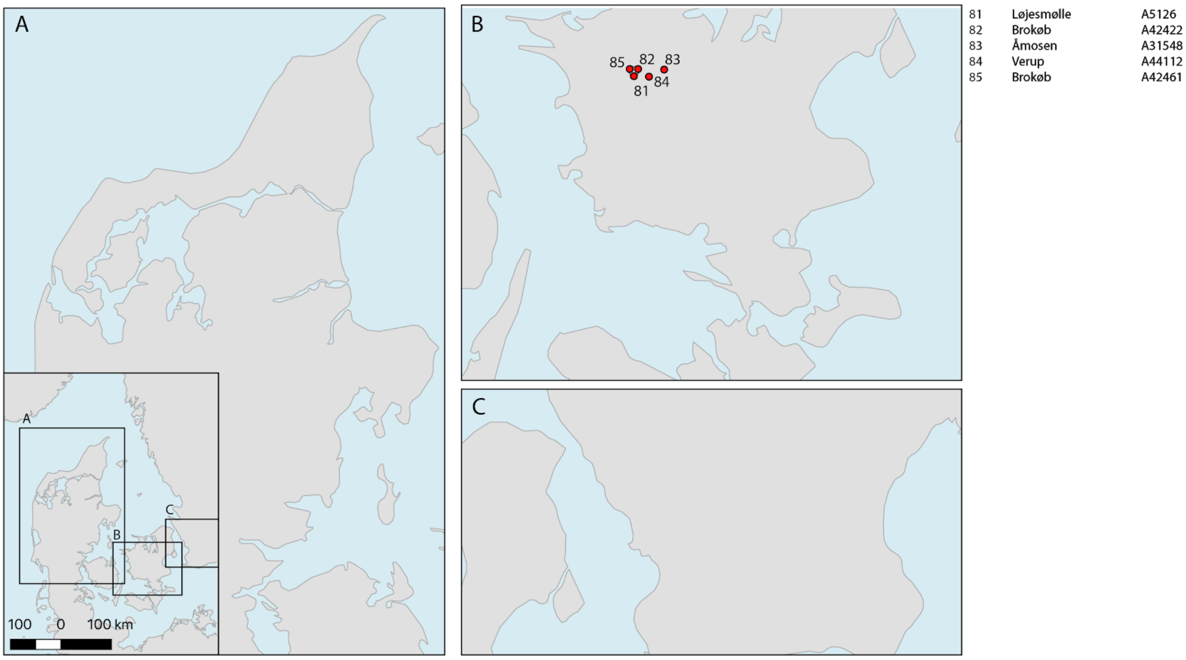

Supplementary Figure 16. Overview of approximate provenance of bone points 81-85.

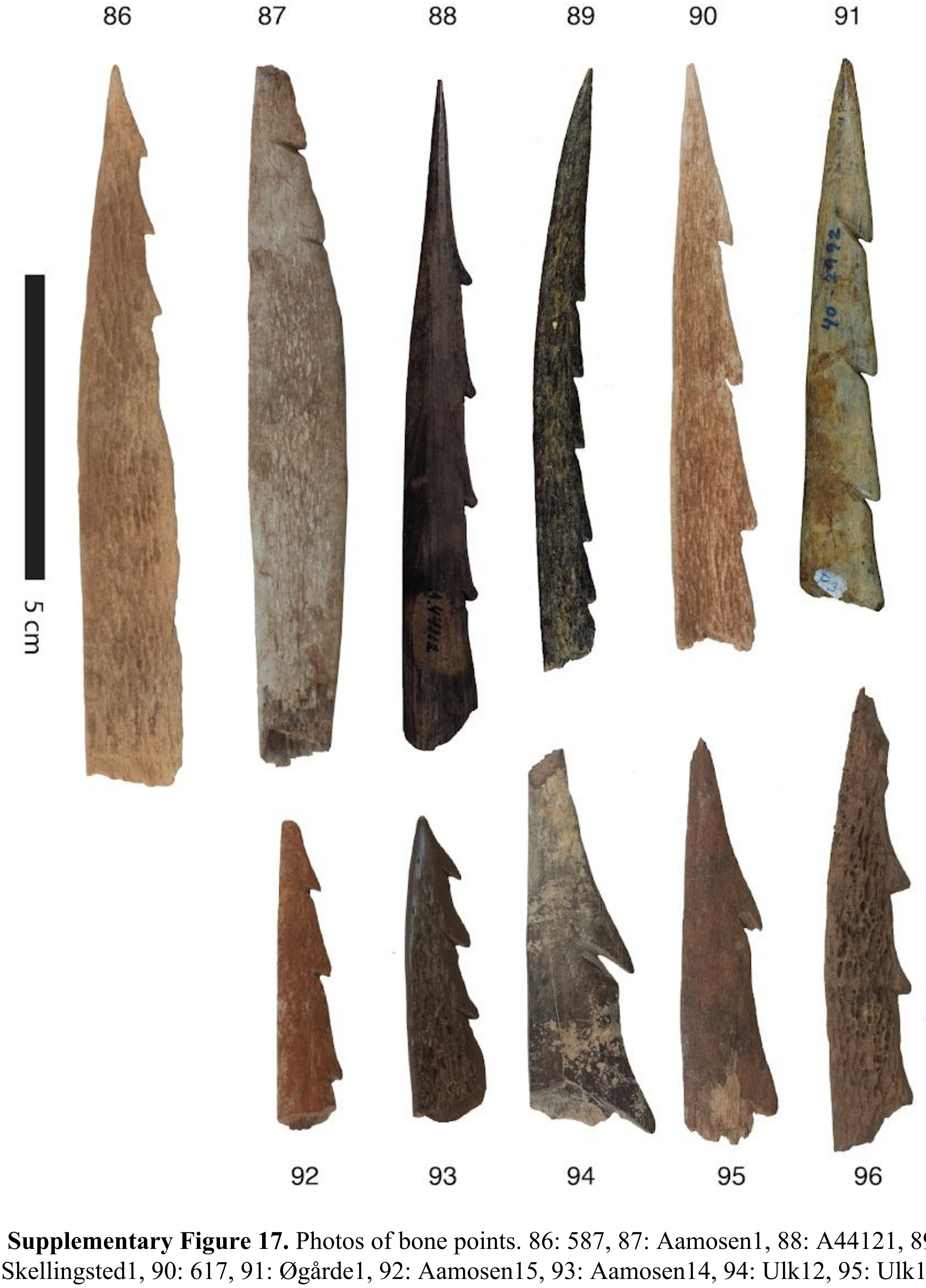

Supplementary Figure 17. Photos of bone points. 86: 587, 87: Aamosen1, 88: A44121, 89: Skellingsted1, 90: 617, 91: Øgårde1, 92: Aamosen15, 93: Aamosen14, 94: Ulk12, 95: Ulk11: 96: Aamosen10.

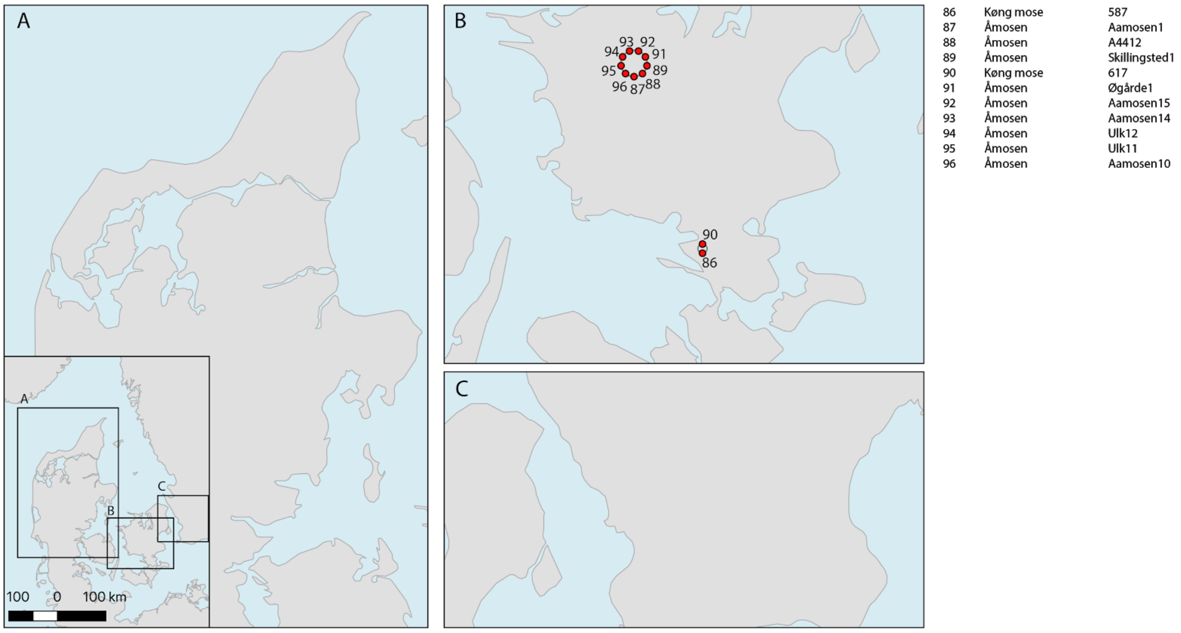

Supplementary Figure 18. Overview of approximate provenance of bone points 86-96.

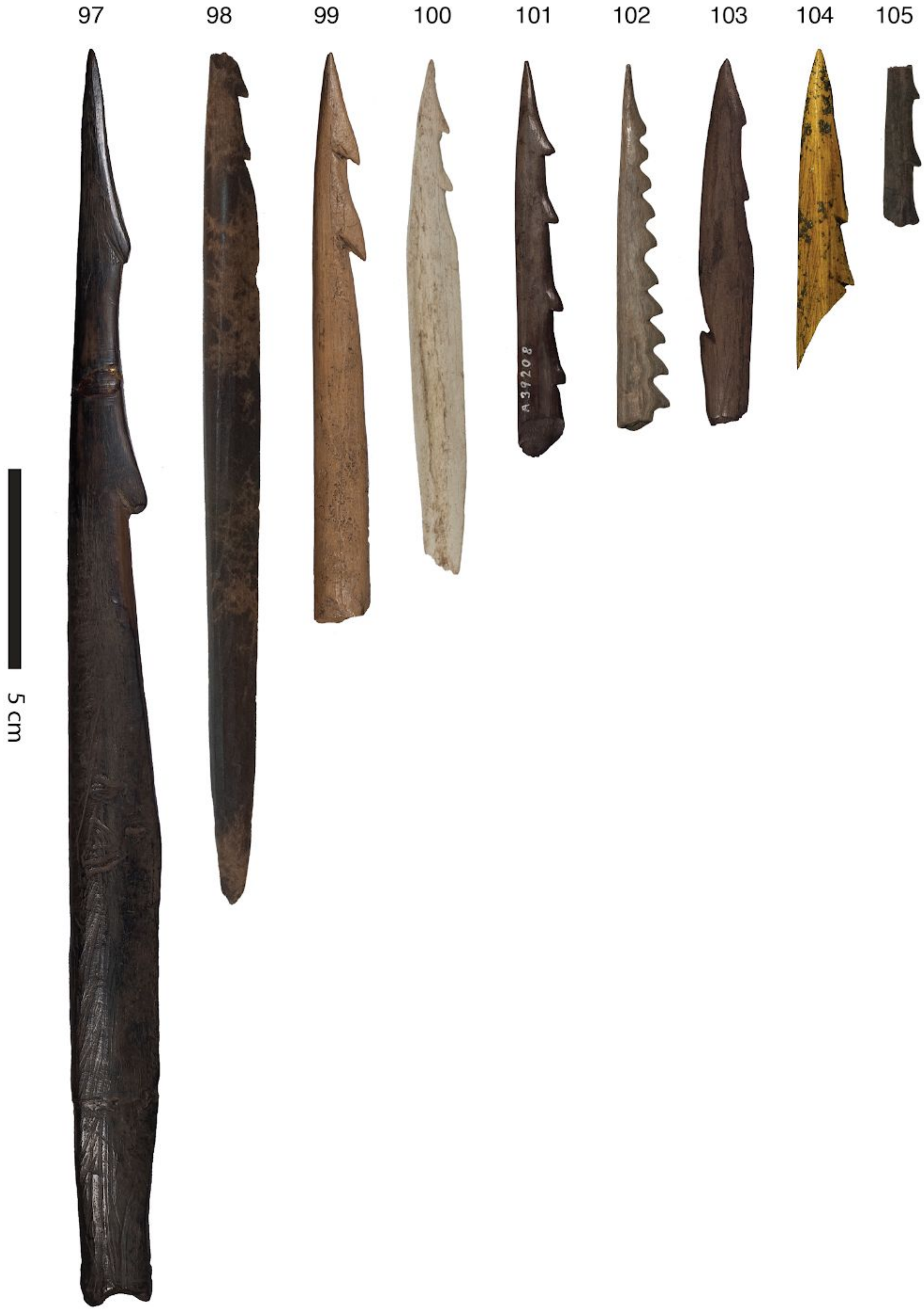

Supplementary Figure 19. Photos of bone points. 97: A14632, 98: Ulk1, 99: A31546, 100: FP1006, 101: A39208, 102: Sla1-2, 103: A38552, 104: FP37, 105: Aamosen18.

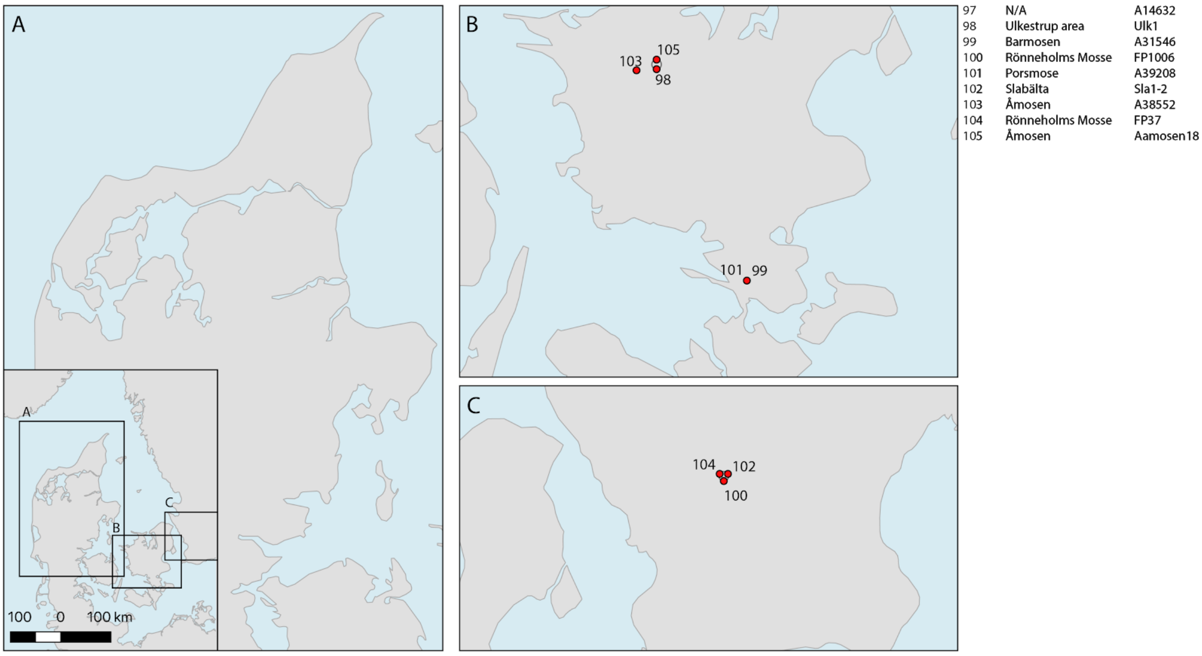

Supplementary Figure 20. Overview of approximate provenance of bone points 98-105 (nb. Nr. 97 not shown on map due to no provenance).

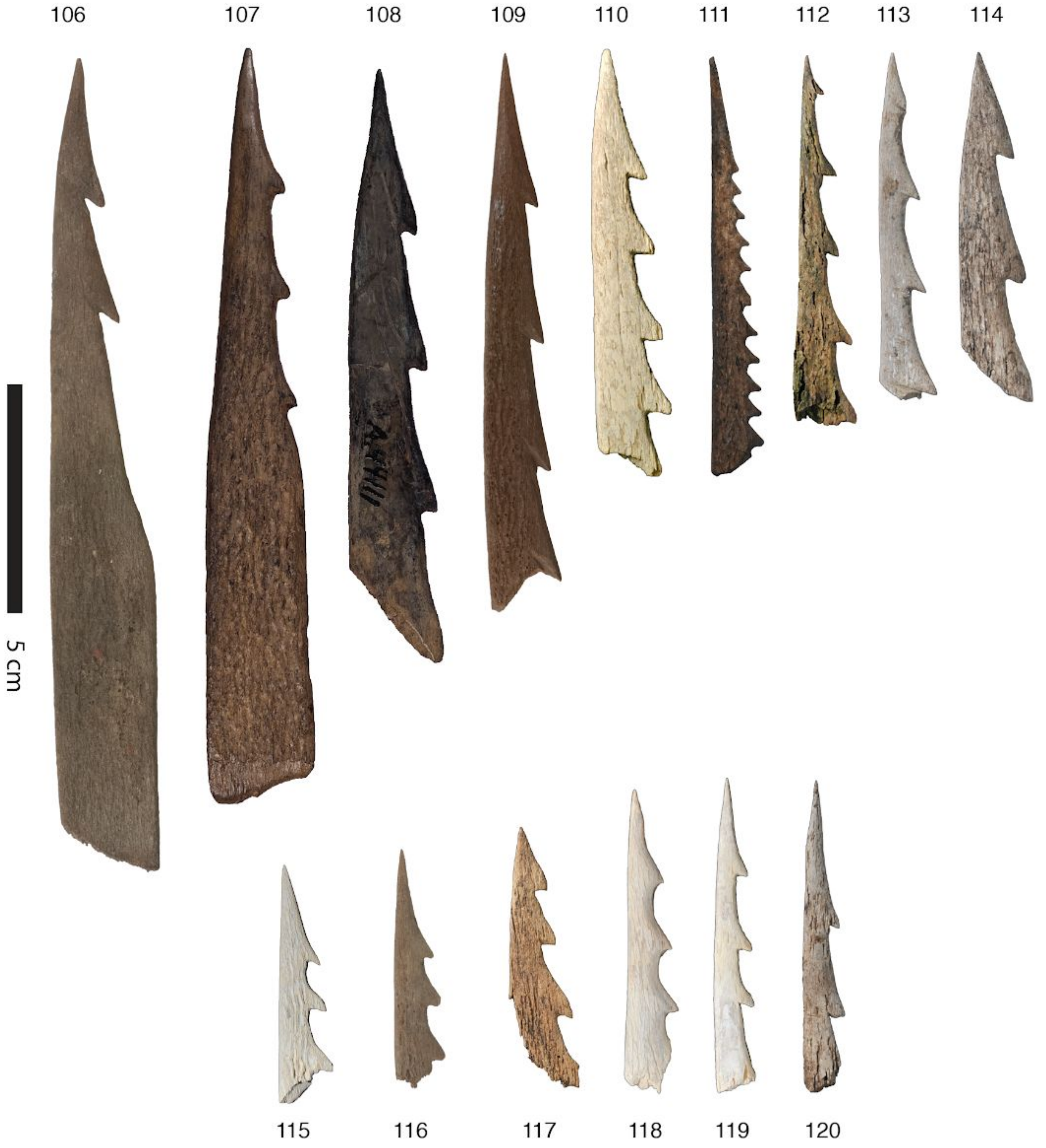

Supplementary Figure 21. Photos of bone points. 106: 715, 107: A42424, 108: A44111, 109: 427, 110: FP746, 111: FP1204, 112: FP1627, 113: FP1247, 114: FP1220, 115: FP1536, 116: FP107, 117: FP482, 118: FP1501, 119: FP1445, 120: FP956.

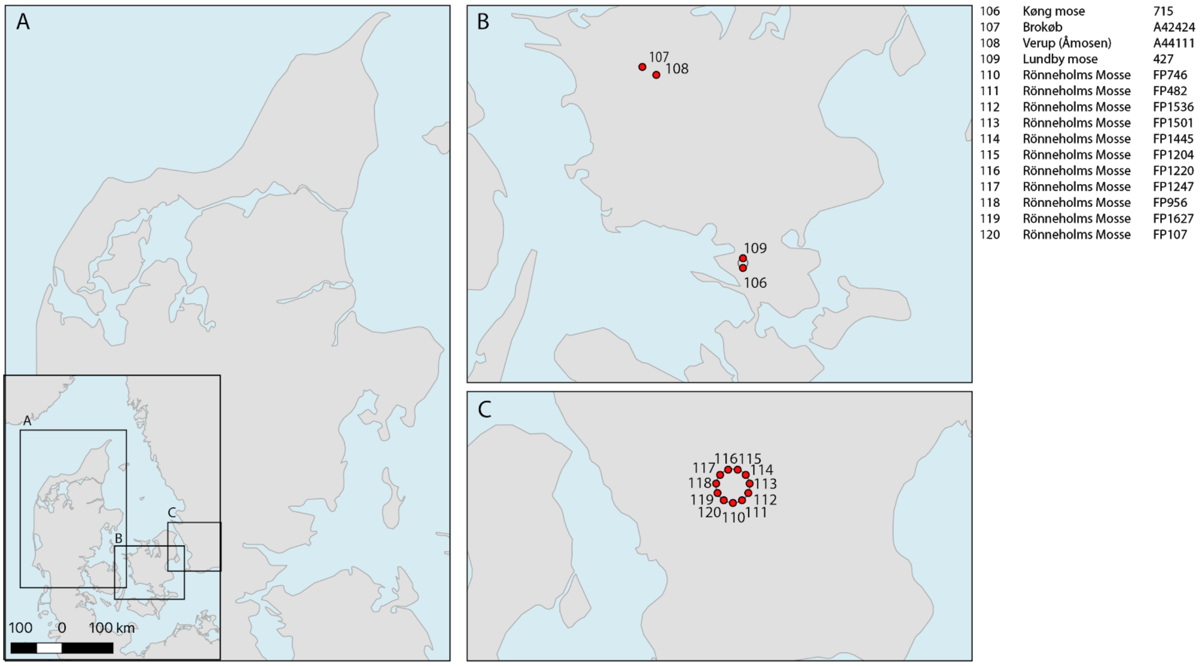

Supplementary Figure 22. Overview of approximate provenance of bone points 106-120.

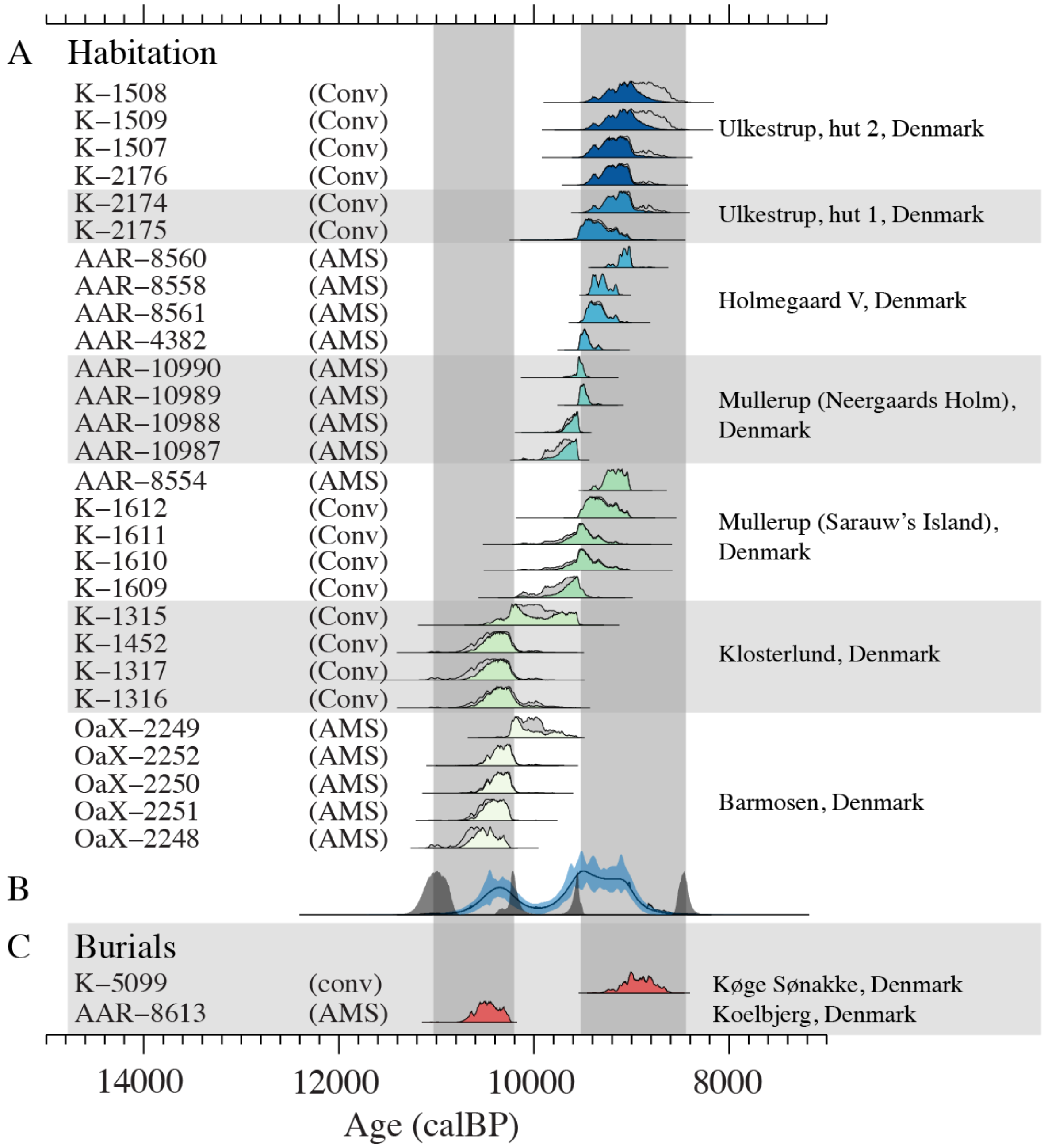

Supplementary Figure 23. Radiocarbon dates from the Early Mesolithic in Denmark. A) associated with habitation, B) KDE model showing activity, C) stray found human skeletons (nb. No context information for Køge Sønakke, which was revealed through seafloor dredging).

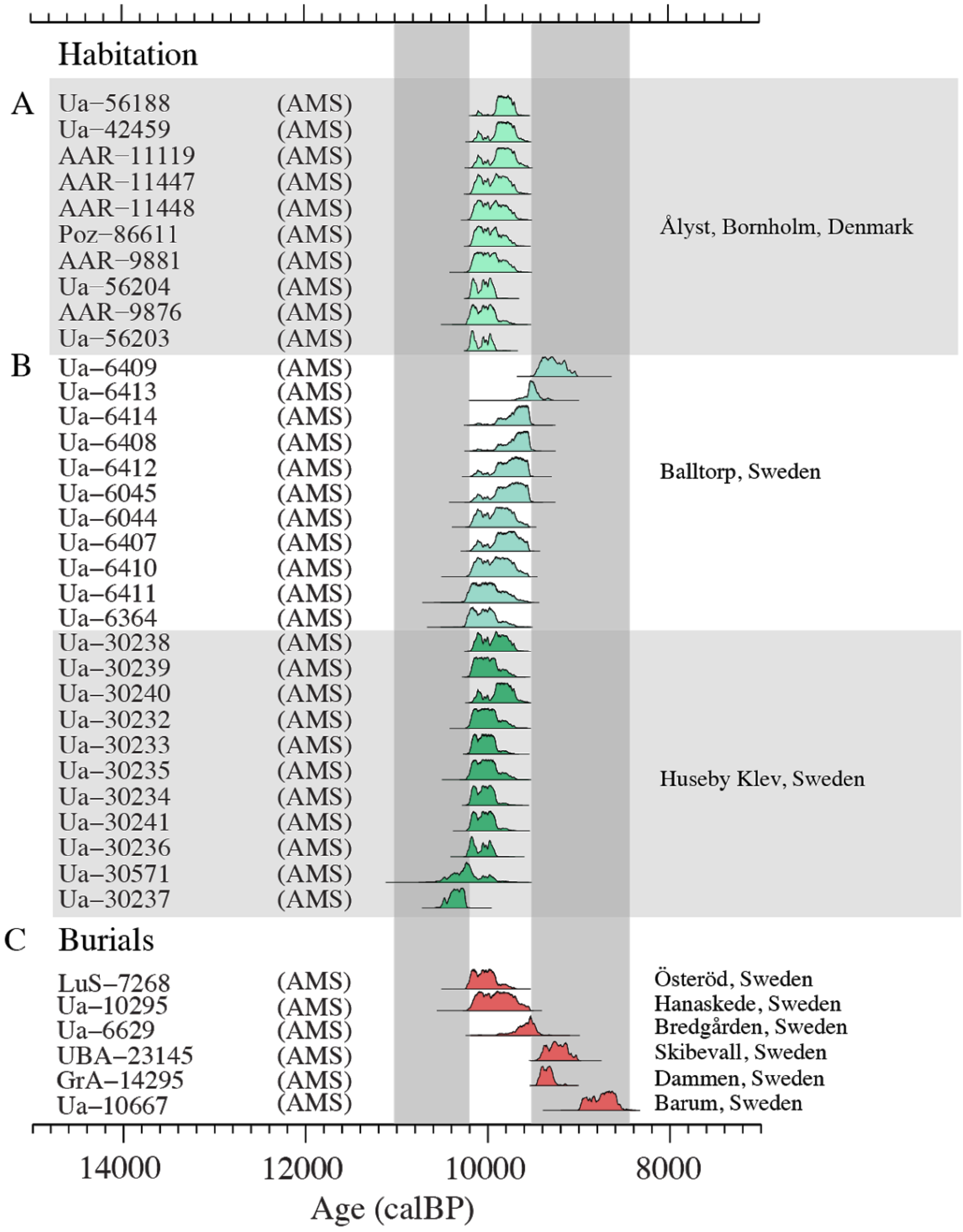

Supplementary Figure 24. Radiocarbon dates from the Early Mesolithic from the Danish island of Bornholm and Southern Sweden, A) radiocarbon dates from Bornholm associated with habitation, B) radiocarbon dates from Southern Sweden associated with habitation, C) burial from context associated with habitation.

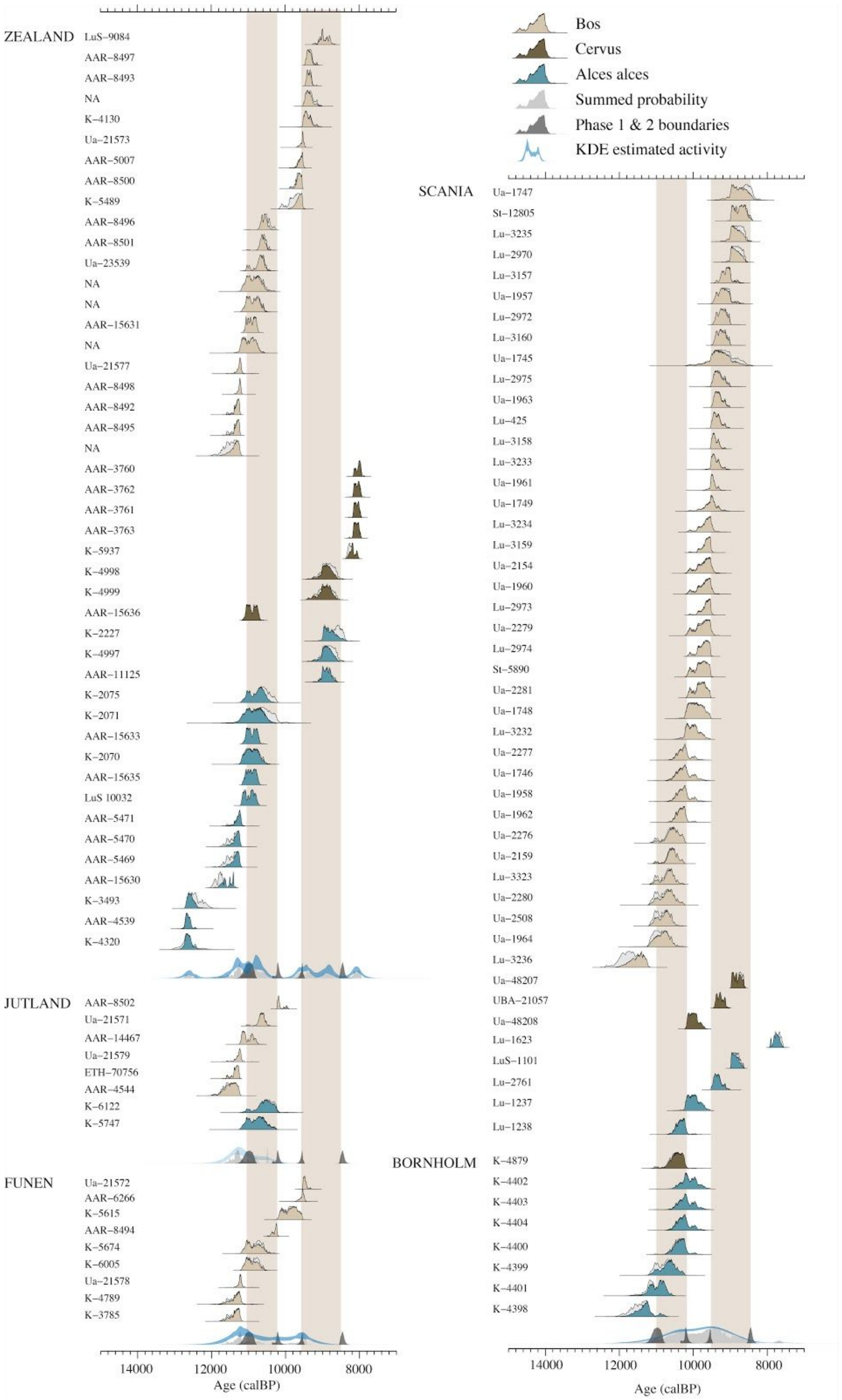

Supplementary Figure 25. Radiocarbon dates of faunal remains of elk, red deer, aurochs, and bison shown in different colours. Phase model applied to each taxonomic class by region. KDE model under each region denotes activity.

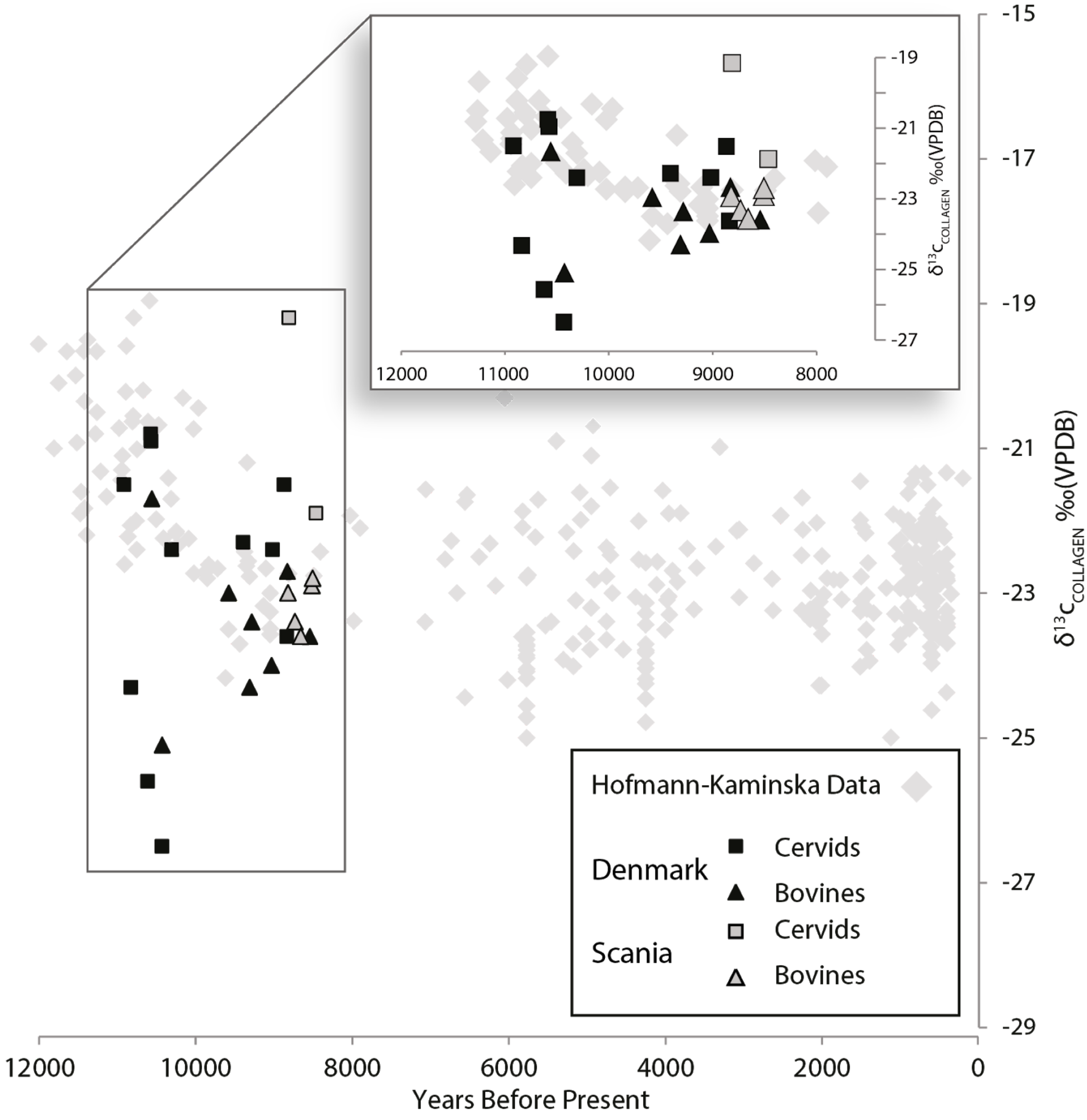

Supplementary Figure 26. Collagen $\delta^{13}C$ measurements obtained from bone points from Denmark and Rönneholm, plotted against data from Hofmann-Kaminska [30](see Supplementary Dataset SI1 for actual measurements).

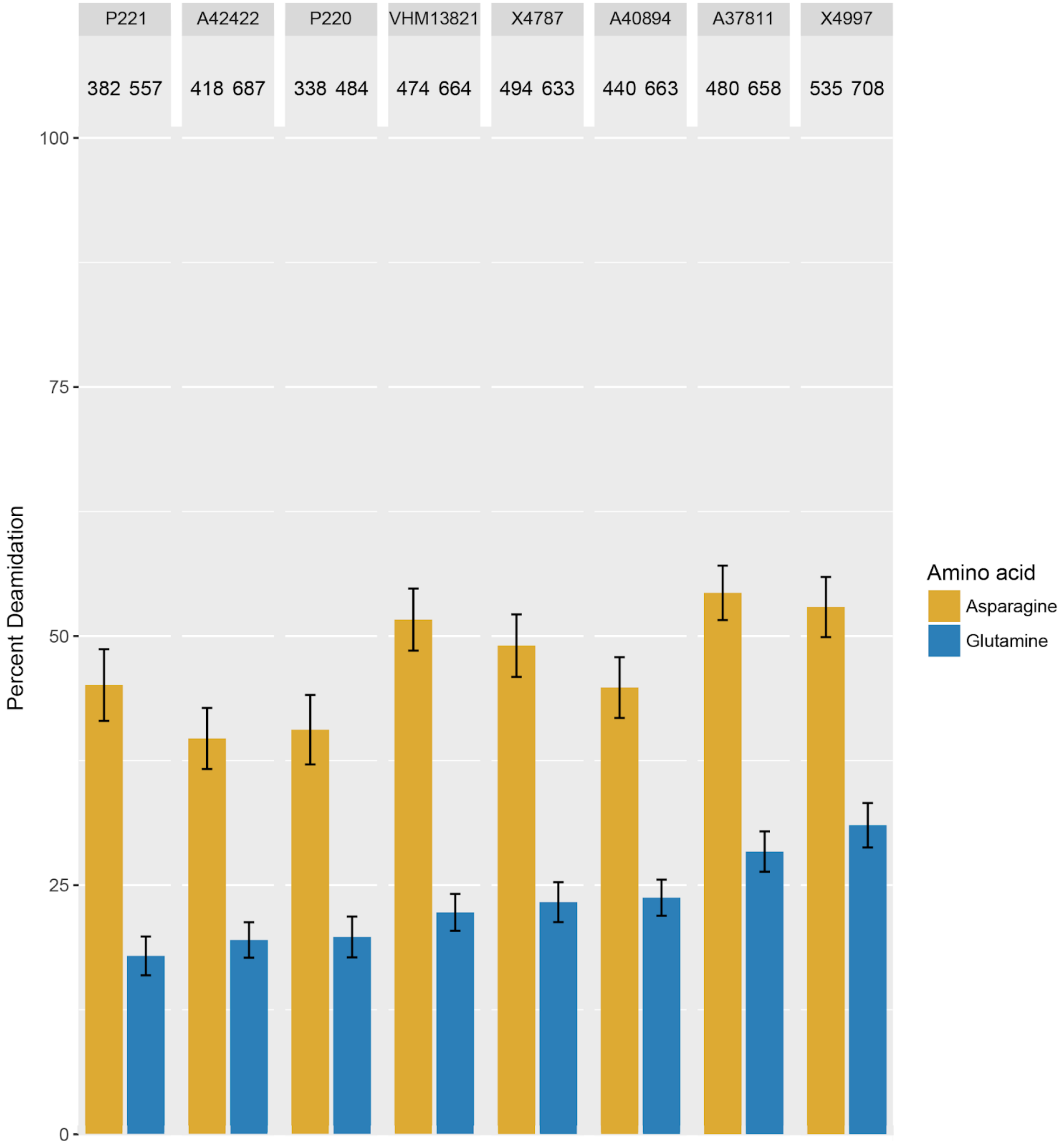

Supplementary Figure 27. The overall percentage of deamidation for asparagine (N) and glutamine (Q) residues for the authentic bone proteins from the bone points (A37811, A40874, A42422, and VHM13821) and the standard elk (P220, P221) and red deer (X4787, X4997) samples. Error bars represent standard deviation and numbers above each bar represent the number of peptides the calculation is based on.

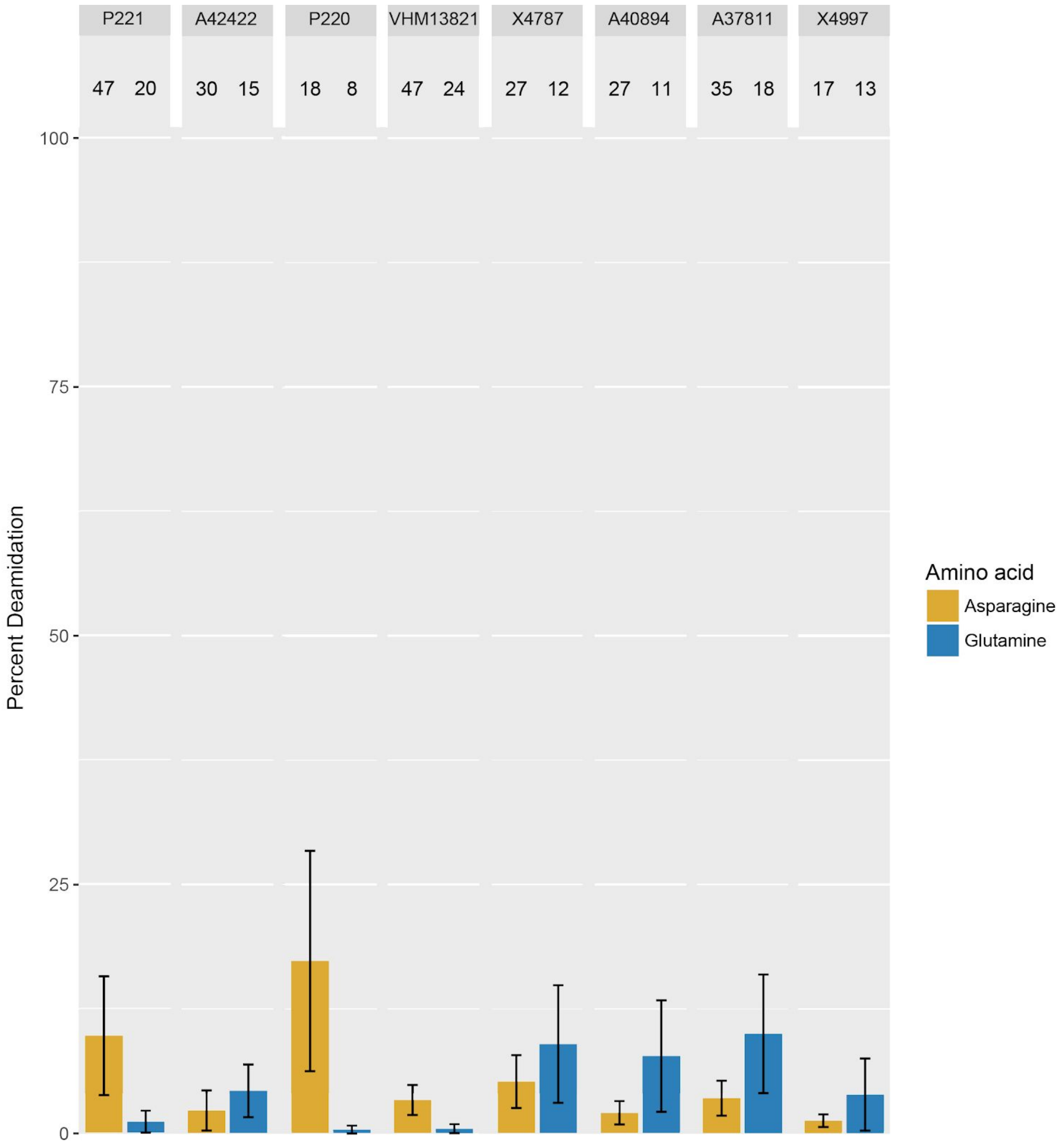

Supplementary Figure 28. Overall percentage of deamidation for asparagine (N) and glutamine (Q) residues for the contaminant proteins from the bone points (A37811, A40874, A42422, and VHM13821) and the standard elk (P220, P221) and red deer (X4787, X4997) samples. Error bars represent standard deviation and numbers above each bar represent the number of peptides the calculation is based on.

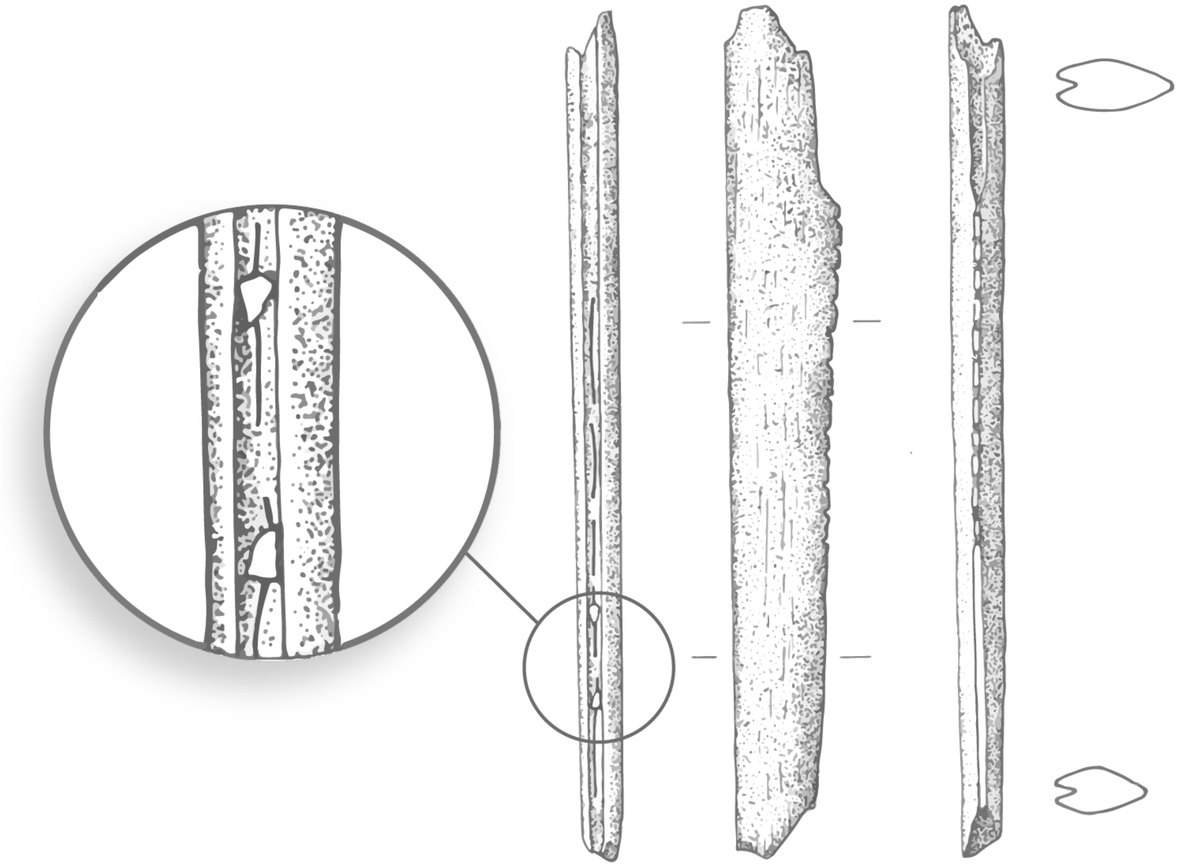

Supplementary Figure 29. Slotted bone point from Fulge Å (AAR-11949, 8360±55), Store Åmose (drawn by Kurt Petersen, Kalundborg Museum in cooperation with Anders Fischer).

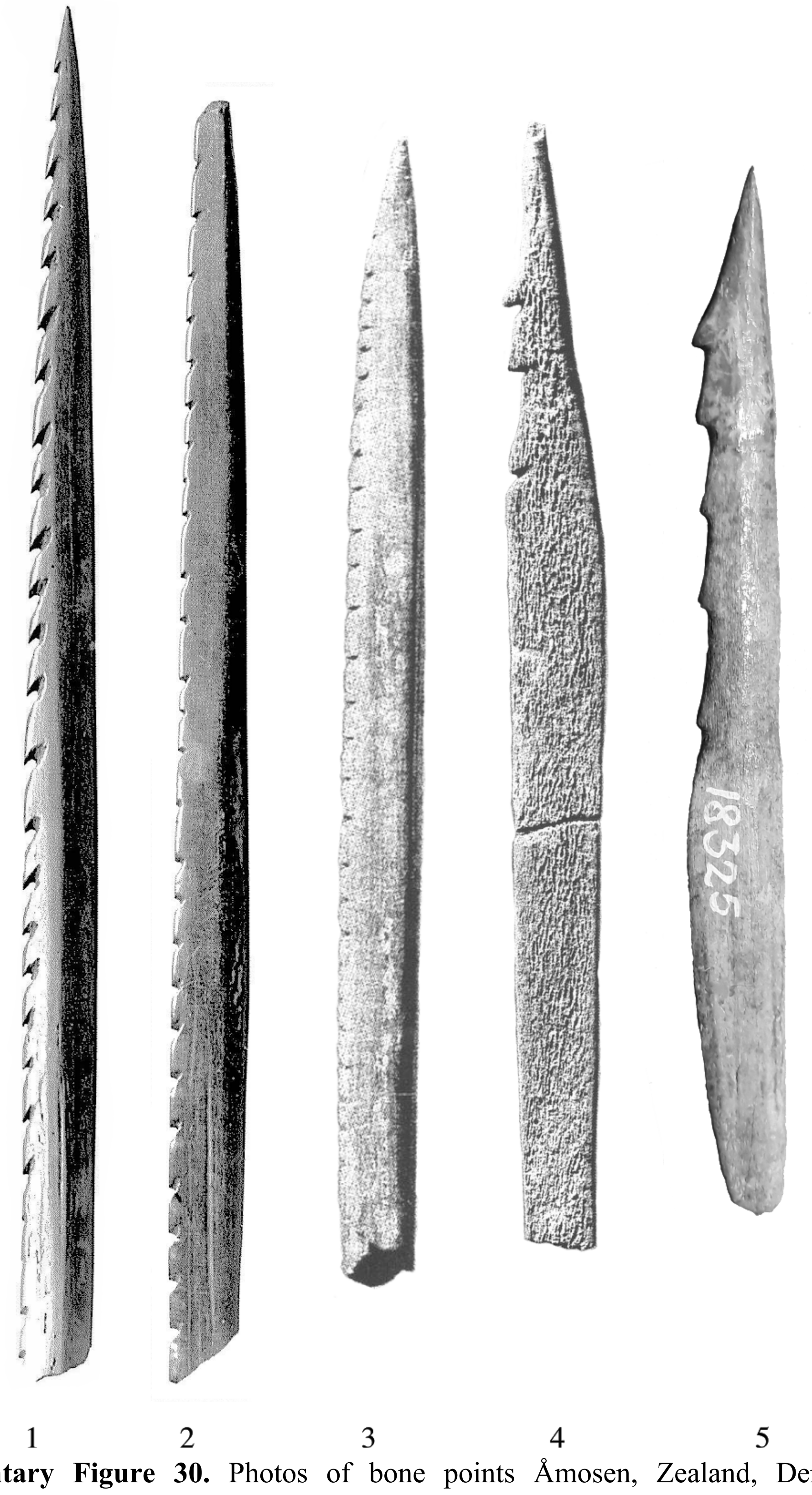

1 2 3 4 5

Supplementary Figure 30. Photos of bone points Åmosen, Zealand, Denmark, only radiocarbon dated. 1: Mørke Enge (Lyster 7), 2: Mørke Enge (Lyster 11), 3: Sønderrød (A44205), 4: Ulkestrup Øst IV (A47608), 5: Ulkestrup Lyng (KAM-18325). No scale provided on original photos.

Supplementary Tables

Supplementary Table 1. Unique tryptic peptide sequences between red deer and elk collagen type 1

Marker Name	Location	Species	Sequence	Blast search matches	LC-MS/MS Marker	ZooMS Marker
A1T66/67	733-756 COL1a1	elk	GETGPAGR **A**GEVGPPGPPGPAGEK	only collagen	yes	no
		red deer	GETGPAGR **P**GEVGPPGPPGPAGEK	only collagen		yes - missed cleavage before P at m/z 2216
A1T74	849-855 COL1a1	elk	DGSPG**P**K	KFY28177.1 (*Pseudogymnoascus sp*), VDL62941.1 (*Hymenolepis diminuta*)	no	no
		red deer	DGSPG**A**K	collagen and fish proteins		
A2T40	430-443 COL1a2	elk	GAPGPDGNNGAQGPPG**L**QGVQGGK	only collagen	no	no
		red deer	GAPGPDGNNGAQGPPG**P**QGVQGGK	WP_139160575.1 (*Acinetobacter baumannii*), WP_139109296.1 (*Klebsiella pneumoniae*)		
A2T51	574-582 COL1a2	elk	GD**I**GSPGR	WP_026910359.1 (*Patulibacter minatonensis*), RIK66337.1 (*Planctomycetes bacterium*)	no	no
		red deer	GD**V**GSPGR	MAW74128.1 (*Gemmatimonadetes bacterium*), OTB08864.1 (*Hypoxylon sp.*)		
A2T66	741-757 COL1a2	elk	**T**GETGASGPPGFAGEK	only collagen	yes	no
		red deer	**S**GETGASGPPGFAGEK	only collagen		

Supplementary Table 2. Species diagnostic peptides found in bone points and reference material. Based on search of LC-MS/MS raw files in MaxQuant (v. 1.6.3.4). 2 spectra in A40894 and 1 in A42422 hit *Alces alces* specific peptides; however, the specific amino acids are not individually covered by b or y ions. Due to the relatively small abundance of these peptides compared to the *Cervus elaphus* peptides found in these samples, these hits are most likely false positives possibly due to unknown modifications or come from sample cross-contamination.

*Based on best spectra if multiple
**Identified by MaxQuant's second peptide search
X- species-specific amino acid
Q - deamidation
(gl)- glu -> pyro-Glu

Sample	Sequence (No. of Hydroxyproline)	Length (aa)	Missed cleavages	Mass (Da)	Charge	MQ score*	No. of Matched spectra
**P220** - Elk (*Alces alces*) reference material	AGEVGPPGPPGPA (2)	13	0	1133.5353	1	194.90	1
	AGEVGPPGPPGPAGEK (1)	16	0	1431.6994	2	136.82	1
	AGEVGPPGPPGPAGEK (2)	16	0	1447.6943	2	257.75	4
	AGEVGPPGPPGPAGEK (3)	16	0	1463.6892	2	179.72	2
	AGEVGPPGPPGPAGEKG (2)	17	1	1504.7158	2	174.05	1
	AGEVGPPGPPGPAGEKGAPG (3)	20	1	1745.8220	2	166.62	1
	AGEVGPPGPPGPAGEKGAPG (4)	20	1	1761.8170	2	162.72	1
	AGEVGPPGPPGPAGEKGAPGADGPA (2)	25	1	2141.0025	2	333.13	1
	AGEVGPPGPPGPAGEKGAPGADGPA (3)	25	1	2156.9974	2	329.16	2
	AGEVGPPGPPGPAGEKGAPGADGPA (4)	25	1	2172.9924	2	306.72	1
	EGLRGPRGDQGPVGRT (0)	16	3	1650.855	2	191.69	1
	EGLRGPRGD**Q**GPVGRT (0)	16	3	1651.839	2	137.81	1
	GD**Q**GPVGRT (0)	9	1	886.41446	2	93.467	1
	GPRGDQGPVGRT (0)	12	2	1195.6058	2;3	136.86	2
	GPRGD**Q**GPVGRT (0)	12	2	1196.5898	2;3	155.26	2
	GRAGEVGPPGPPGPAGEK (2)	18	1	1660.8169	2	202.63	1
	TGETGASGPPGFAGEK (1)	16	0	1477.6685	2	260.81	1
**P221** - Elk (*Alces alces*) reference material	AGEVGPPGPPG (2)	11	0	965.44543	2	102.30	1
	AGEVGPPGPPGPAGEK (1)	16	0	1431.6994	2	169.82	1
	AGEVGPPGPPGPAGEK (2)	16	0	1447.6943	2	237.08	3
	AGEVGPPGPPGPAGEK (3)	16	0	1463.6892	2	150.65	2
	AGEVGPPGPPGPAGEKG (2)	17	1	1504.7158	2	184.43	1
	AGEVGPPGPPGPAGEKGAPG (2)	20	1	1729.8271	2	217.57	1

	AGEVGPPGPPGPAGEKGAPG (3)	20	1	1745.8220	2	145.81	1
	AGEVGPPGPPGPAGEKGAPG (4)	20	1	1761.8170	2	166.62	1
	AGEVGPPGPPGPAGEKGAPGADGPA (2)	25	1	2141.0025	2	328.92	1
	AGEVGPPGPPGPAGEKGAPGADGPA (3)	25	1	2156.9974	2	261.91	2
	AGEVGPPGPPGPAGEKGAPGADGPA (4)	25	1	2172.9924	2	303.44	1
	EGLRGPRGDQGPVGRT (0)	16	3	1650.855	2	199.30	1
	EGLRGPRGD**Q**GPVGRT (0)	16	3	1651.839	2;3;4	138.97	3
	(gl)-EGLRGPRGD**Q**GPVGRT (0)	16	3	1633.8285	3	105.77	1
	GD**Q**GPVGRT	9	1	886.41446	2	100.02	1
	GPRGDQGPVGRT (0)	12	2	1195.6058	2;3	197.94	2
	GPRGD**Q**GPVGRT (0)	12	2	1196.5898	2	160.91	1
	GRAGEVGPPGPPGPAGEK (2)	18	1	1660.8169	2	215.15	1
	GPRGDQGPVGRTGETGASGPPGF (1)	23	2	2169.0199	3	96.881	1
	TGETGASGPPGFAGEK (1)	16	0	1477.6685	2	273.02	1
	TGETGASGPPGFAGEK (2)	16	0	1493.6634	2	122.86	1
**X4787** - Red Deer (*Cervus elaphus*) reference material	GETGPAGRPGE (1)	11	1	1042.468	2	106.40	1
	GETGPAGRPGEVGPPGPPGPA (3)	21	1	1900.8915	2	220.85	1
	GETGPAGRPGEVGPPGPPGPAGEK (3)	24	1	2215.0505	2;3	326.78	3
	GETGPAGRPGEVGPPGPPGPAGEK (4)	24	1	2231.0455	2;3	219.95	2
	GETGPAGRPGEVGPPGPPGPAGEKG (3)	25	2	2272.072	3	100.23	1
	GRPGEVGPPGPPGPAGEK (3)	18	1	1702.8275	2;3	278.30	4
	GRPGEVGPPGPPGPAGEK (4)	18	1	1718.8224	2	147.35	1
	PGEVGPPGPPGPAGEK (3)	16	0	1489.7049	2	257.16	3
	PGEVGPPGPPGPAGEK (4)	16	0	1505.6998	2	222.20	2
	SGETGASGPPGF (1)	12	0	1078.4567	2	171.75	1
	SGETGASGPPGFAGEK (0)	16	0	1447.6579	2	174.90	1
	SGETGASGPPGFAGEK (1)	16	0	1463.6529	2	234.79	2
	SGETGASGPPGFAGEK (2)	16	0	1479.6478	2	99.752	1
	GPRGDQGPVGRSGETGASGPPGF (1)	23	2	2155.0043	3	136.96	1
**X4997** - Red Deer (*Cervus elaphus*) reference material	EGLRGPRGDQGPVGRS (0)	16	3	1636.8394	3	82.709	1
	EGLRGPRGD**Q**GPVGRS (0)	16	3	1637.8234	3	72.341	1
	GETGPAGRPG (1)	10	1	913.42536	2	98.629	1
	GETGPAGRPGEVGPPGPPGPAGEK (2)	24	1	2199.0556	3	107.09	1
	GETGPAGRPGEVGPPGPPGPAGEK (3)	24	1	2215.0505	2;3	272.19	3
	GETGPAGRPGEVGPPGPPGPAGEK (4)	24	1	2231.0455	2;3	315.58	2
	GRPGEVGPPGPPGPAGEK (3)	18	1	1702.8275	2;3	278.68	2
	GRPGEVGPPGPPGPAGEK (4)	18	1	1718.8224	2;3	139.66	2
	PGEVGPPGPPGPAGEK (2)	16	0	1473.71	2	245.85	1
	PGEVGPPGPPGPAGEK (3)	16	0	1489.7049	2;3	295.20	5

	PGEVGPPGPPGPAGEK (4)	16	0	1505.6998	2	237.67	2
	SGETGASGPPGFAGEK (0)	16	0	1447.6579	2	177.10	1
	SGETGASGPPGFAGEK (1)	16	0	1463.6529	2	234.63	2
	SGETGASGPPGFAGEK (2)	16	0	1479.6478	2	143.97	1
	GPRGDQGPVGRSGETGASGPPGF (1)	23	2	2155.0043	3	175.33	1
**A37811** - Preboral bone point (*Alces alces*)	AGEVGPPG (1)	8	0	698.32352	2	62.338	1
	AGEVGPPGP (1)	9	0	795.37628	2	67.741	1
	AGEVGPPGPPG (2)	11	0	965.44543	2	124.34	1
	AGEVGPPGPPGPA (2)	13	0	1133.5353	2	137.62	1
	AGEVGPPGPPGPAG (2)	14	0	1190.5568	2	117.04	1
	AGEVGPPGPPGPAGEK (1)	16	0	1431.6994	2	178.36	1
	AGEVGPPGPPGPAGEK (2)	16	0	1447.6943	2;3	259.47	7
	AGEVGPPGPPGPAGEK (3)	16	0	1463.6892	2	189.43	6
	AGEVGPPGPPGPAGEK (4)	16	0	1479.6842	2	103.44	2
	AGEVGPPGPPGPAGEKG (2)	17	1	1504.7158	2	85.536	1
	AGEVGPPGPPGPAGEKG (3)	17	1	1520.7107	2	117.20	1
	AGEVGPPGPPGPAGEKGA (3)	18	1	1591.7478	2	143.42	1
	RAGEVGPPGPPGPAGEK (3)	17	1	1619.7903	3	69.74	1
	TGETGASGPPGFAGEK (1)	16	0	1477.6685	2	243.40	1
	TGETGASGPPGFAGEK (2)	16	0	1493.6634	2	128.28	2
	TGETGASGPPGFAGEKG (1)	17	1	1534.69	2	153.75	1
**A40894** - Boral bone point (*Cervus elaphus*)	ETGPAGRPGEVGPPGPPGPAGEK (4)	23	1	2174.024	3	78.837	1
	GETGPAGRPG (1)	10	1	913.42536	2	69.905	1
	GETGPAGRPGE (1)	11	1	1042.468	2	136.63	1
	GETGPAGRPGEV (1)	12	1	1141.5364	2	72.848	1
	GETGPAGRPGEVG (1)	13	1	1198.5578	2	199.68	2
	GETGPAGRPGEVGP (1)	14	1	1295.6106	2	223.87	1
	GETGPAGRPGEVGPP (2)	15	1	1408.6583	2	173.37	1
	GETGPAGRPGEVGPPG (2)	16	1	1465.6797	2	114.24	1
	GETGPAGRPGEVGPPGP (2)	17	1	1562.7325	2	122.52	1
	GETGPAGRPGEVGPPGPPG (3)	19	1	1732.8016	2	218.11	2
	GETGPAGRPGEVGPPGPPGPAG (3)	22	1	1957.913	2	283.10	1
	GETGPAGRPGEVGPPGPPGPAGEK (3)	24	1	2215.0505	2;3	285.34	3
	GETGPAGRPGEVGPPGPPGPAGEK (4)	24	1	2231.0455	2;3	276.03	2
	GETGPAGRPGEVGPPGPPGPAGEK (5)	24	1	2247.0404	3	179.50	5
	GETGPAGRPGEVGPPGPPGPAGEKG (4)	25	2	2288.0669	3	143.25	1
	GPAGRPGEVGPPGPPGPAGEK (4)	21	1	1943.9337	3	123.53	1
	GRPGEVGPPGPPGPAGEK (4)	18	1	1718.8224	3	67.995	1
	PAGRPGEVGPPGPPGPAGEK (4)	20	1	1886.9123	3	121.63	1
	PGEVGPPGPPG (3)	11	0	1007.456	2	95.352	1
	PGEVGPPGPPGPAGEK (2)	16	0	1473.71	2	132.99	1

	PGEVGPPGPPGPAGEK (3)	16	0	1489.7049	2	257.76	3
	PGEVGPPGPPGPAGEK (4)	16	0	1505.6998	2	236.11	3
	RPGEVGPPGPPGPAGEK (4)	17	1	1661.8009	3	63.374	1
	TGPAGRPGEVGPPGPPGPAGEK (4)	22	1	2044.9814	3	109.39	1
	SGETGASGPPGFAGEK (0)	16	0	1447.6579	2	134.56	1
	SGETGASGPPGFAGEK (1)	16	0	1463.6529	2	223.31	4
	SGETGASGPPGFAGEKG (1)	17	1	1520.6743	2	104.49	1
**A42422** - Boral bone point (*Cervus elaphus*)	GETGPAGRPGE (1)	11	1	1042.468	2	145.89	1
	GETGPAGRPGEVG (1)	13	1	1198.5578	2	179.42	1
	GETGPAGRPGEVGP (1)	14	1	1295.6106	2	103.35	1
	GETGPAGRPGEVGPPG (2)	16	1	1465.6797	2	106.26	1
	GETGPAGRPGEVGPPGP (2)	17	1	1562.7325	2	130.79	1
	GETGPAGRPGEVGPPGPPG (3)	19	1	1732.8016	2	225.33	2
	GETGPAGRPGEVGPPGPPGPAG (3)	22	1	1957.913	2	314.55	1
	GETGPAGRPGEVGPPGPPGPAGEK (3)	24	1	2215.0505	3	210.32	4
	GETGPAGRPGEVGPPGPPGPAGEK (4)	24	1	2231.0455	2;3	293.20	5
	GETGPAGRPGEVGPPGPPGPAGEK (5)	24	1	2247.0404	3	142.32	1
	GRPGEVGPPGPPGPAGEK (4)	18	1	1718.8224	3	64.249	1
	PAGRPGEVGPPGPPGPAGEK (3)	20	1	1870.9173	3	65.425	1
	PGEVGPPGPPG (3)	11	0	1007.456	2	99.375	1
	PGEVGPPGPPGPAG (3)	14	0	1232.5673	2	153.08	1
	PGEVGPPGPPGPAGEK (2)	16	0	1473.71	2	164.68	1
	PGEVGPPGPPGPAGEK (3)	16	0	1489.7049	2	297.50	3
	PGEVGPPGPPGPAGEK (4)	16	0	1505.6998	2	222.20	2
	RPGEVGPPGPPGPAGEK (4)	17	1	1661.8009	3	63.145	1
	SGETGASGPPGFAGEK (0)	16	0	1447.6579	2	168.02	1
	SGETGASGPPGFAGEK (1)	16	0	1463.6529	2	231.67	4
	SGETGASGPPGFAGEK (2)	16	0	1479.6478	2	100.47	1
**VHM13821** - Preboral bone point (*Alces alces*)	AGEVGPPG** (1)	8	0	698.32352	1	85.306	1
	AGEVGPPGPPG (2)	11	0	965.44543	2	120.45	2
	AGEVGPPGPPGPA (2)	13	0	1133.5353	2	110.67	1
	AGEVGPPGPPGPAGEK (1)	16	0	1431.6994	2	147.10	1
	AGEVGPPGPPGPAGEK (2)	16	0	1447.6943	2;3	248.56	7
	AGEVGPPGPPGPAGEK (3)	16	0	1463.6892	2;3	172.60	7
	AGEVGPPGPPGPAGEK (4)	16	0	1479.6842	2	154.67	3
	AGEVGPPGPPGPAGEKG (2)	17	1	1504.7158	2	158.34	1
	AGEVGPPGPPGPAGEKG (3)	17	1	1520.7107	2	135.26	2
	TGETGASGPPGFAGEK (0)	16	0	1461.6736	2	173.76	1
	TGETGASGPPGFAGEK (1)	16	0	1477.6685	2	250.67	3
	TGETGASGPPGFAGEK (2)	16	0	1493.6634	2	162.68	3

References

1. Andersen, K. *Stenalder bebyggelsen i den Vestsjællandske Åmose*. (Fredningsstyrelsen, 1983).
2. Fischer, A. Stone Age Åmose. Stored in museums and preserved in the living bog. in *Bog Bodies, Sacred Sites and Wetland Archaeology* (eds. Coles, B., Coles, J. & Jørgensen, M. S.) vol. 8 (Exeter: WARP, 1999).
3. Mathiassen, T., Troels-Smith, J. & Degerbøl, M. Stenalderbopladser i Aamosen. (1943).
4. Jørgensen, S. *Early Postglacial in Aamosen: Geological and Pollen-analytical Investigations of Maglemosian Settlements in the West-Zealand Bog Aamosen*. (Reitzel, 1963).
5. Noe-Nygaard, N. Sedimentary, geochemical and ecological evolution of a Lateglacial--Postglacial lacustrine basin: lakelevel and climatic influence on flora, fauna and human population (Aamosen, Denmark). *Fossils and Strata* **37**, 1–436 (1995).
6. Noe-Nygaard, N., Abildtrup, C. H., Albrechtsen, T., Gotfredsen, A. B. & Richter, J. Palæobiologiske, sedimentologiske og geokemiske undersøgelser af Sen Weichel og Holocæne aflejringer i Store Åmose, Danmark. *Geologisk tidsskrift* **2**, 1–65 (1998).
7. Althin, C.-A. *The Chronology of the Stone Age Settlement of Scania, Sweden: the mesolithic settlement. I.* (R. Habelt, 1954).
8. Larsson, L. *Ageröd V: An Atlantic bog site in central Scania*. vol. No 12. (Institute of Archaeology, University of Lund, 1983).
9. Larsson, L. *Ageröd I:B - Ageröd I:D: A study of early Atlantic settlement in Scania*. vol. No 12 (C. W. K. Gleerup, 1978).
10. Sjöström, A. *Slabälta 1 - en boplats från sen maglemosetid vid Ageröds mosse: Arkeologisk undersökning 2012: Munkarp 2:3, Munkarp socken, Höörs kommun, Skåne*. vol. 6 (2013).
11. Larsson, L., Sjöström, A. & Nilsson, B. Lost at the bottom of the lake. Early and Middle Mesolithic leister points found in the bog Rönneholms Mosse, southern Sweden. in *Working at the sharp end: From bone and antler to Early Mesolithic life in Northern Europe* (eds. Groß, D., Lübke, H., Meadows, J. & Jantzen, D.) 1–8 (Wacholtz, 2019).
12. Nilsson, T. Die pollenanalytische Zonengliederung der spät- und postglazialen Bildungen Schonens. *Geologiska Föreningen i Stockholm Förhandlingar* **57**, 385–562 (1935).
13. Nilsson, T. Entwicklungsgeschichtliche Studien in Ageröds mosse, Schonen. *Lunds Universitets Årsskrift N. F. Avd. 2* **59**, (1964).
14. Nilsson, T. Standardpollendiagramme und C14-Datierungen aus dem Ageröds Mosse im mittleren Schonen. *LUNDS UNIVERSITETS ÄRSSKRIFT. NF Avd. 2. Bd 59. Nr 7.* (1964).
15. Nilsson, T. *Pollenanalytische Datierung mesolithischer Siedlungen im Randgebiet des Ageröds Mosse im mittleren Schonen*. vol. 16 (Acta Univ. Lund, 1967).
16. Fischer, A. At the border of human habitat. The Late Palaeolithic and Early Mesolithic in Scandinavia. in *The Earliest Settlement of Scandinavia and its relationship with neighbouring areas* (ed. Larsson, L.) 157–176 (Almquist & Wiksell, 1996).
17. Fischer, A. Tissø og Amoserne som trafikforbindelse og kultsted i stenalderen. *Historisk Samfund for Holbæk Amt* 27–44 (2003).

18. Fischer, A. Food for Feasting? An evaluation of explanations of the neolithisation of Denmark and southern Sweden. in *The Neolithisation of Denmark – 150 Years of Debate* (eds. Fischer, A. & Krisiansen, K.) 343–393 (J.R. Collis, 2002).
19. Fischer, A. *et al.* Coast–inland mobility and diet in the Danish Mesolithic and Neolithic: evidence from stable isotope values of humans and dogs. *J. Archaeol. Sci.* **34**, 2125–2150 (2007).
20. van Doorn, N. L., Hollund, H. & Collins, M. J. A novel and non-destructive approach for ZooMS analysis: ammonium bicarbonate buffer extraction. *Archaeol. Anthropol. Sci.* **3**, 281 (2011).
21. Buckley, M., Collins, M., Thomas-Oates, J. & Wilson, J. C. Species identification by analysis of bone collagen using matrix-assisted laser desorption/ionisation time-of-flight mass spectrometry. *Rapid Commun. Mass Spectrom.* **23**, 3843–3854 (2009).
22. Strohalm, M., Kavan, D., Novák, P., Volný, M. & Havlícek, V. mMass 3: a cross-platform software environment for precise analysis of mass spectrometric data. *Anal. Chem.* **82**, 4648–4651 (2010).
23. Kirby, D. P., Buckley, M., Promise, E., Trauger, S. a. & Holdcraft, T. R. Identification of collagen-based materials in cultural heritage. *Analyst* **138**, 4849–4858 (2013).
24. Mackie, M. *et al.* Palaeoproteomic Profiling of Conservation Layers on a 14th Century Italian Wall Painting. *Angew. Chem. Int. Ed Engl.* **57**, 7369–7374 (2018).
25. Cox, J. & Mann, M. MaxQuant enables high peptide identification rates, individualized ppb-range mass accuracies and proteome-wide protein quantification. *Nature Biotechnology* **26**, 1367–1372 (2008).
26. Welker, F. *et al.* Palaeoproteomic evidence identifies archaic hominins associated with the Châtelperronian at the Grotte du Renne. *Proc. Natl. Acad. Sci. U. S. A.* **113**, 11162–11167 (2016).
27. Rodriguez, J., Gupta, N., Smith, R. D. & Pevzner, P. A. Does trypsin cut before proline? *J. Proteome Res.* **7**, 300–305 (2008).
28. DeNiro, M. J. Postmortem preservation and alteration of in vivo bone collagen isotope ratios in relation to palaeodietary reconstruction. *Nature* **317**, 806–809 (1985).
29. Bocherens, H., Hofman-Kamińska, E., Drucker, D. G., Schmölcke, U. & Kowalczyk, R. European bison as a refugee species? Evidence from isotopic data on Early Holocene bison and other large herbivores in northern Europe. *PLoS One* **10**, e0115090 (2015).
30. Hofman-Kamińska, E. *et al.* Adapt or die-Response of large herbivores to environmental changes in Europe during the Holocene. *Glob. Chang. Biol.* 1–16 (2019).
31. Björck, S. A review of the history of the Baltic Sea, 13.0-8.0 ka BP. *Quat. Int.* **27**, 19–40 (1995).
32. Bennike, O., Andreasen, M. S., Jensen, J. B., Moros, M. & Noe-Nygaard, N. Early Holocene sea-level changes in Øresund, southern Scandinavia. *Geological Survey of Denmark and Greenland Bulletin* **26**, 29–32 (2012).
33. Bennike, O., Jensen, J. B., Lemke, W., Kuijpers, A. & Lomholt, S. Late- and postglacial history of the Great Belt, Denmark. *Boreas* **33**, 18–33 (2004).
34. Bennike, O. & Jensen, J. B. Postglacial, relative shore-level changes in Lillebælt, Denmark. *Geological Survey of Denmark and Greenland Bulletin* **23**, 37–40 (2011).
35. Wiberg-Larsen, P., Bennike, O., Jensen, J. B. O. & Lemke, W. Trichoptera remains from early Holocene river deposits in the Great Belt, Denmark. *Boreas* **30**, 299–306 (2001).
36. Jensen, J. B., Kuijpers, A., Bennike, O. & Lemke, W. Balkat: The Baltic Sea without frontiers. *Geologi, nyt fra GEUS* **4**, 2–20 (2002).

37. Lam, Y. M., Chen, X. & Pearson, O. M. Intertaxonomic Variability in Patterns of Bone Density and the Differential Representation of Bovid, Cervid, and Equid Elements in the Archaeological Record. *Am. Antiq.* **64**, 343–362 (1999).
38. Gron, K. J. Body Part Representation, Fragmentation and Patterns of Ertebølle Deer Exploitation in Northwest Zealand, Denmark. *Int. J. Osteoarchaeol.* **25**, 722–732 (2015).
39. Osipowicz, G. Bone and antler: softening techniques in prehistory of the North Eastern part of Polish Lowlands in the light of experimental archaeology and micro trace analysis. *euroREA. Journal for (Re)construction and Experiment in Archaeology* **4**, 11–20 (2007).
40. Gaillard, M.-J. Postglacial paleoclimatic changes in Scandinavia and Central Europe. A tentative correlation based on studies of lake-level fluctuations. *Ecol. Mediterr.* **11**, 159–175 (1985).
41. Gedda, B. Environmental and climatic aspects of the early to mid Holocene calcareous tufa and land mollusc fauna in southern Sweden. (Lund University, 2001).
42. Digerfeldt, G., Björck, S., Hammarlund, D. & Persson, T. Reconstruction of Holocene lake-level changes in Lake Igelsjön, southern Sweden. *GFF* **135**, 162–170 (2013).
43. Welinder, S. The concept of'ecology'in Mesolithic research. *The Early Postglacial Settlement of Northern Europe. London: Duckworth* 11–26 (1978).
44. Newell, R. R. Making Cultural Ecology Relevant to Mesolithic Research: II. Restocking the Larder of the Later Mesolithic of Zealand, Denmark. in *Contributions to the Mesolithic in Europe* (eds. Vermeersch, P. M. & Peer, P. V.) 53–69 (Leuven University Press, 1990).
45. Magnell, O. Tracking Wild Boar and Hunters : Osteology of Wild Boar in Mesolithic South Scandinavia. (Lund University, 2006).
46. Aaris-Sørensen, K. Depauperation of the Mammalian Fauna of the Island of Zealand during the Atlantic Period. *Vidensk. Meddr Dansk Naturh. Foren.* **142**, 131–138 (1980).
47. Christensen, C. The Littorina Transgressions in Denmark. *Man and sea in the Mesolithic* 15–21 (1995).
48. Iversen, J. *The Development of Denmark's Nature Since the Last Glacial*. (C. A. Reitzels Forlag, 1973).
49. Schulz, E. & Kaiser, T. M. Feeding strategy of the Urus Bos primigenius BOJANUS, 1827 from the Holocene of Denmark. *Cour. Forsch.-Inst. Senckenberg* **259**, 155–164 (2007).
50. Lepiksaar, J. The Holocene history of theriofauna in Fennoscandia and Baltic countries. *Striae* **24**, 51–70 (1986).
51. Hofmann, R. R. Evolutionary steps of ecophysiological adaptation and diversification of ruminants: a comparative view of their digestive system. *Oecologia* **78**, 443–457 (1989).
52. Gron, K. J. *et al.* Strontium isotope evidence of early Funnel Beaker Culture movement of cattle. *Journal of Archaeological Science: Reports* **6**, 248–251 (2016).
